# Facile synthesis of *N*- (4-bromophenyl)-1- (3-bromothiophen-2-yl)methanimine derivatives via Suzuki cross-coupling reaction: their characterization and DFT studies

**DOI:** 10.1186/s13065-018-0451-0

**Published:** 2018-07-17

**Authors:** Komal Rizwan, Nasir Rasool, Ravya Rehman, Tariq Mahmood, Khurshid Ayub, Tahir Rasheed, Gulraiz Ahmad, Ayesha Malik, Shakeel Ahmad Khan, Muhammad Nadeem Akhtar, Noorjahan Banu Alitheen, Muhammad Nazirul Mubin Aziz

**Affiliations:** 1 Department of Chemistry, Government College University, Faisalabad, 38000 Pakistan; 2 Department of Chemistry, Government College Women University, Faisalabad, Pakistan; 3 Department of Chemistry, COMSATS Institute of Information Technology, University Road, Tobe Camp, Abbottabad, 22060 Pakistan; 4 The School of Chemistry & Chemical Engineering, State Key Laboratory of Metal Matrix Composites, Shanghai Jiao Tong University, 800 Dongchuan Road, Shanghai, 200240 China; 5 Faculty of Industrial Sciences & Technology, University Malaysia Pahang, LebuhrayaTun, Razak, 26300 Kuantan, Pahang Malaysia; 6 Deparment of Cell and Molecular Biology, Faculty of Biotechnology and Biomolecular Science, University Putra Malaysia, 43400 Serdang, Selangor DarulEhsan Malaysia

**Keywords:** Imines, Thiophene, Suzuki coupling, Density functional theory, Computational, Reactivity

## Abstract

A variety of imine derivatives have been synthesized via Suzuki cross coupling of *N*-(4-bromophenyl)-1-(3-bromothiophen-2-yl)methanimine with various arylboronic acids in moderate to good yields (58–72%). A wide range of electron donating and withdrawing functional groups were well tolerated in reaction conditions. To explore the structural properties, Density functional theory (DFT) investigations on all synthesized molecules (**3a**–**3i**) were performed. Conceptual DFT reactivity descriptors and molecular electrostatic potential analyses were performed by using B3LYP/6-31G(d,p) method to explore the reactivity and reacting sites of all derivatives (**3a**–**3i**).

**Electronic supplementary material:**

The online version of this article (10.1186/s13065-018-0451-0) contains supplementary material, which is available to authorized users.

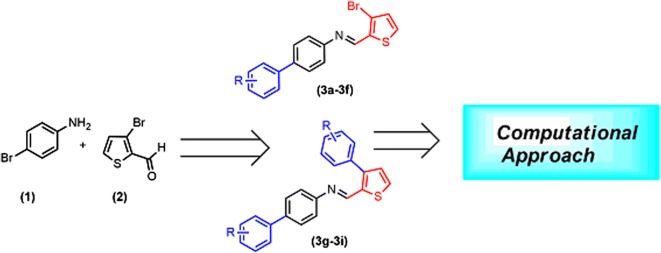

## Background

Imines are an important class of organic compounds and these are synthesized by condensation of primary amines with carbonyl compounds (aldehyde or ketone). They are carrying a (–C=N–) functional group and also known as azomethine [1]. These are pharmaceutically well known for broad spectrum biological activities including antimicrobial [2], analgesic [3], anticonvulsant [4], anticancer [5], antioxidant [6], antihelmintic [7] and many others. Imines are also key component of pigments, dyes, polymer stabilizers, corrosion inhibitors and also used as catalyst and intermediate of various organic reactions [8]. Role of Imines for development of coordination chemistry, inorganic biochemistry is well known [9]. These have been utilized for synthesis of biologically and industrially active compounds via ring closure, replacement and cycloaddition reactions [8]. So, keeping in view the importance of imine functional group we synthesized a novel series of thiophene based imines via Suzuki cross coupling reaction and computational studies of synthesized derivatives was carried to determine their pharmaceutical potential.

## Results and discussion

### Chemistry

In present studies the Suzuki cross coupling of *N*-(4-bromophenyl)-1-(3-bromothiophen-2-yl)methanimine (**3**) with various arylboronic acids has been investigated. According to best of our knowledge no such study about derivatization of imines via Suzuki cross coupling reaction has been reported before.

**Scheme 1:**
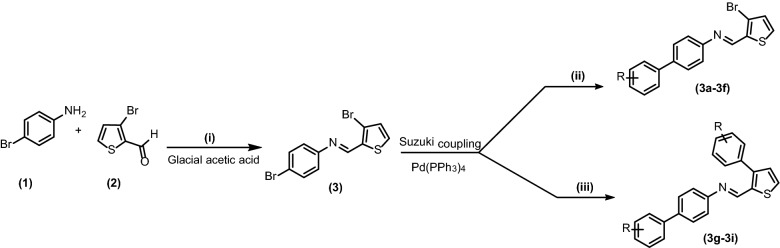
Synthesis of *N*-(4-bromophenyl)-1-(3-bromothiophen-2-yl)methanimine (**3**) and Suzuki coupling of imine with arylboronic acids. Conditions: (i) 1 (1.74 mmol, 0.3 g), 2 (1.74 mmol, 0.33 g), ethanol (10 ml), glacial acetic acid (5–6 drops). (ii) 3 (0.29 mmol, 0.1 g), arylboronic acid (0.32 mmol), K_3_PO_4_ (0.58 mmol,0.12 g), Pd(pph_3_)_4_ (1.45 mmol, 0.01 g), 1,4-dioxane:H_2_O (4:1), reflux 12 h, 95 °C, (iii) 3 (0.29 mmol, 0.1 g), arylboronic acid (0.80 mmol, 0.12 g), K_3_PO_4_ (0.58 mmol, 0.12 g), Pd(pph_3_)_4_ (1.45 mmol, 0.01 g), 1,4-dioxane:H_2_O (4:1), reflux 12 h, 95 °C

In the first step commercially available 4-bromoaniline (**1**) was condensed with 3-bromothiophene-2-carbaldehyde (**2**) in the presence of glacial acetic acid and product *N*-(4-bromophenyl)-1-(3-bromothiophen-2-yl)methanimine (**3**) was obtained in 70% yield. In second step Suzuki coupling of *N*-(4-bromophenyl)-1-(3-bromothiophen-2-yl)methanimine (**3**) with various arylboronic acids was carried out which led to the synthesis of corresponding coupled products containing –C=N–functional group (**3a**–**3f**, **3g**–**3i**) in moderate to good yields 58–72, 67–71% respectively (Scheme 1, Table 1). The results revealed that the compound **3e**, **3h**, **3i** showed good yields 72, 71, 70% respectively, while other compounds **3d**, **3g**, **3b**, **3f**, **3c**, **3a** showed moderate yields (68, 67, 65, 62, 61, 58%) respectively. A wide range of functional groups were well tolerated in reaction conditions.

**Table 1:**
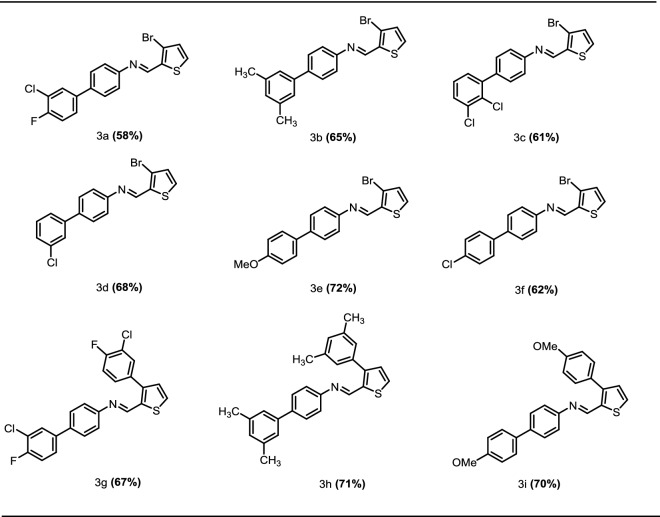
Substrate scope of Suzuki coupling of *N*-(4-bromophenyl)-1-(3-bromothiophen-2-yl)methanimine with arylboronic acids

In additionally, we noted that regio selectivity, when reactions was carried out with 1 eq. boronic acids. Therefore during the transmetallation, bromide moiety of the phenyl ring eliminated rather than bromide mioty present of thiophene part of the substrate, the reason is that no steric hindrance was observed. It is also observed that hydrolysis of imine linkage was not occurred during oxidation, addition, transmetallation, even reductive elimination. While various research groups reported the imine bond cleavage during different Catalytic reaction pathway [10–12]. Herein fortunately, moderate to very good yield of the final products were observed without breaking the imine linkage. So we concluded that imine linkage of this substrate is stable and does not break during catalytic reaction conditions, PH, high temperature and even using the base.

### Density functional theory (DFT) studies

To find the structural properties and reactivity’s of synthesized molecules the DFT studies were computed by using GAUSSIAN 09 software. First of all, molecules (**3a**–**3i**) were optimized by using B3LYP/6-31G(d,p) basis set along with the frequency analysis. After optimization the energy minimized structures were used further for the conceptual DFT reactivity descriptors [13, 14] and molecular electrostatic potential (MEP) analysis on the same basis set.

### Molecular electrostatic potential analysis

**Fig. 1:**
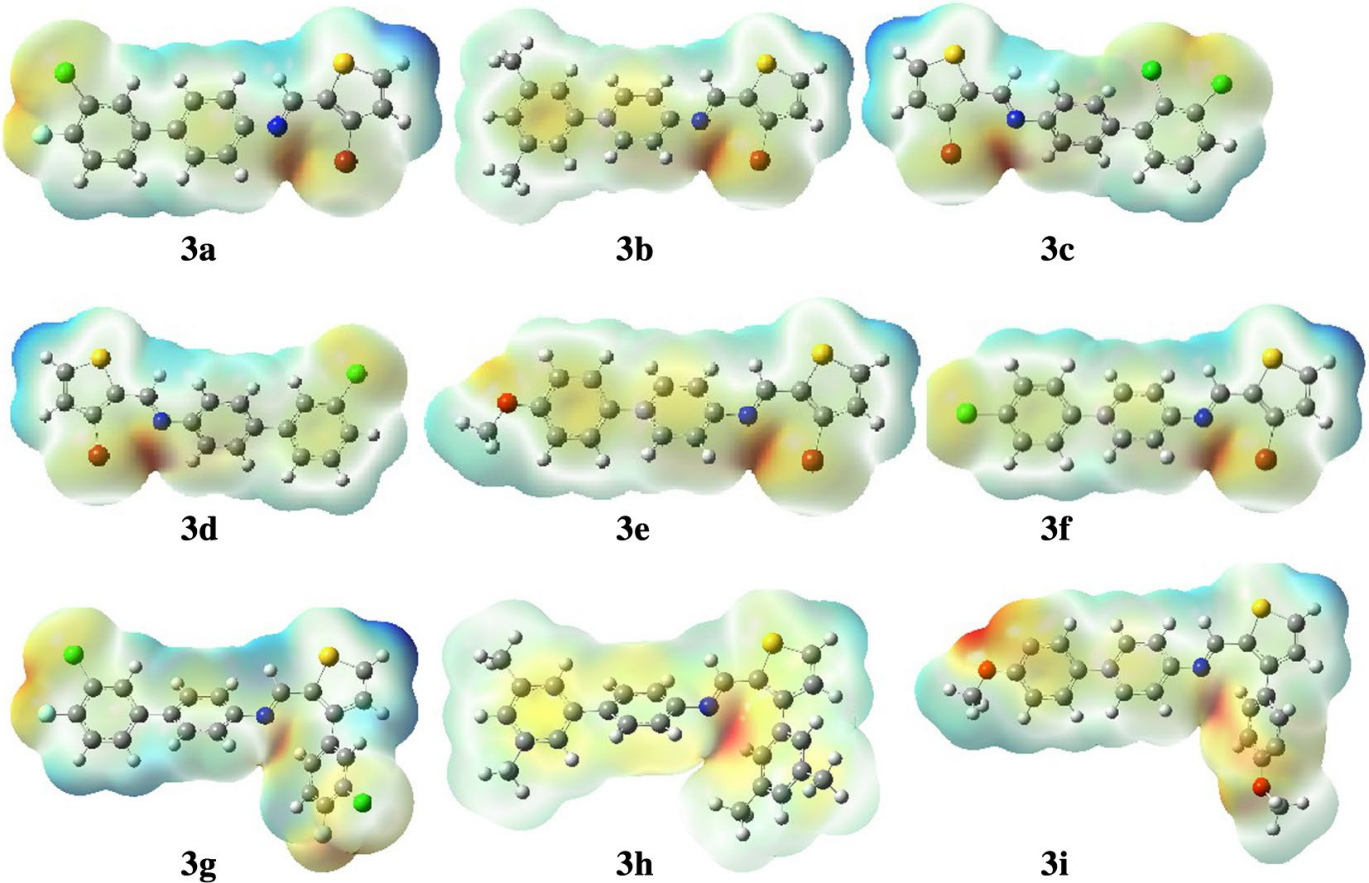
The MEP surfaces of compounds (**3a**–**3i**), red color is indicative of negative potential, whereas blue color is indicative of site of positive potential

Molecular electrostatic potential analysis by using computational methods is famous parameter to describe the distribution of charges and electronic density in newly synthesized compounds [15–17]. MEP analysis of compounds (**3a**–**3i**) was performed by using B3LYP/6-31G(d,p) method. The dispersion of charges is given in the Table 2 and graphics are given in the Fig. 1.

**Table 2:** MEP values of all compounds (**3a**–**3i**)

S. no.	−ve potential (a. u.)	+ve potential (a. u.)
**3a**	− 0.039	0.039
**3b**	− 0.044	0.044
**3c**	− 0.039	0.039
**3d**	− 0.040	0.040
**3e**	− 0.045	0.045
**3f**	− 0.040	0.040
**3g**	− 0.034	0.034
**3h**	− 0.046	0.046
**3i**	− 0.042	0.042

Graphics shown in Fig. 1 reflect that in all derivatives the negative potential is concentrated on the N=CH moiety, which is the attractive site for the positively charged species. On the other hands, the positive potential is located on the protons of thiophene ring in all derivatives (**3a**–**3i**). The dispersion of electronic density of all derivatives is given in the Table 3. The dispersion of charges in **3h** is maximum, which ranges from − 0.046 to 0.046 a. u., whereas in **3g** is minimum that ranges from − 0.034 to 0.034 a. u.

**Table 3:** Ionization potential (I), electron affinity (A), chemical hardness (η), electronic chemical potential (µ), electrophilicity index (ω) nucleophilicity index (N), Fukui function (*f*_*k*_^+^ and *f*_*k*_^*¯*^)

S. no.	I (eV)	A (eV)	*η* (eV)	*µ*(eV)	*ω*(eV)	N (eV)	*f*_*k*_^+^	*f*_*k*_^*−*^
**3a**	− 5.82	− 2.07	1.87	− 3.94	4.15	3.29	0.08	0.21
**3b**	− 5.55	− 1.89	1.83	− 3.72	3.78	3.56	0.11	0.08
**3c**	− 5.90	− 2.03	1.93	− 3.96	4.06	3.21	0.39	0.32
**3d**	− 5.80	− 2.03	1.88	− 3.91	4.06	3.31	0.10	0.08
**3e**	− 5.36	− 1.88	1.74	− 3.62	3.76	3.75	0.11	0.06
**3f**	− 5.75	− 2.04	1.85	− 3.89	4.08	3.36	0.10	0.08
**3g**	− 5.80	− 2.18	1.81	− 3.99	4.39	3.31	0.10	0.07
**3h**	− 5.38	− 1.77	1.80	− 3.57	3.54	3.73	0.10	0.07
**3i**	− 4.52	− 4.17	0.17	− 4.34	55.39	4.59	0.16	0.15

### Conceptual DFT reactivity descriptors

The conceptual DFT reactivity descriptors such as ionization potential (I), electron affinity (A), chemical hardness (η), electronic chemical potential (µ), electrophilicity index (ω) [14] nucleophilicity index (N) [18] Fukui functions (*f*_*k*_^+^ and *f*_*k*_^*¯*^*)* as well as Parr functions [19, 20] are very helpful for the explanation of the reactivity of any molecule. The values of all important reactivity descriptors of all compounds are given in the Table 3. As per accordance with Koopmans’ theorem of closed-shell compounds, the energy values of the highest occupied molecular orbital (E_HOMO_) and the lowest unoccupied molecular orbital (E_LUMO_) correspond to the ionization potential (I) and electron affinity (A), respectively [15]. With the help of these values chemical hardness (η), electronic chemical potential (µ), electrophilicity index (ω) and can be determined easily.

The chemical hardness of any compound can be expressed in term of the following equation [21]:

$$\eta = \, \left( {{\text{E}}_{\text{HOMO}} - {\text{E}}_{\text{LUMO}} } \right)/2$$

The chemical hardness of all compounds is found in the range of 0.17–1.93 eV (Table 3). From values it is cleared that the compound **3d** has highest value (1.93 eV) and chemically less reactive. Whereas **3i** has lowest value i.e. of 0.17 eV and most reactive among all derivatives. The Electronic chemical potential (*µ*) of any compound express the charge transfer within compound in ground state and mathematically can be defined as follow by equation.

$$\eta = \left( {{\text{E}}_{\text{HOMO}} + {\text{E}}_{\text{LUMO}} } \right)/2$$

The electronic chemical potential values of all compounds (**3a**–**3i**) are found in the range of − 3.57 to − 4.34 eV. The compound **3h** has highest value, whereas **3i** has lowest value among all. Like chemical hardness and chemical potential, the concept of electrophilicity index (ω) was given by Parr et al. [22]. This reactivity index calculates the stabilization in energy when the system gets an additional charge from the outer environment. Mathematically, the electrophilicity index is defined by the following equation [23]:

$$\omega = \mu^{2} /2\eta$$

Among all the synthesized compounds, the **3i** has highest value of electrophilicity index i.e. 55.39 eV. This exceptionally very high value indicates that **3i** has very strong potential to accept the charge from the outer source. This is due to reason because it had doner:acceptor:doner (D:A:D) electronic groups attached through conjugation in its skeleton [24]. The nucleophilicity (Ν) index [25] is another very important reactivity descriptor for describing the reactivity of organic compounds. We calculated the nucleophilicity by using the following mathematical expression:

$${\text{N}}\left( {\text{Nu}} \right) \, = {\text{ E}}_{{{\text{HOMO}}\, ( {\text{Nu)}}}} \left( {\text{eV}} \right) - {\text{E}}_{{{\text{HOMO}}\, ( {\text{TCE)}}}} \left( {\text{eV}} \right)$$

Tetracyanoethylene (TCE) is used as a reference standard because it has the lowest HOMO energy in a large series of organic molecules which are considered already. The nucleophilicity index of all synthesized compounds (**3a**–**3i**) is found in the range of 3.21–4.59 eV. Among all the lowest value of N is for **3c**, i.e. of 3.21 eV, which is classified as soft nucleophile and highest value is **3i**, i.e. of 4.59 eV (strongest nucleophile among all).

In the last few years, the Fukui functions are extensively used to identify the local reactivity (electrophilic or nucleophilic) sites of compounds [26]. N + 1 and N – 1 calculations were carried out for an N electrons system by single point energy calculations and B3LYP/6-31G(d,p) method. The electronic population for an atom k in the molecules was calculated from NBO analysis. The mathematical equations of condensed form of Fukui functions for an atom k in a compound for nucleophilic, electrophilic attacks are:

$$f_{\text{k}}^{ + } = \, \left[ {{\text{q}}_{\text{k}} \left( {N + 1} \right) - {\text{q}}_{\text{k}} \left( N \right)} \right]{\text{ for nucleophilic attack}}$$

$$f_{\text{k}}^{ - } = \, \left[ {{\text{q}}_{\text{k}} \left( N \right) - {\text{q}}_{\text{k}} \left( N \right) - 1} \right]{\text{ for electrophilic attack}}$$

where qk is the electronic population of atom k of compound.

The highest values of *f*_k_^+^ and *f*_k_^¯^ of all compounds are given in the Table 3. The Fukui functions results are in total agreement with the ESP results. In all compounds almost the all the hetro atoms (N and S) sites are favorable for the electrophilic attack (for detailed values see Table 3).

**Fig. 2:**
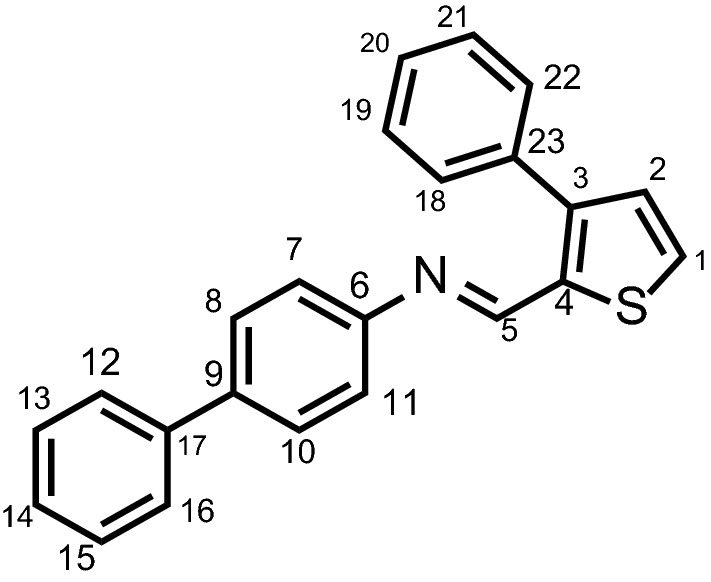
Labelling scheme for discussion of Parr functions

In order to look further look insight of the reactivity of the all compounds, we also investigated the electrophilic (*P*_*k*_^+^) and nucleophilic (*P*_*k*_^−^) Parr functions by calculation the single point energy calculations under radical cationic and anionic conditions [23]. Once the values of *P*_*k*_^+^ and *P*_*k*_^−^ were calculated, we also calculated the local electrophilicity and local nucleophilicity of all compounds with the help of following equations [27].

$$\omega_{k} = \, \omega P_{k}^{ + } \left( {\text{local electrophilicity}} \right)$$

$$N_{k} = \, N \, P_{k}^{ - } \left( {\text{local nucleophilicity}} \right)$$

where the ω and N are electrophilicity index and band gap of frontier orbitals, respectively. The detailed values of Parr functions and local electrophilicity as well as nucleophilicity of all compounds are provided in the Table 4. From the values provided in the Table it is clear that most electrophilic center is C5, which is directly attached to the –N = moiety (see Fig. 2 for labelling) in compound **3a**–**3h**. In **3i** the trend is different, and the most electrophilic carbon is C15, which is next to the methoxy substituent. The *P*_*k*_^−^ value reflects that the most nucleophilic center in **3a**, **3c**–**3h** is C9 of biphenyl core and in **3b** is C14, in **3i** is C15. In **3i** the electrophilic and nucleophilic centers are concentrated on the similar carbon, the reason of this exceptional behavior is not clear. The local electrophilicity results shows that among all the **3i** is most electrophilic in nature having very high value of 35.56. The local nucleophilicity analysis reflects that **3c** is most nucleophilic and **3i** is least nucleophilic among all synthesized compounds.

**Table 4:** Electrophilic (*P*_*k*_^+^) and nucleophilic (*P*_*k*_^−^) Parr functions, local electrophilicity (*ω*_*k*_) and local nucleophilicity (*N*_*k*_) of all compounds (**3a**–**3i**)

Compounds	*P*_*k*_^+^	*P*_*k*_^−^	*ω*_*k*_	*N*_*k*_
**3a**	0.22 (C5)	0.17 (C9)	0.91	0.67
**3b**	0.21 (C5)	0.18 (C14)	0.81	0.67
**3c**	0.21 (C5)	0.21 (C9)	0.86	0.84
**3d**	0.21 (C5)	0.19 (C9)	0.88	0.72
**3e**	0.21 (C5)	0.12 (C9)	0.82	0.44
**3f**	0.21 (C5)	0.17 (C9)	0.88	0.63
**3g**	0.20 (C5)	0.15 (C9)	0.91	0.56
**3h**	0.21 (C5)	0.15 (C9)	0.74	0.55
**3i**	0.64 (C15)	0.98 (C15)	35.56	0.34

### Frontier molecular orbitals analyses by using FERMO concept

FERMO concept is recently introduced in the literature where frontier orbitals other than HOMO and LUMO are taken into account to explain the reactivities of compounds under consideration [28–30]. In the FERMO concept, adequate orbital shape and composition are correlated with the reactivity indexes. It has been realized that a frontier molecular orbital other than HOMO and LUMO may have large contribution on atoms present at the active site. These frontier orbitals can fit the orbital choice criterion because they are present in all compounds under study and better correlate with the experimental observation rather than HOMO and LUMO.

**Fig. 3:**
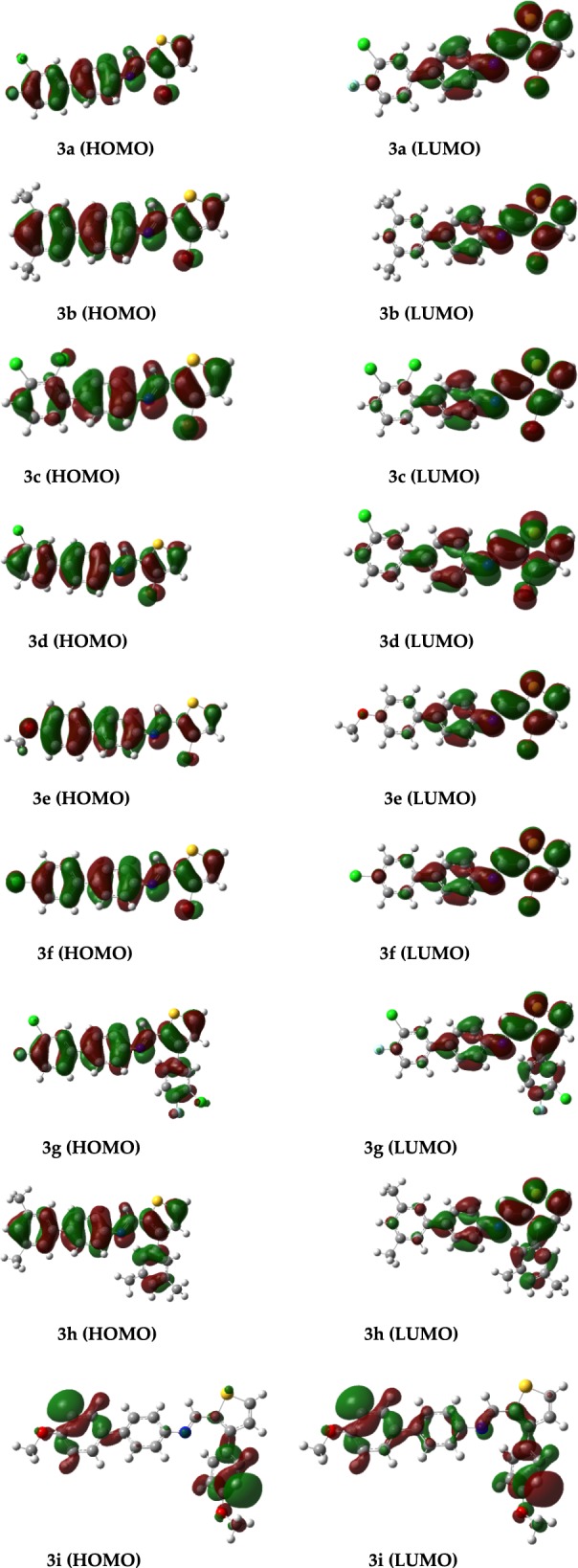
HOMO–LUMO surfaces showing the isodensities of all compounds (**3a**–**3i**)

**Fig. 4:**
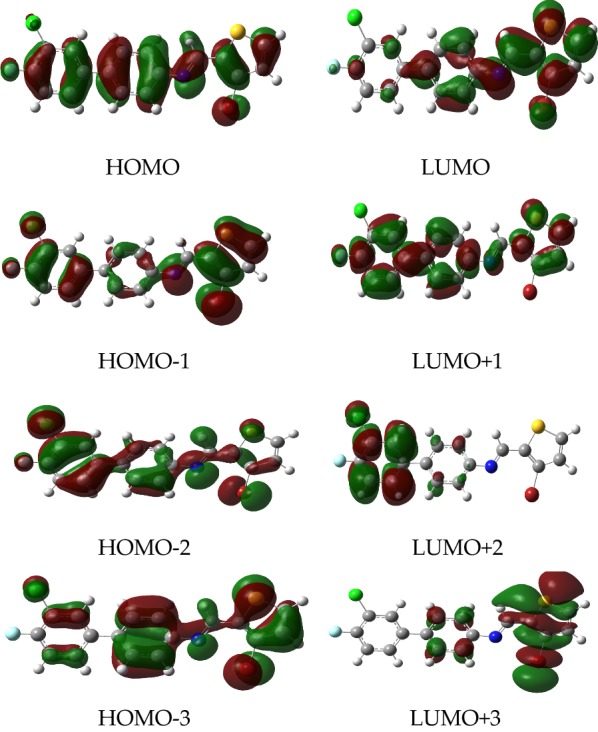
HOMO, HOMO−1, HOMO−2, HOMO−3 and LUMO, LUMO+1, LUMO+2, LUMO+3 surfaces of **3a**

In this study, we have correlated the calculated electrophilicities nucleophilicities with the FERMO concept. The P_k_^+^ of compound shows that the atom 5 has the highest reactivity whereas C9 has the highest reactivity for P_k_^−^. A number of frontier orbital ranging from HOMO−3 to LUMO+3 are analyzed to see which molecular orbital has contribution from atom 5 atom 9. The analysis reveals that HOMO and LUMO are the appropriate orbitals with maximum contributions from atoms present in the active sites (APAS). Similarly, we have analyzed frontier molecular orbitals (HOMO−3 to LUMO+3) for all compounds and are given in the supporting information (Additional file 1: Figure S1). The results reveal that in all of these cases, the HOMO and LUMO have maximum contribution from atom involved in the active sites. The HOMO and LUMO of all compounds are shown in Figs. 3 and 4 where it can be easily rationalized why compound **3b** and **3i** behave differently than all other compounds. For all other compounds atoms 5 and 9 have the highest contribution to justify the highest P_k_^+^ and P_k_^−^. For compound **3i**, the orbital densities are present on atom 15 in HOMO as well as in LUMO which is consistent with its P_k_^−^ and P_k_^+^. Therefore, it can be concluded that the HOMO and LUMO are the FERMO for nucleophilicities and electrophilicities.

## Materials and methods

### General information

Melting points were determined with help of (Buchi B-540) melting point apparatus (Buchi, New Castle, DE, USA). Proton (1H) NMR and Carbon (13C) NMR spectra were obtained in CDCl_3_ at 500/126 MHz (Bruker, Billercia, MA, USA), respectively. EI-MS spectra were obtained on JMS-HX-110 spectrometer (JEOL, Peabody, MA, USA). Silica gel (70–230 mesh) was used for purification of compounds in column chromatography. The reactions were monitored on TLC, using Merck silica gel 60 PF_254_ cards. Visualization of compounds was done by using UV lamp (254–365 nm).

### General procedure for synthesis of Schiff base *N*-(4-bromophenyl)-1-(3-bromothiophen-2-yl)methanimine

First of all round bottom flask took and dried in an oven. 4-bromoaniline in ethanolic solution was condensed with 3-bromothiophene-2-carbaldehyde in the presence of few drops of glacial acetic acid. Then the mixture was refluxed for 6–10 h on water bath. After 6–10 h yellow coloured Schiff base was filtered, washed and purified by column chromatography [31].

### General procedure for Suzuki coupling of Schiff base with arylboronic acids

The palladium catalyst Pd(PPh_3_)_4_ was added in *N*-(4-bromophenyl)-1-(3-bromothiophen-2-yl)methanimine (**3**), under nitrogen gas. The 1,4-dioxane was used as solvent and reaction mixture stirred for 30 min. After that arylboronic acid, K_3_PO_4_ and water were added [32, 33] and mixture was stirred for 12 h at 90 °C. After cooling to normal temperature, the mixture was diluted with ethyl acetate. After separation the organic layer was dried with MgSO_4_ and the solvent was removed under vacuum. The purification of crude residue was done by column chromatography by using ethyl-acetate and *n*-hexane, and further characterization was done by using different spectroscopic techniques.

### Characterization data

#### (*E*)-*N*-(4-bromophenyl)-1-(3-bromothiophen-2-yl)methanimine (**3**)

Obtained as solid, mp = 114 °C, ^1^H NMR (500 MHz, CDCl_3_): δ 8.65 (s, 1H), 7.48 (d, *J* = 7.0, 2H), 7.15 (d, *J* = 6.8 Hz, 2H), 7.35 (d, *J* = 6.5 Hz, 1H), 6.75 (d, *J* = 5.8 Hz, 1H); ^13^C NMR (126 MHz, CDCl_3_): δ 150.1, 145.2, 132.9, 130.1, 125.7, 124.9, 124.5, 123.4, 122.1, 120.1, 109.1. EI/MS *m/z* (%): 346.0 [M+H]^+^; 347 [M+2]; 349 [M+4]; [M-Br] = 263.0, [M-2Br] = 186.1.

#### (*E*)-1-(3-bromothiophen-2-yl)-*N*-(3′-chloro-4′-fluoro-[1,1′-biphenyl]-4-yl)methanimine (**3a**)

Obtained as solid, mp = 125 °C, ^1^H NMR (500 MHz, CDCl_3_): δ 9.75 (s, 1H), 7.78 (dd, *J* = 5.0, 1.5 Hz, 1H), 7.55(dd, *J* = 7.0, 2.5 Hz, 2H), 7.38–7.35 (m, 3H), 7.29–7.26 (m, 2H), 7.21 (d, *J* = 5.0 Hz, 1H); ^13^C NMR (126 MHz, CDCl_3_): δ 148.5, 138.9, 134.5, 132.2, 131.5, 131.1, 131.0, 130.4, 129.4, 129.3, 122.7, 121.9, 121.6, 117.1, 116.9, 116.4, 110.5. EI/MS *m/z* (%): 393.0 [M+H]^+^; 394.5 [M+2];396.5 [M+4]; [M-Br] = 314.0; [M-Cl, F] = 260.4.

#### (*E*)-1-(3-bromothiophen-2-yl)-*N*-(3′,5′-dimethyl-[1,1′-biphenyl]-4-yl)methanimine (**3b**)

Obtained as solid, mp = 131 °C, ^1^H NMR (500 MHz, CDCl_3_): δ 9.91 (s, 1H), 8.52 (d, *J* = 5.0 Hz, 1H), 7.73 (d, *J* = 2.0 Hz, 2H), 7.52–7.46 (m, 3H), 7.28–7.04 (m, 3H), 2.41 (s, 6H); ^13^C NMR (126 MHz, CDCl_3_): δ 153.7, 145.1, 142.1, 138.4, 137.6, 133.9, 132.1, 131.1, 130.6, 130.1, 129.0, 128.0, 127.4, 126.5, 122.8, 121.7, 120.1, 21.9, 21.5. EI/MS *m/z* (%): 371.0 [M+H]^+^; 372.1[M+2]; [M-Br] = 290.0; [M-2CH_3_] = 339.0.

#### (*E*)-1-(3-bromothiophen-2-yl)-*N*-(2′,3′-dichloro-[1,1′-biphenyl]-4-yl)methanimine (**3c**)

Obtained as solid, mp = 128 °C, ^1^H NMR (500 MHz, CDCl_3_): δ 8.62 (s, 1H), 7.80–7.79 (m, 3H), 7.60–7.58 (m, 3H), 7.52–7.50 (m, 2H), 6.57 (d, *J* = 9.0 Hz, 1H), ^13^C NMR (126 MHz, CDCl_3_): δ 152.9, 146.4, 141.8, 137.8, 133.0, 132.0, 130.9, 130.1, 129.0, 128.9, 128.0, 127.5, 127.1, 124.8, 122.8, 122.1, 114.4. EI/MS *m/z* (%): 409.0 [M+H]^+^; 410.1[M+2]; 412.1 [M+4]; 414.1 [M+6], [M-2Cl] = 337.9.

#### (*E*)-1-(3-bromothiophen-2-yl)-*N*-(3′-chloro-[1,1′-biphenyl]-4-yl)methanimine (**3d**)

Obtained as solid, mp = 135 °C, ^1^H NMR (500 MHz, CDCl_3_): δ 8.82 (s, 1H), 7.96 (d, *J* = 3.0 Hz, 2H), 7.48 (d, *J* = 7.0 Hz, 2H), 7.35 (m, 4H), 6.90 (m, 2H), ^13^C NMR (126 MHz, CDCl_3_): δ 150.1, 146.7, 141.2, 140.1, 134.9, 130.1, 129.9, 129.3, 127.8, 126.9, 125.6, 123.9, 123.4, 122, 121.1, 120.2, 112.1. EI/MS *m/z* (%): 377.0 [M+H]^+^; 378.1 [M+2]; 380.4 [M+4], [M-Cl] = 339.9, [M-aryl, Cl fragments] = 264.0.

#### (*E*)-1-(3-bromothiophen-2-yl)-*N*-(4′-methoxy-[1,1′-biphenyl]-4-yl)methanimine (**3e**)

Obtained as solid, mp = 142 °C, ^1^H NMR (500 MHz, CDCl_3_): δ 8.72 (s, 1H), 7.86 (m, 4H), 7.44 (d, *J* = 6.98 Hz, 2H), 7.00 (m, 2H), 6.97 (m, 2H), 3.65 (s, 3H). ^13^C NMR (126 MHz, CDCl_3_): δ 160.2, 154.1, 148.2, 140.1, 134.1, 132.1, 131.3, 130.2, 129.1, 125.1, 124.1, 123.1, 122.1, 120.1, 115.6, 113.1, 109.1, 56.1. EI/MS *m/z* (%): 373.0 [M+H]^+^; 374.1 [M+2], [M-OMe] = 340.1 [M-Br, OMe] = 261.1.

#### (*E*)-1-(3-bromothiophen-2-yl)-*N*-(4′-chloro-[1,1′-biphenyl]-4-yl)methanimine (**3f**)

Obtained as solid, mp = 128 °C, ^1^H NMR (500 MHz, CDCl_3_): δ 8.72 (s, 1H), 7.90 (d, *J* = 5.0 Hz, 2H), 7.82 (d, *J* = 7.0 Hz, 2H), 7.31 (m, 4H), 6.90 (m, 2H), ^13^C NMR (126 MHz, CDCl_3_): δ 151.1, 146.2, 140.2, 139.1, 137.9, 132.1, 129.9, 129.2, 128.1, 127.0, 124.6, 123.5, 123.1, 122.0, 121.1, 120.1, 111.1. EI/MS *m/z* (%): 377.0 [M+H]^+^; 378.1 [M+2]; 380.4 [M+4], [M-Cl] = 339.9.

#### (*E*)-*N*-(3′-chloro-4′-fluoro-[1,1′-biphenyl]-4-yl)-1-(3-(3-chlor*o*-4-fluorophenyl)thiophen-2-yl)methanimine (**3g**)

Obtained as solid, mp = 125 °C, ^1^H NMR (500 MHz, CDCl_3_): δ 8.61 (s, 1H), 7.92 (m, 6H), 7.61 (d, *J *= 6.58 Hz, 2H), 7.75 (d, *J *= 7.25 Hz, 2H), 7.20 (m, 2H), ^13^C NMR (126 MHz, CDCl_3_): δ 160.1, 156.7, 150.1, 145.1, 139.0, 137.9, 136.8, 134.5, 131.9, 130.8, 130.1, 129.9, 129.1, 128.3, 127.1, 123.8, 122.9, 122.1, 121.1, 120.1, 119.7, 118.1, 116.1. EI/MS *m/z* (%): 445.4 [M+H]^+^; 446.1 [M+2]; 448.1 [M+4]; [M-2Cl] = 375.0; [M-2Cl, F] = 357.4.

#### (*E*)-*N*-(3′,5′-dimethyl-[1,1′-biphenyl]-4-yl)-1-(3-(3,5-dimethylphenyl)thiophen-2-yl)methanimine (3h)

Obtained as solid, mp = 127 °C, ^1^H NMR (500 MHz, CDCl_3_): δ 8.51 (s, 1H), 7.82 (m, 4H), 7.61 (d, *J *= 5.58 Hz, 2H), 7.52 (d, *J *= 8.0 Hz, 2H), 7.10 (m, 4H), 2.50 (s, 12H). ^13^C NMR (126 MHz, CDCl_3_): δ 153.1, 148.1, 141.1, 1401.1, 139.8, 139.1, 138.1, 137.1, 136.1, 135.1, 131.1, 130.9. 130.1, 129.9, 129.1, 128.4, 128.1, 127.1, 126.8, 126.1, 125.1, 121.4, 120.1, 21.8, 21.0, 20.1, 19.8. EI/MS *m/z* (%): 396.1 [M+H]^+^; [M-CH_3_] = 382.0; [M-4CH_3_] = 338.1.

#### (*E*)-*N*-(4′-methoxy-[1,1′-biphenyl]-4-yl)-1-(3-(4-methoxyphenyl)thiophen-2-yl)methanimine (**3i**)

Obtained as solid, mp = 140 °C, ^1^H NMR (500 MHz, CDCl_3_): δ 8.72 (s, 1H), 7.71 (m, 6H), 7.61 (m 2H), 7.52 (m, 2H), 7.10 (m, 4H), 3.50 (s, 6H). ^13^C NMR (126 MHz, CDCl_3_): δ 160.1, 158.1, 152.5, 147.1, 139.1, 136.1, 131.1, 133.2, 130.9, 130.2, 129.9, 129.2, 128.2, 127.9, 127.0, 122.9, 122.1, 121.4, 120.9, 114.9, 114.2, 113.1, 112.1, 55.8, 55.0. EI/MS *m/z* (%): 400.3 [M+H]^+^; [M-CH_3_] = 3368.0 [M-2CH_3_] = 338.0.

### Computational methods

Calculations were performed with the help of GAUSSIAN 09 software [34], visualization of results and graphics were executed by using GaussView 05 program [35]. The geometries of all compounds (**3a**–**3i**) were optimized at B3LYP/6-31G(d,p) level of DFT and confirmed with the help of vibrational analysis (no single imaginary frequency). The optimized geometries further used for conceptual DFT reactivity descriptors including the Fukui as well as Parr functions and molecular electrostatic potential (MEP) analyses at the same level of theory.

## Conclusions

In present study we have synthesized a variety of thiophene based imine derivatives (**3a**–**3i**) via Palladium catalyzed Suzuki reaction in moderate to good yields (58–72%). Both electron donating and withdrawing groups were well tolerated in reaction conditions. DFT studies reflect that all molecules (**3a**–**3i**) are relatively less stable and more reactive. The reactivity descriptors revealed that **3i** is most reactive among all the synthesized derivatives. The MEP analysis reelects that negative potential lies on the N=CH moiety in all derivatives (**3a**–**3i**). The local electrophilicity results shows that among all the **3i** is most electrophilic in whereas **3c** is most nucleophilic among all synthesized compounds. In light of this research, synthesized Imine derivatives might be a potential source of therapeutic agents. Future investigations in this dimension will provide new visions towards development of novel pharmaceutically important drugs.

## Additional file

**Additional file 1: Figure S1.** HOMO, HOMO−1, HOMO−2, HOMO−3 and LUMO, LUMO+1, LUMO+2, LUMO+3 surfaces of **3b**–**3i.**
