# Supplementary material for: Facile synthesis of N- (4-bromophenyl)-1- (3-bromothiophen-2-yl)methanimine derivatives via Suzuki cross-coupling reaction: their characterization and DFT studies
Source: Chem Cent J. 2018 Jul 17;12:84. doi: 10.1186/s13065-018-0451-0 (PMC6049850; doi:10.1186/s13065-018-0451-0)
Supplement: Supplementary file 1 — Additional file 1: Figure S1. HOMO, HOMO−1, HOMO−2, HOMO−3 and LUMO, LUMO+1, LUMO+2, LUMO+3 surfaces of 3b–3i. [file 13065_2018_451_MOESM1_ESM.docx]

**Additional file**

| **3b** | |
| --- | --- |
| 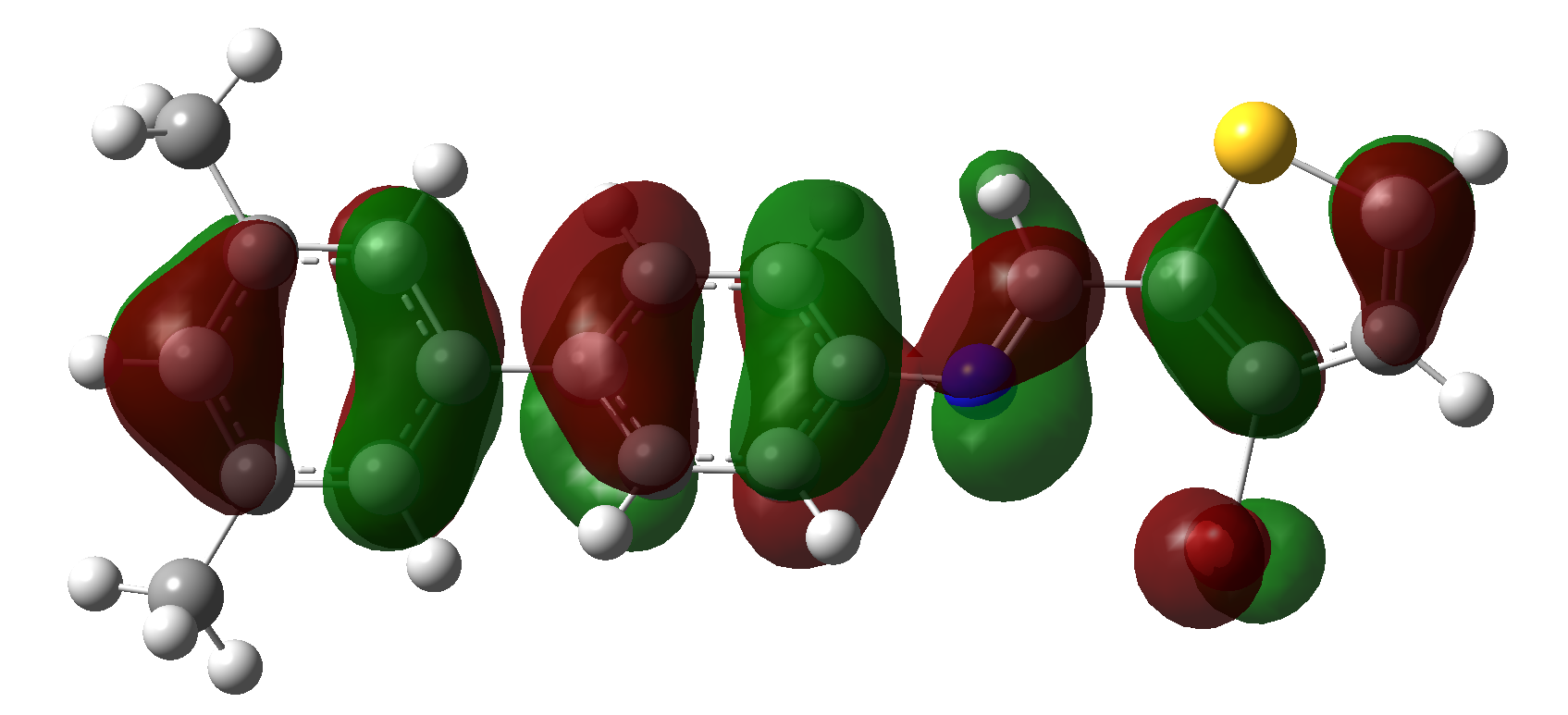  HOMO | 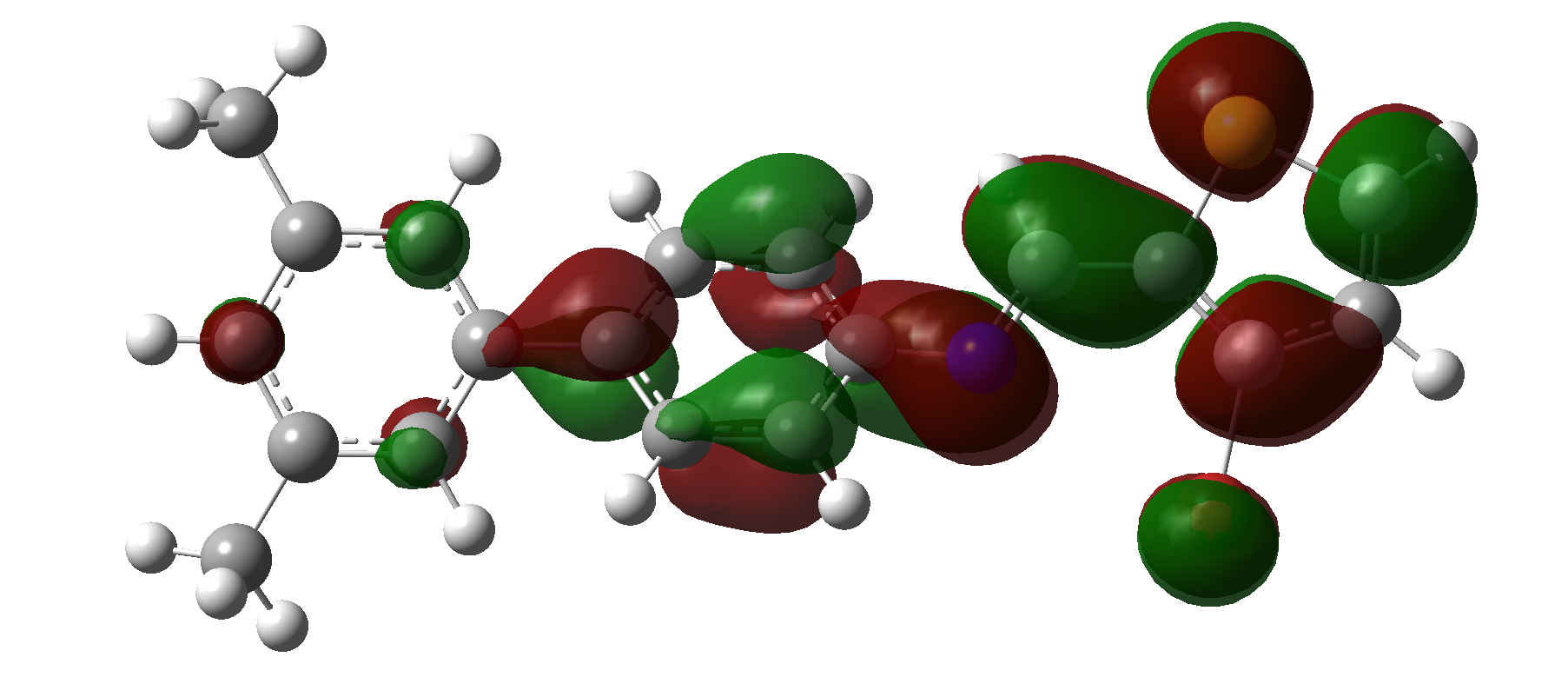  LUMO |
| 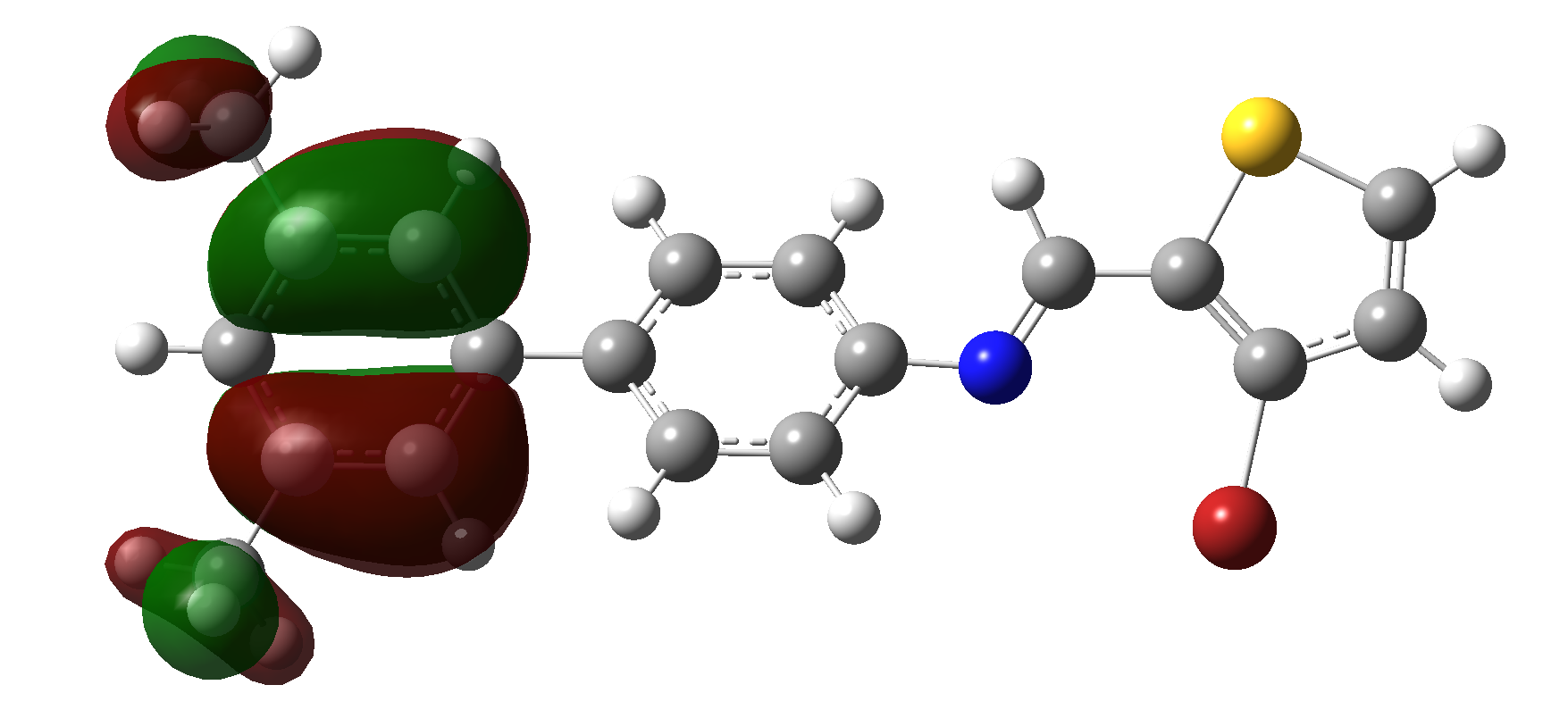  HOMO-1 | 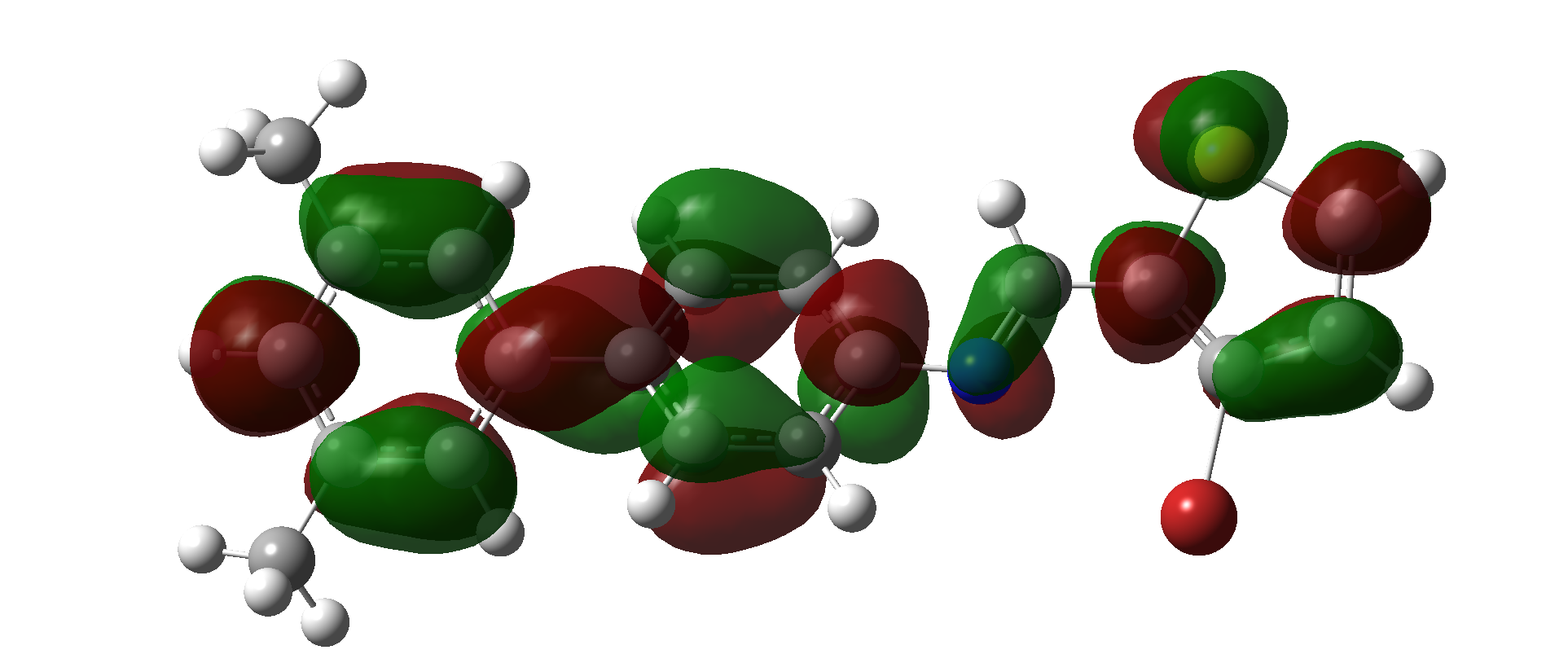  LUMO+1 |
| 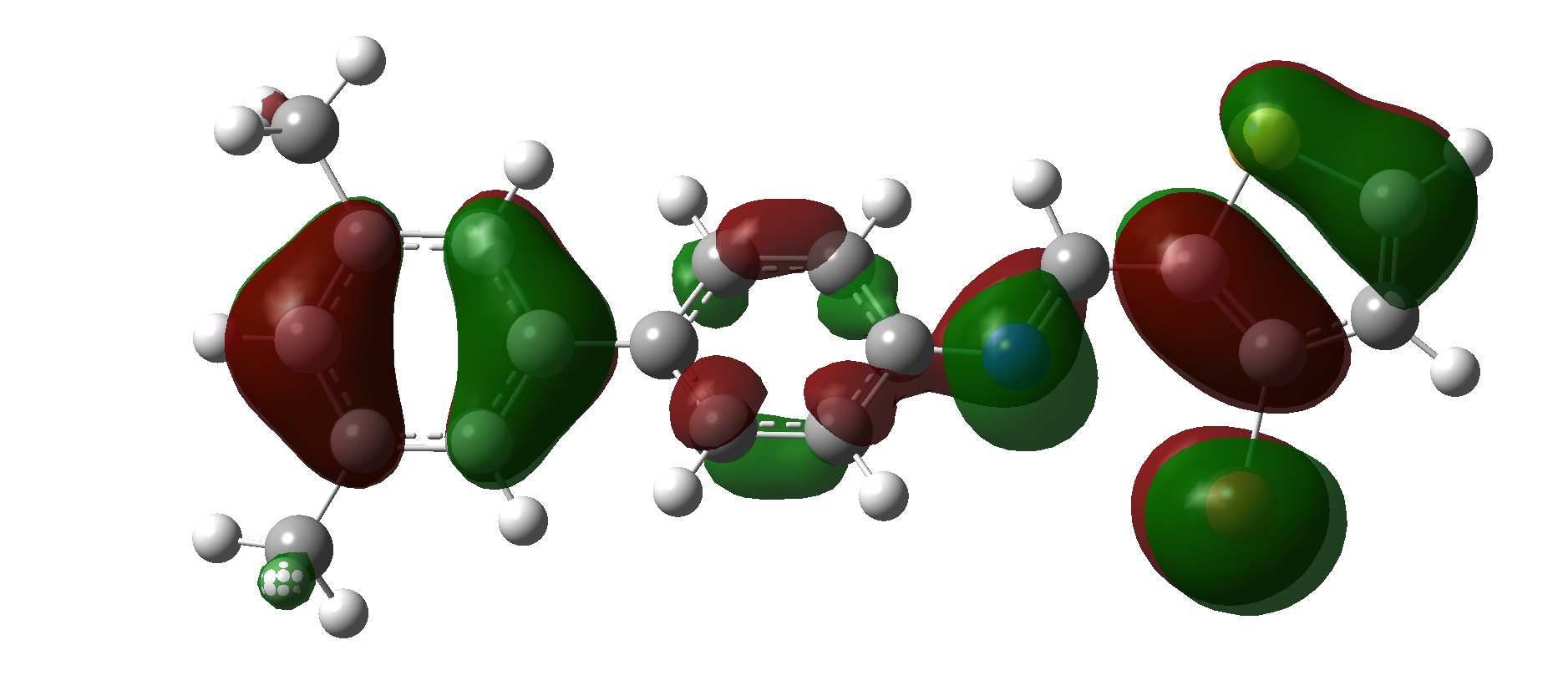  HOMO-2 | 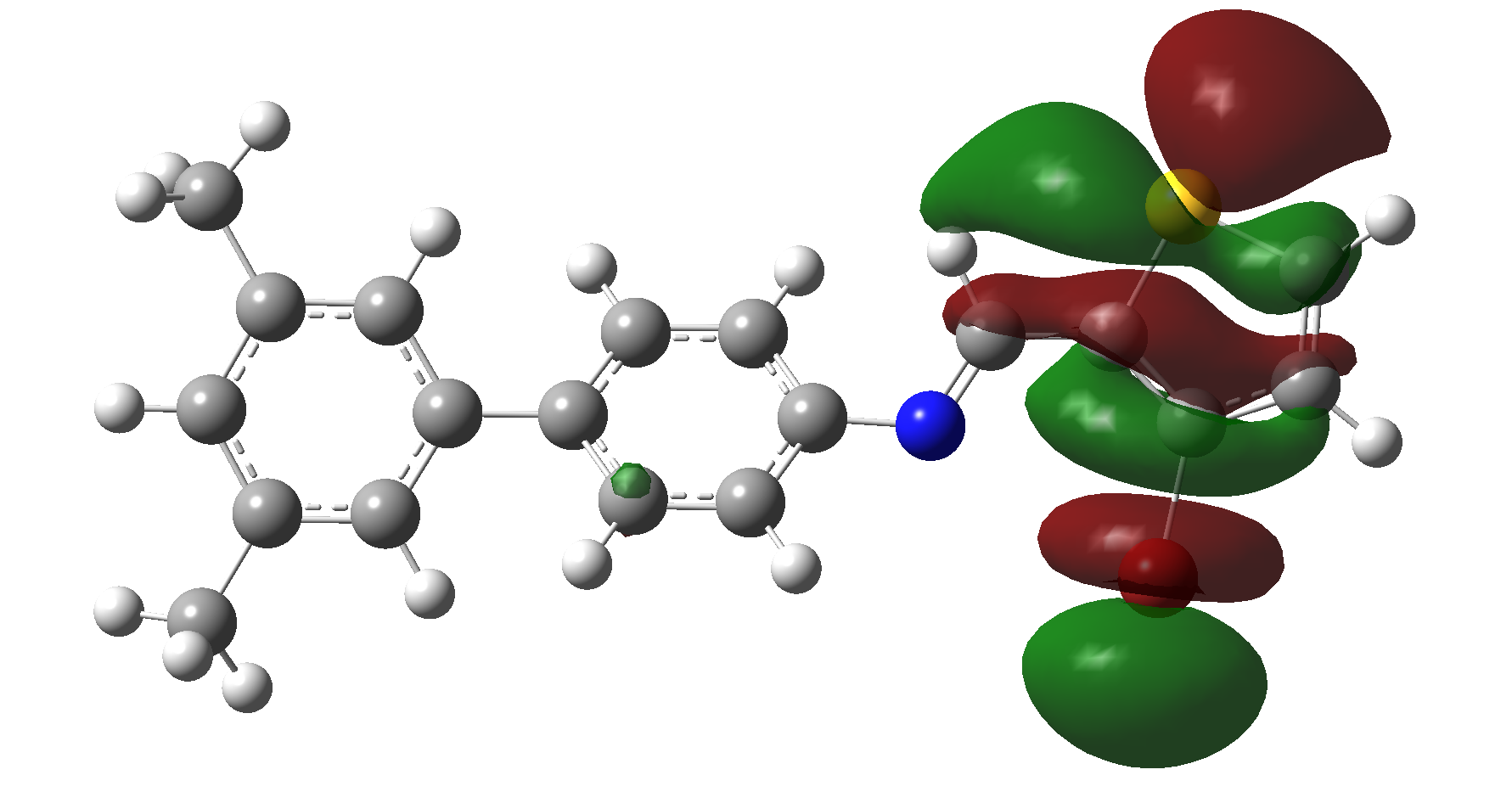  LUMO+2 |
| 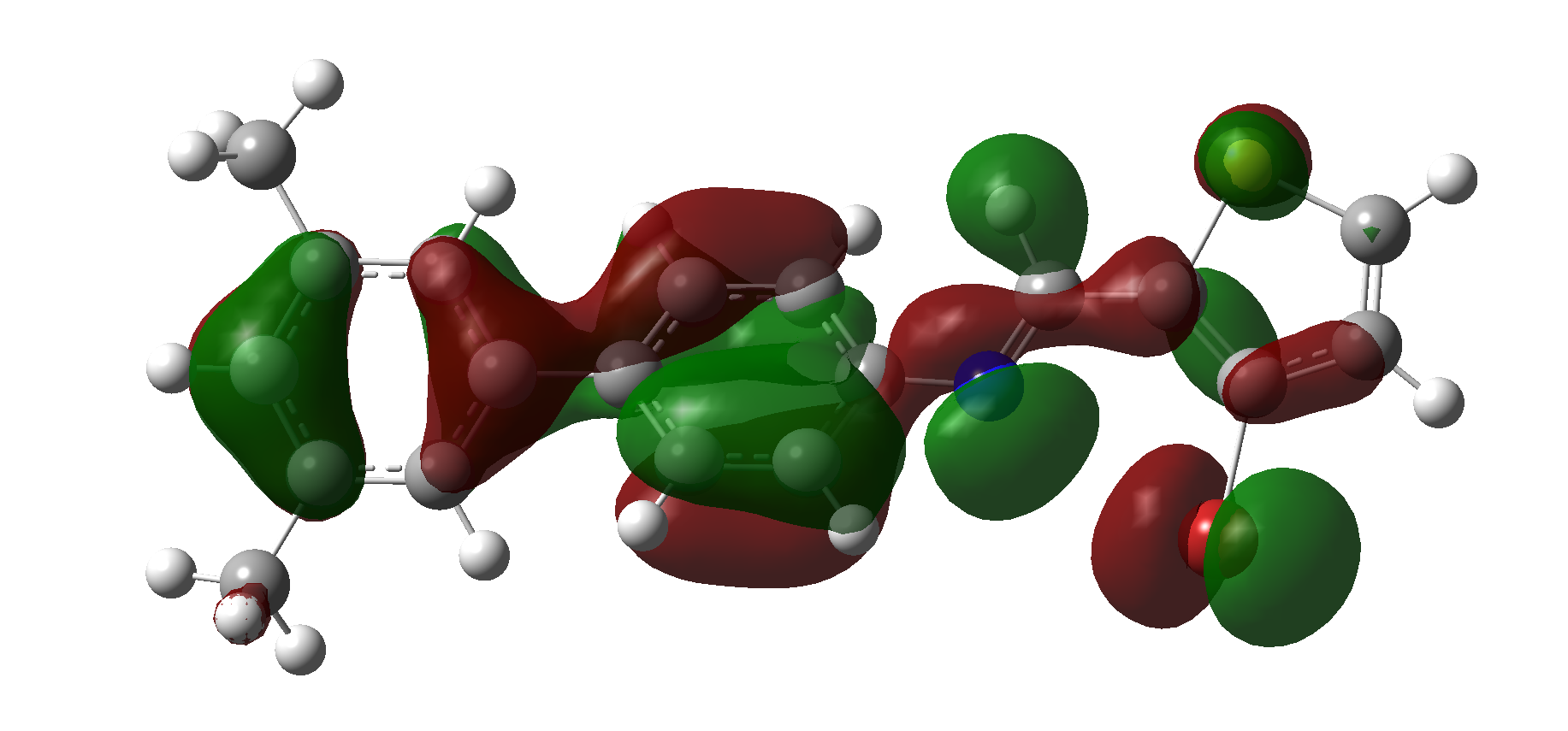  HOMO-3 | 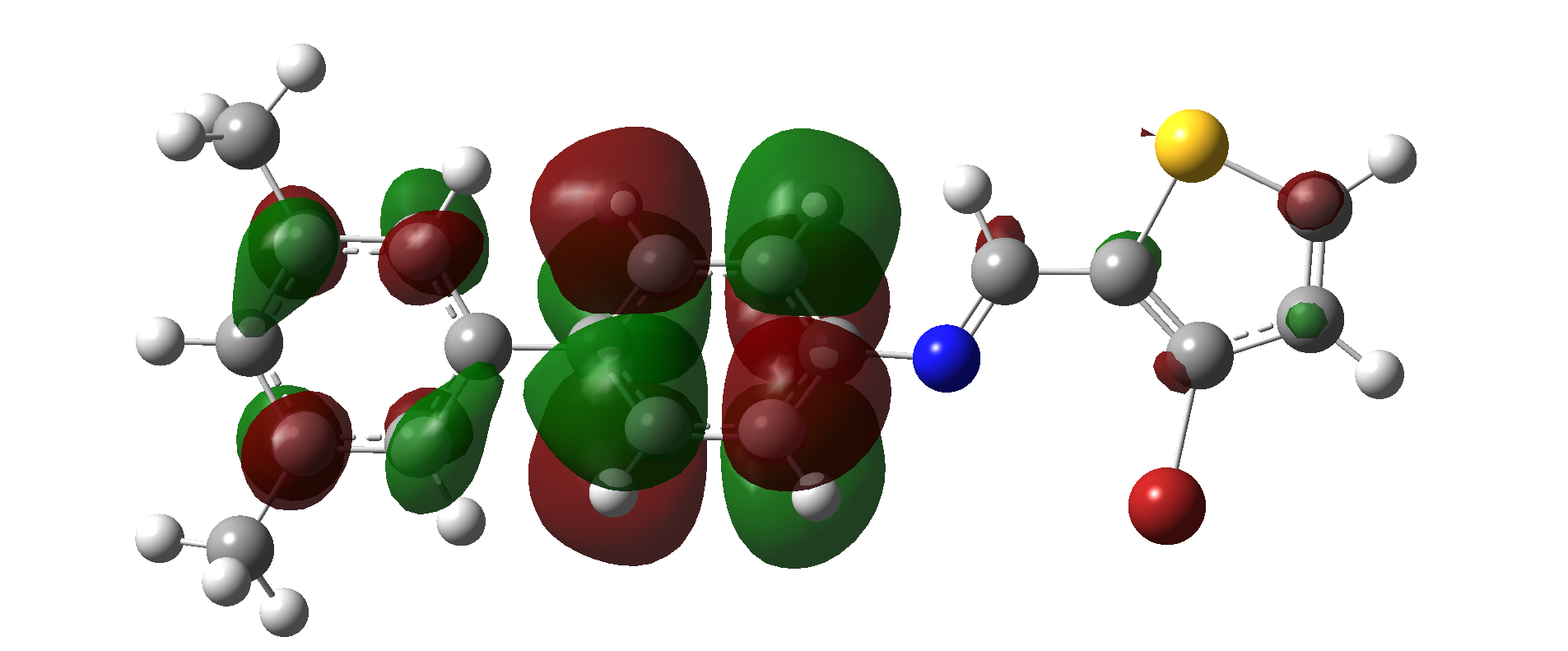  LUMO+3 |

| **3c** | |
| --- | --- |
| 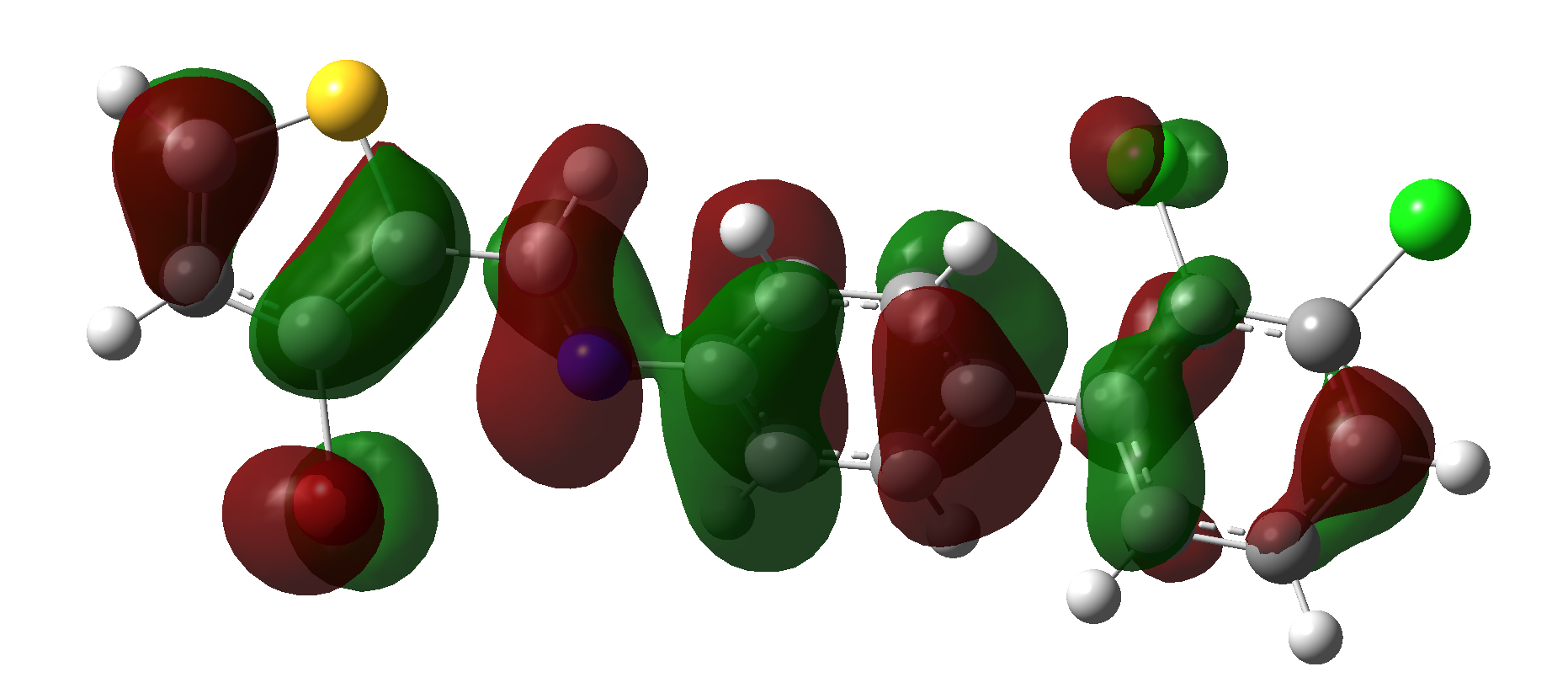  HOMO | 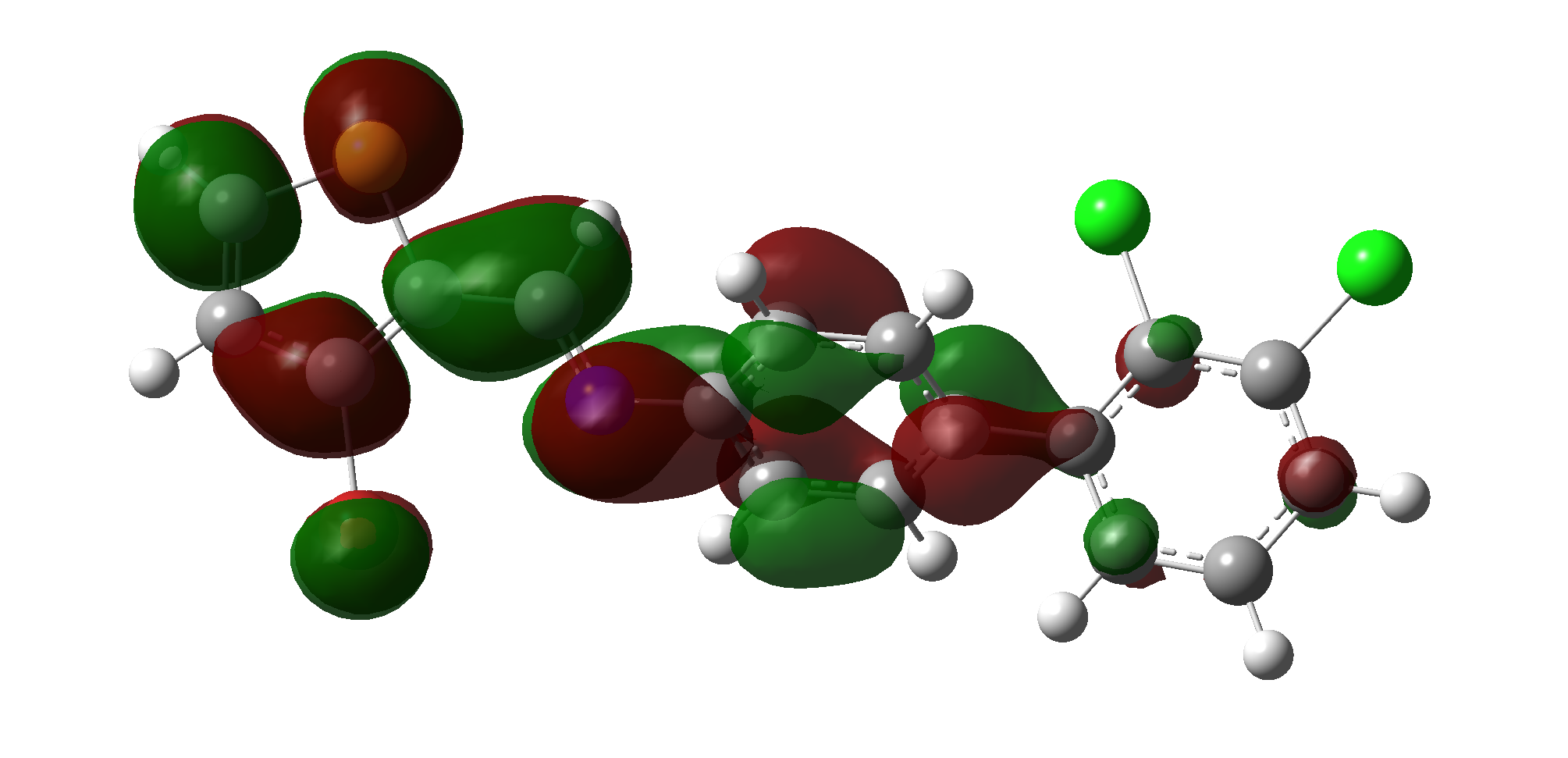  LUMO |
| 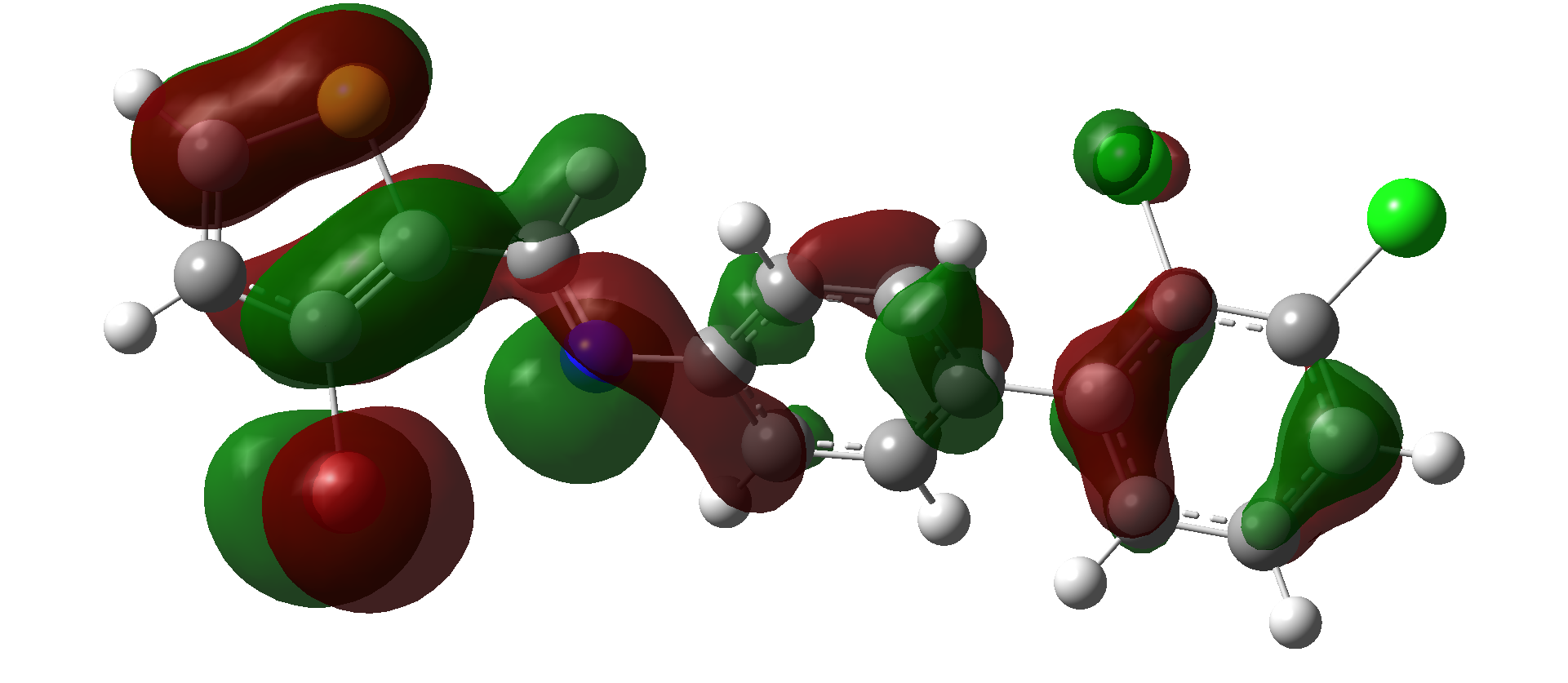  HOMO-1 | 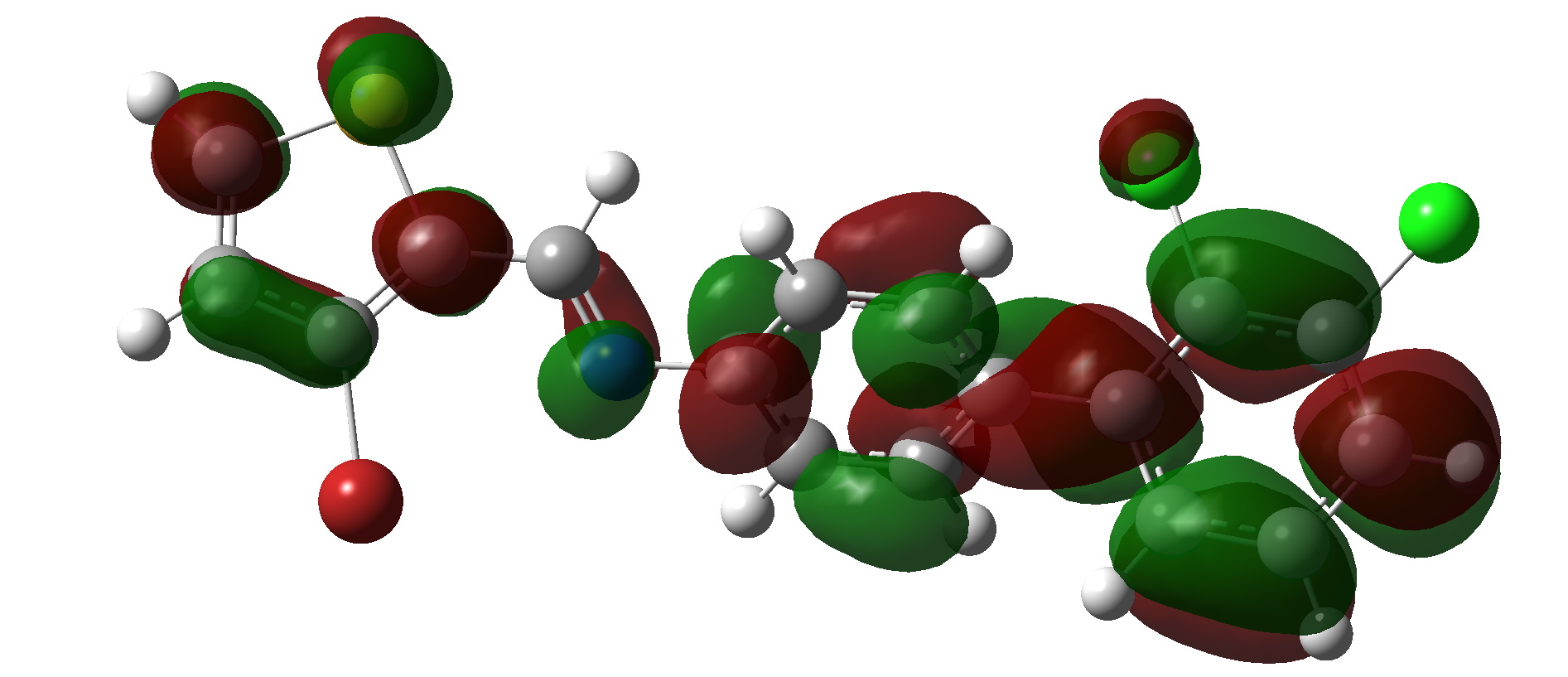  LUMO+1 |
| 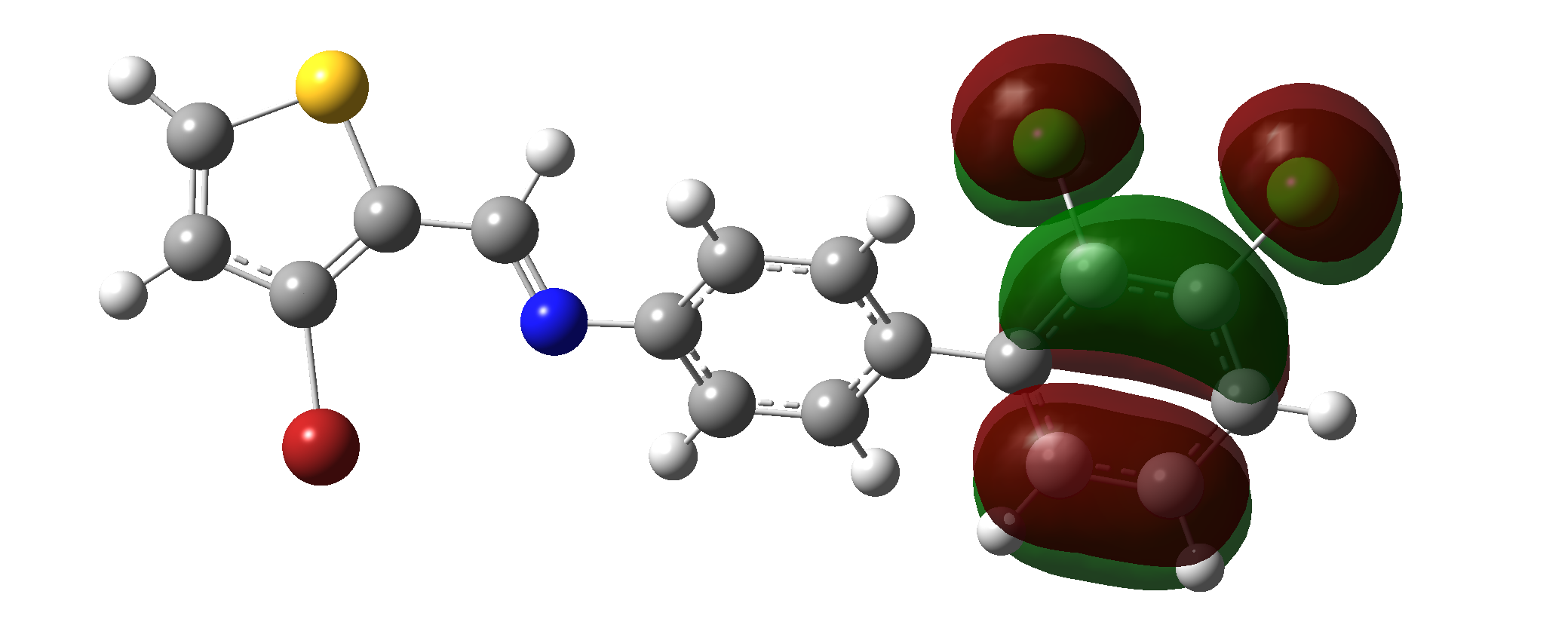  HOMO-2 | 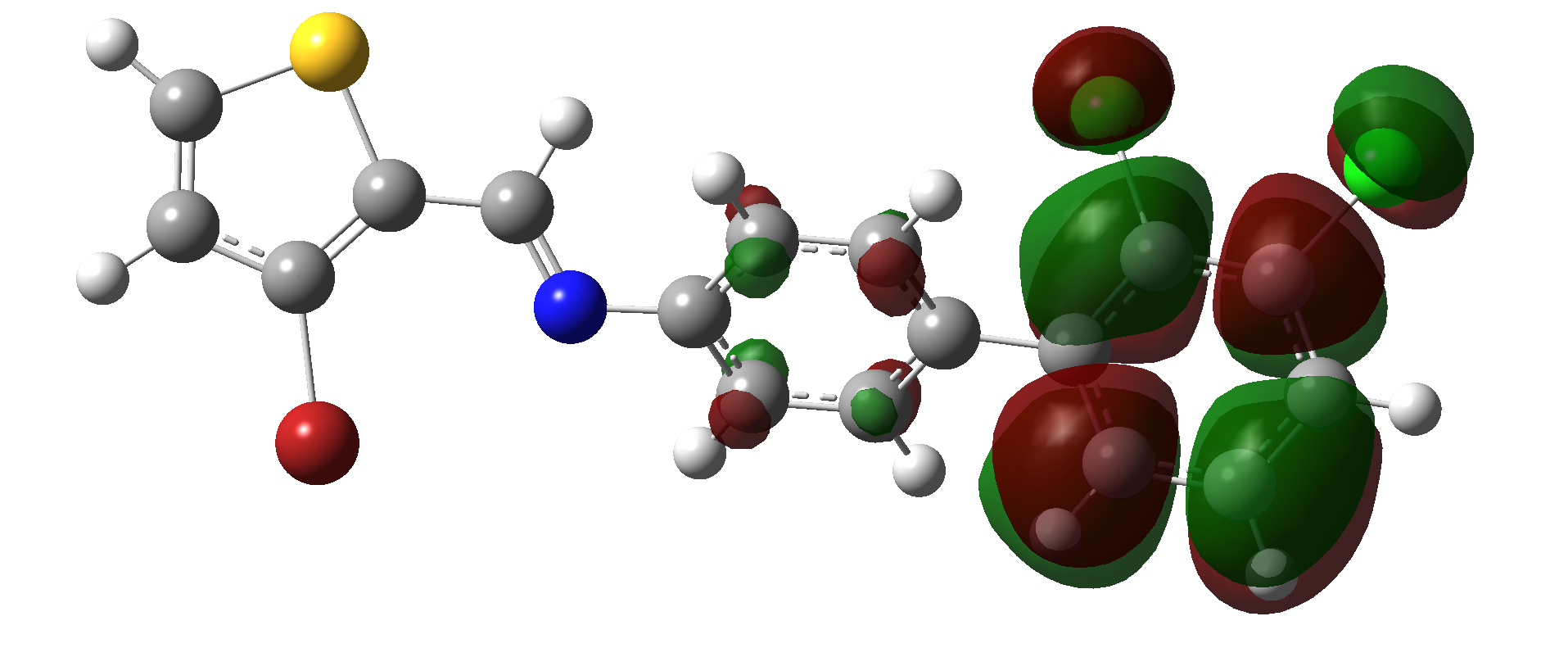  LUMO+2 |
| 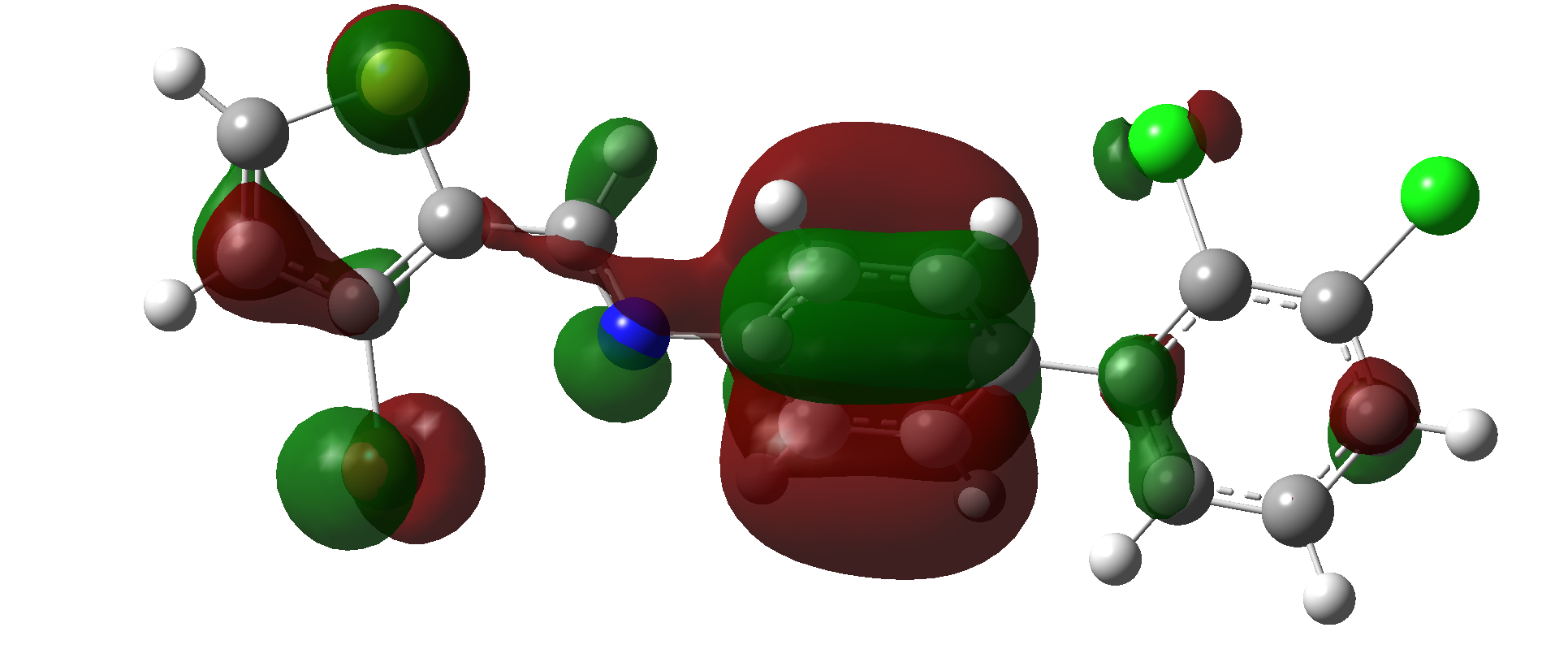  HOMO-3 | 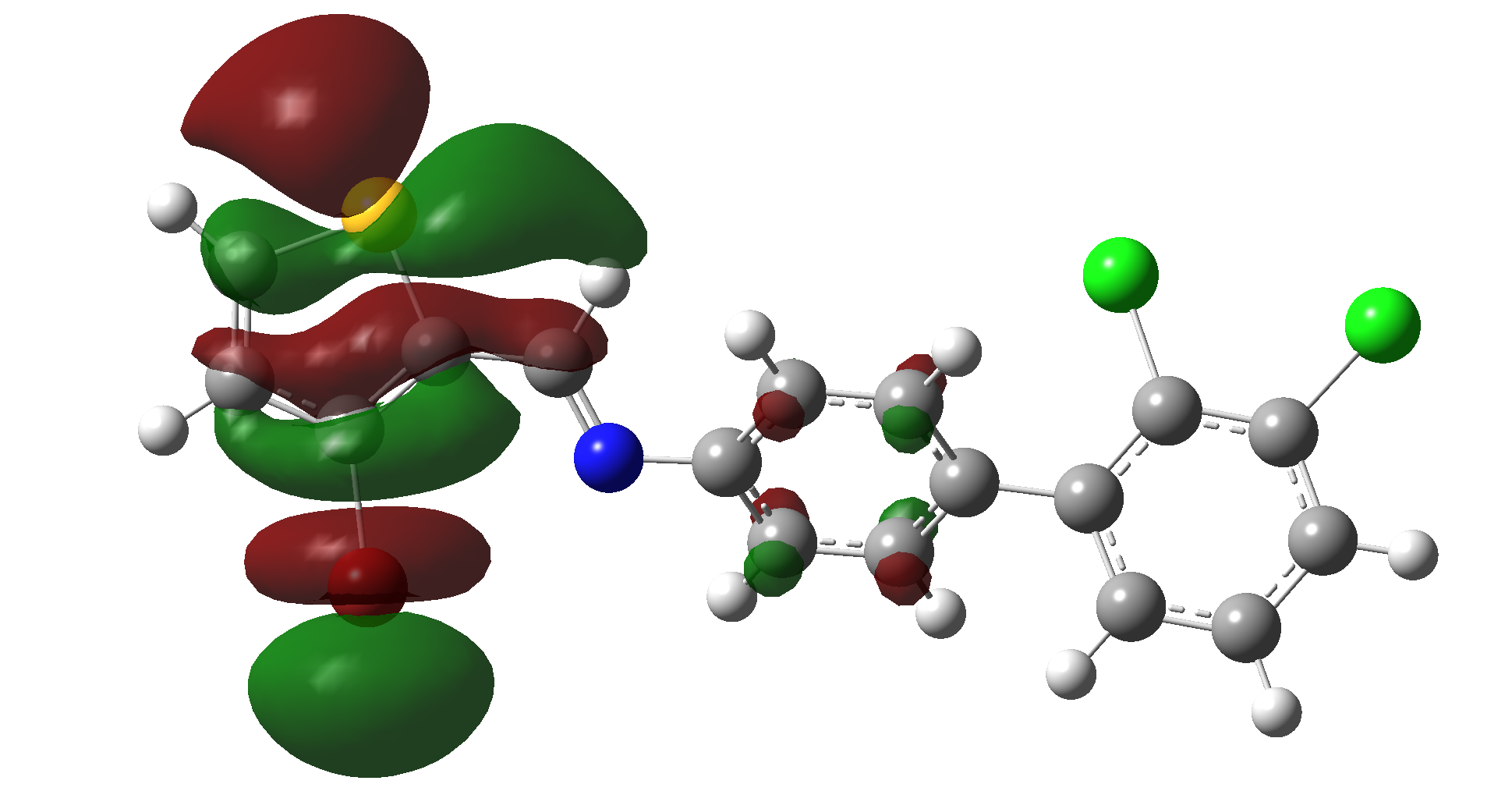  LUMO+3 |

| **3d** | |
| --- | --- |
| 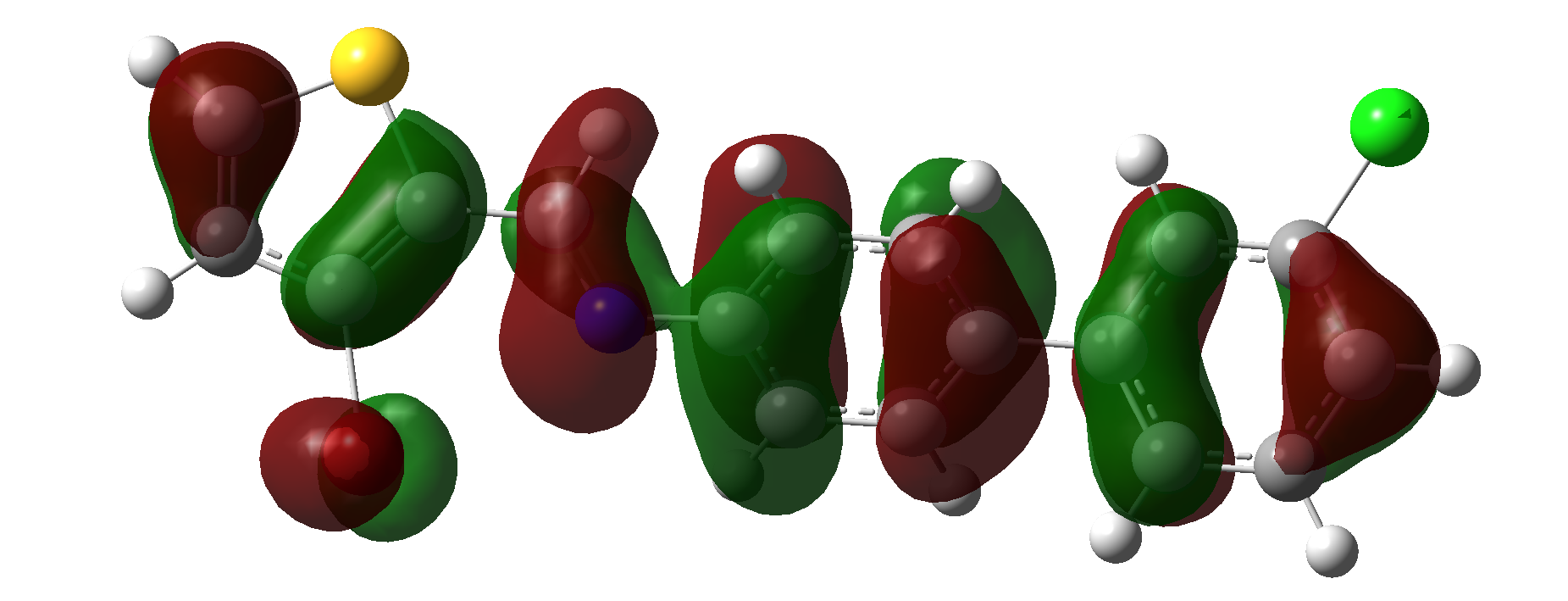  HOMO | 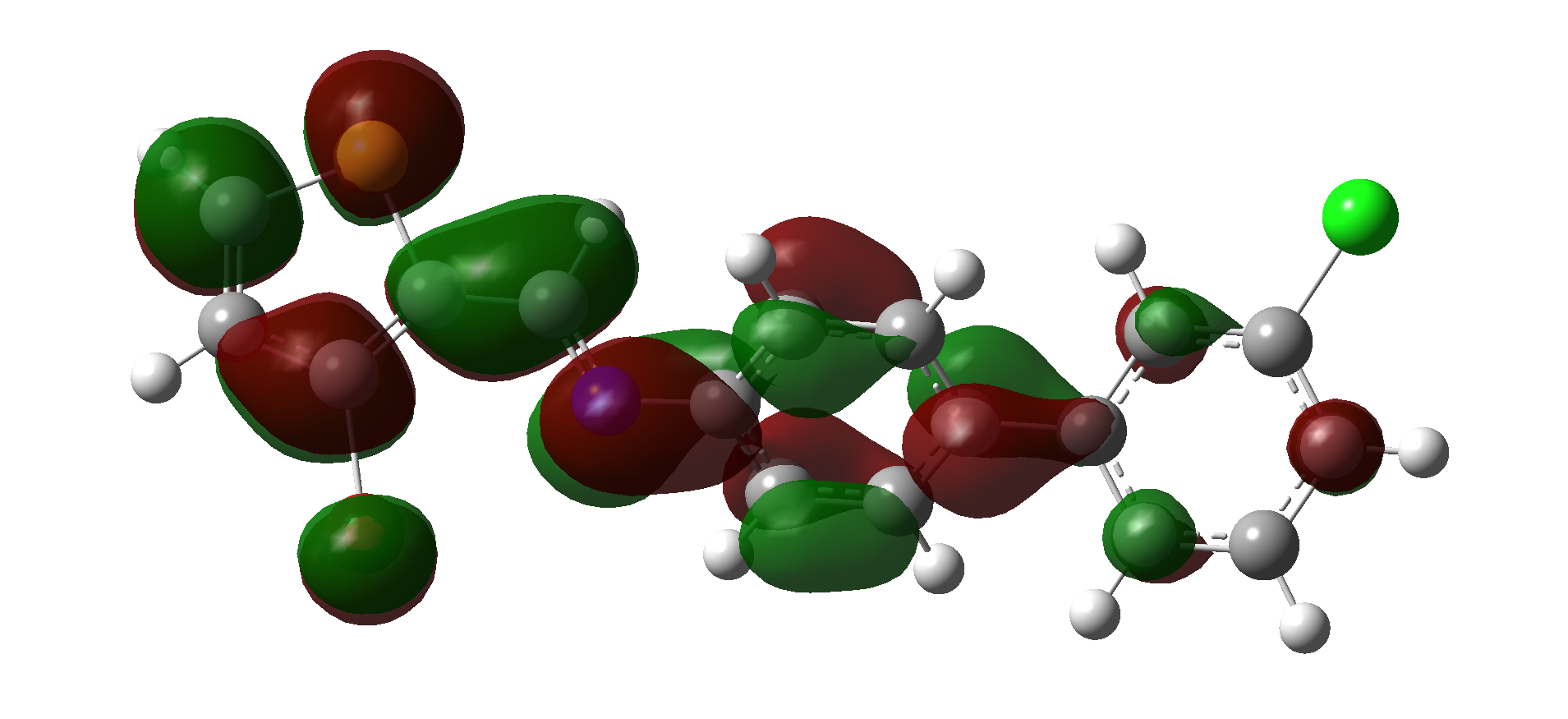  LUMO |
| 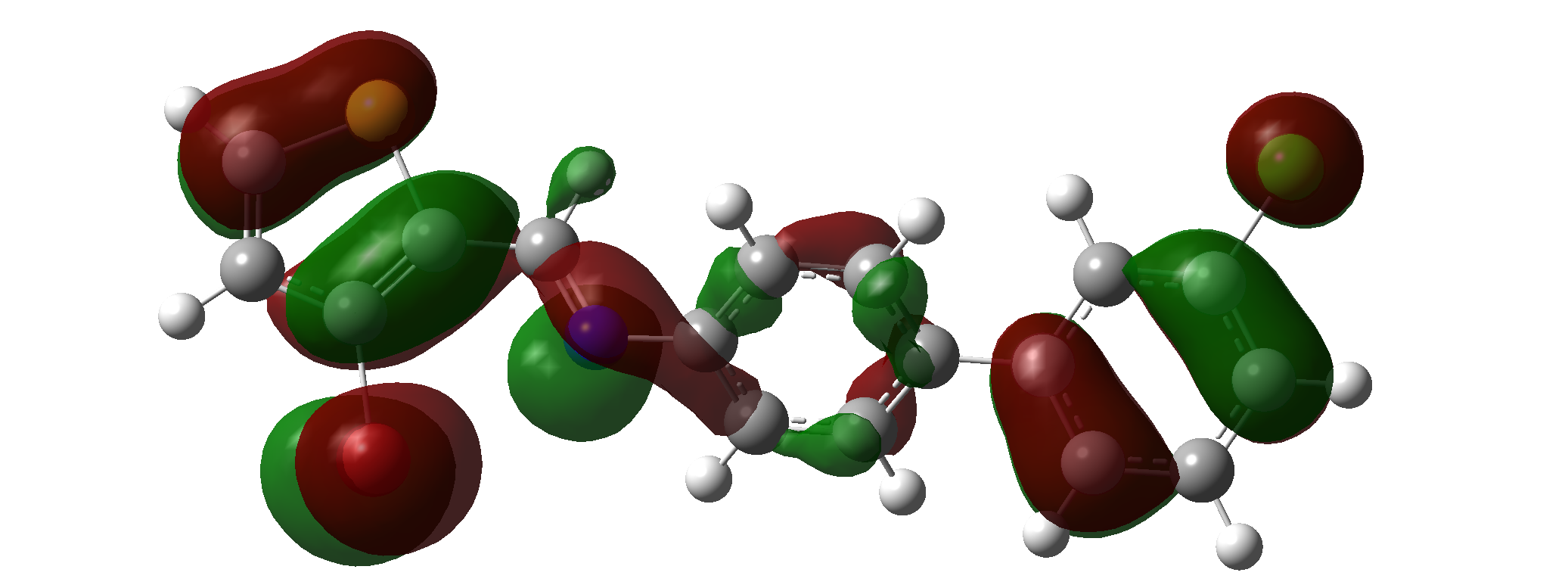  HOMO-1 | 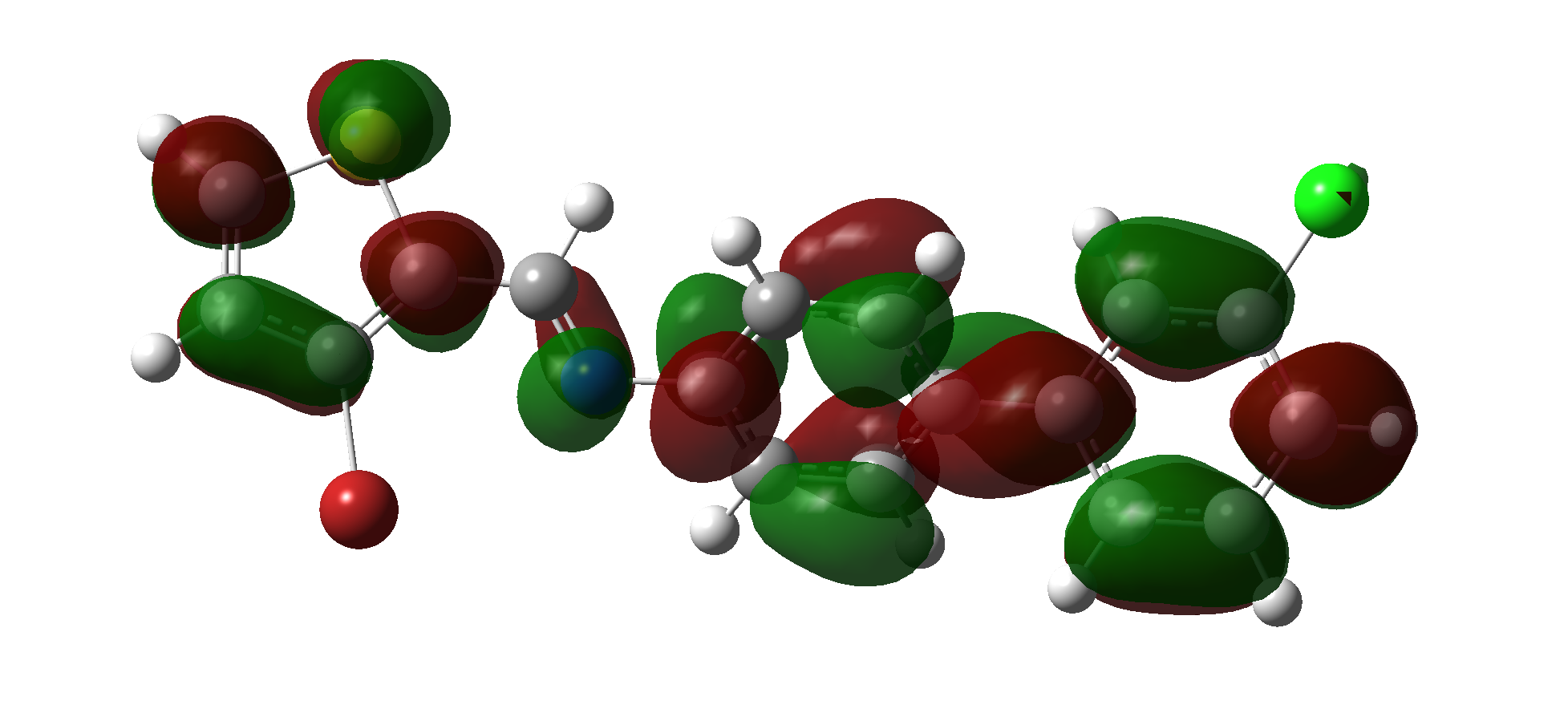  LUMO+1 |
| 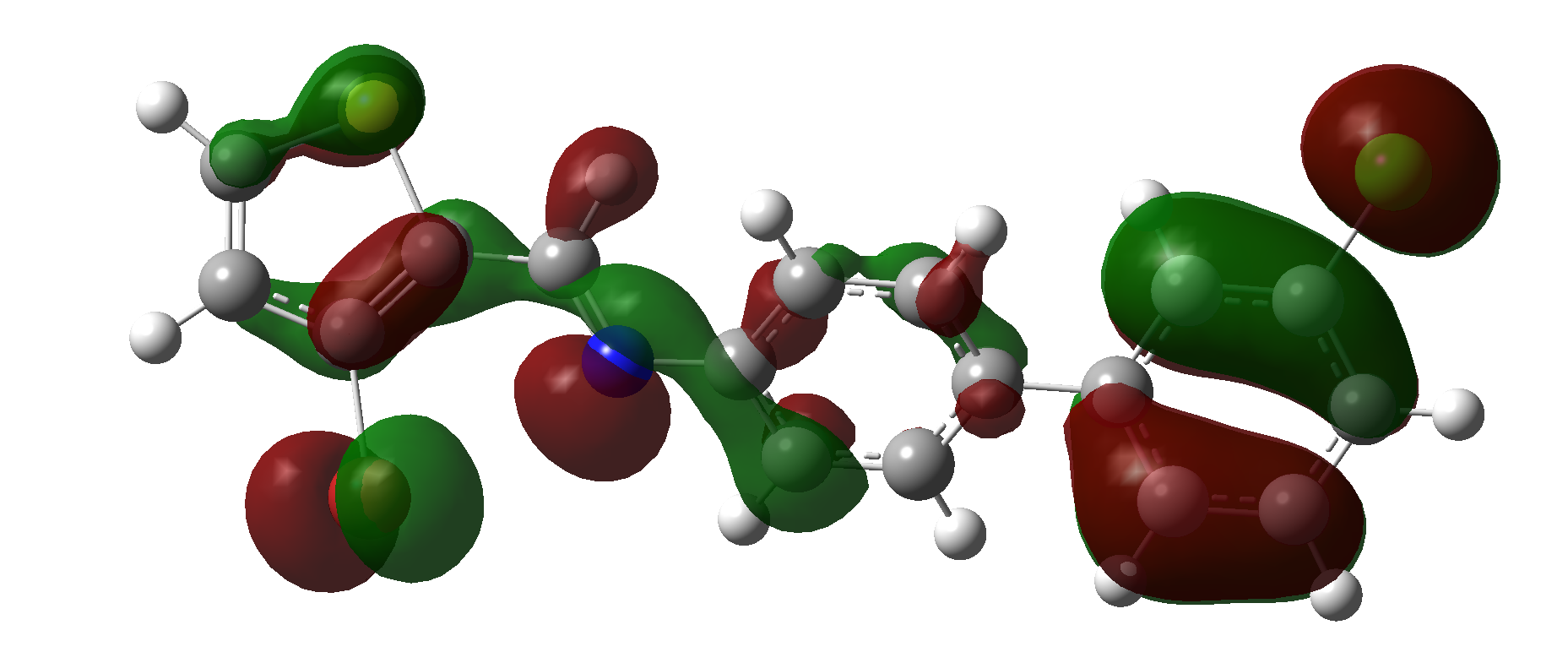  HOMO-2 | 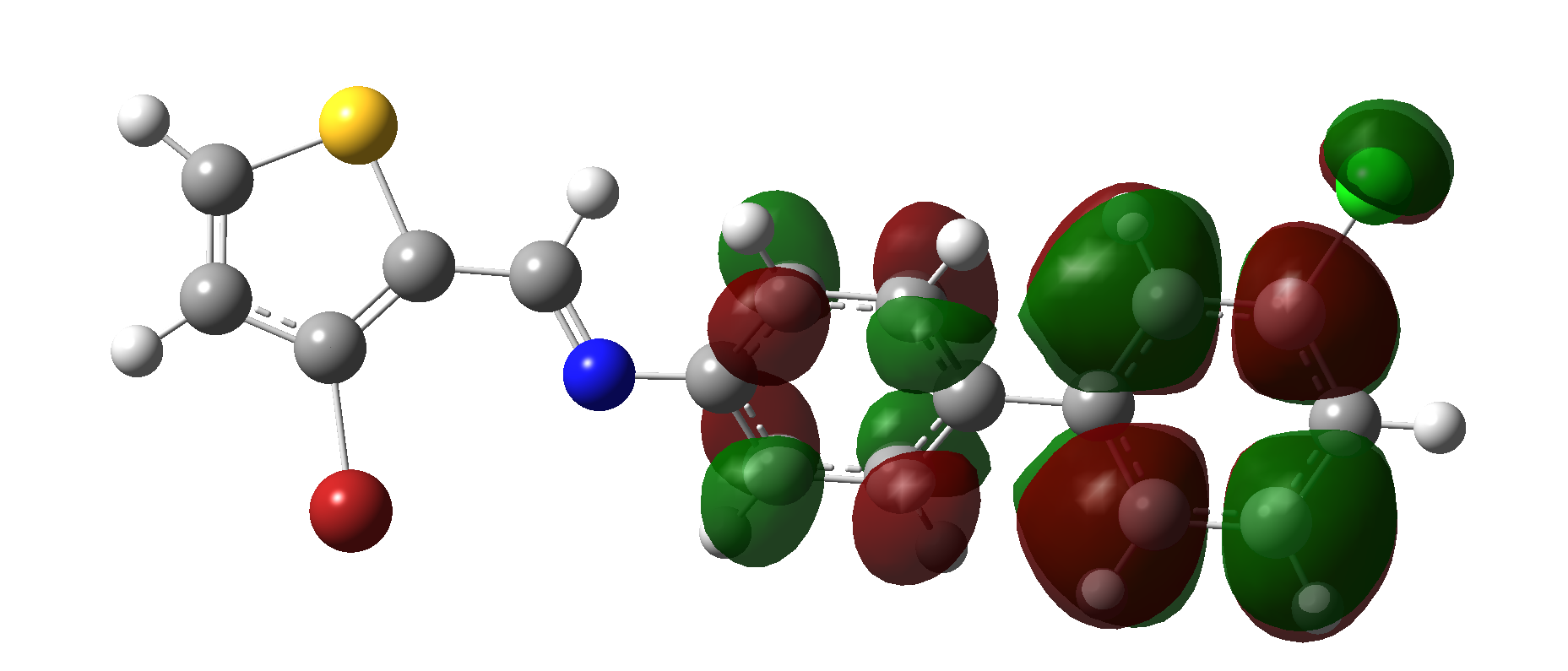  LUMO+2 |
| 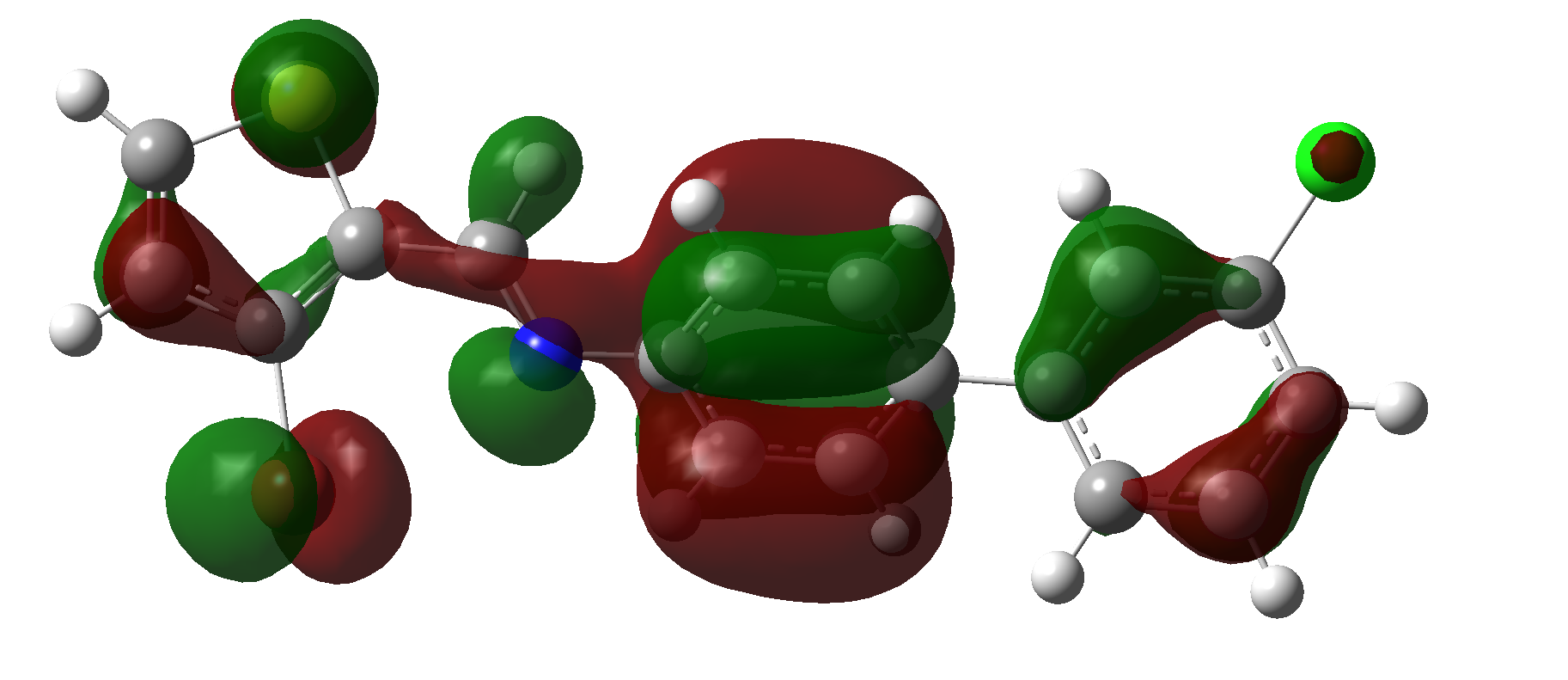  HOMO-3 | 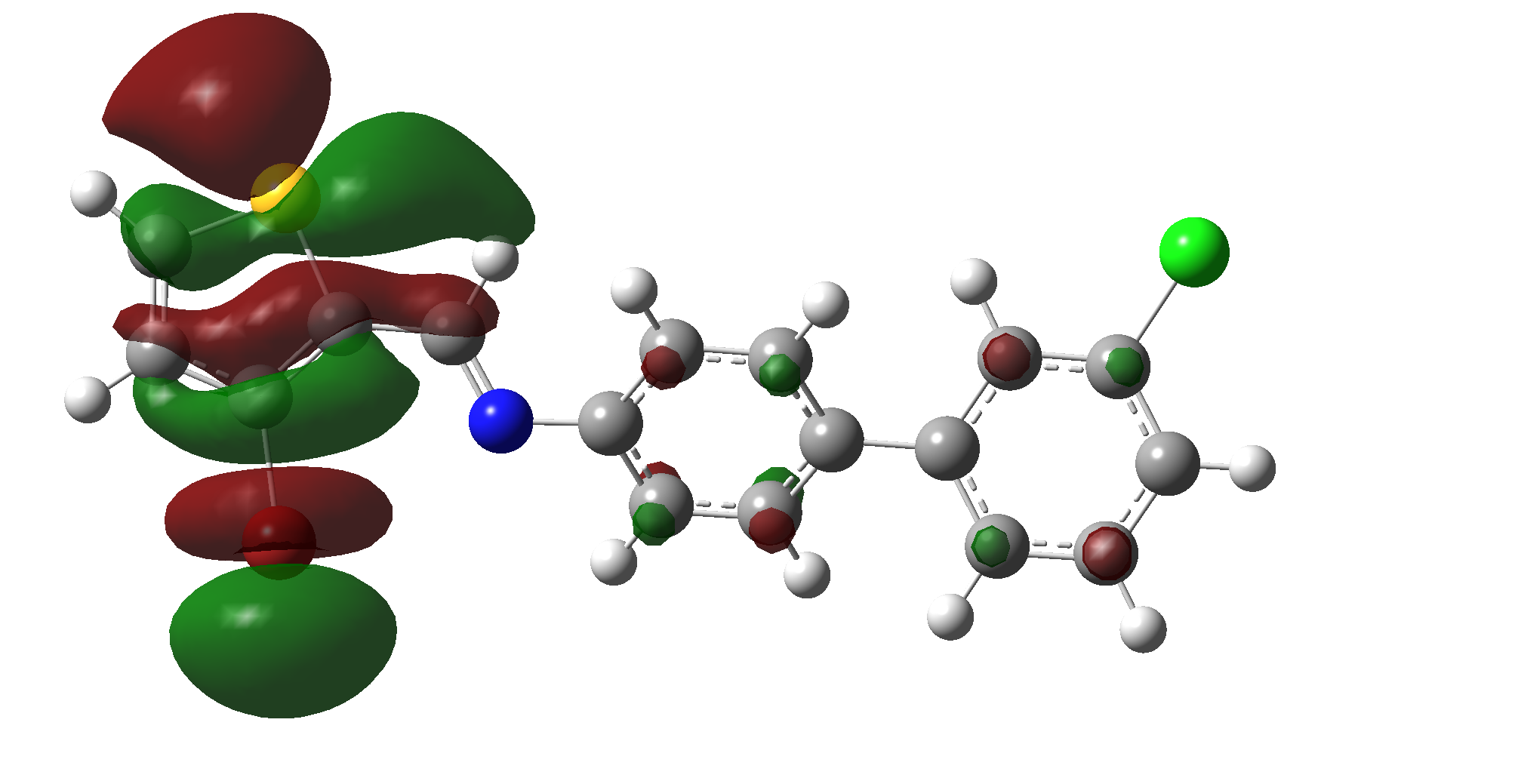  LUMO+3 |

| **3e** | |
| --- | --- |
| 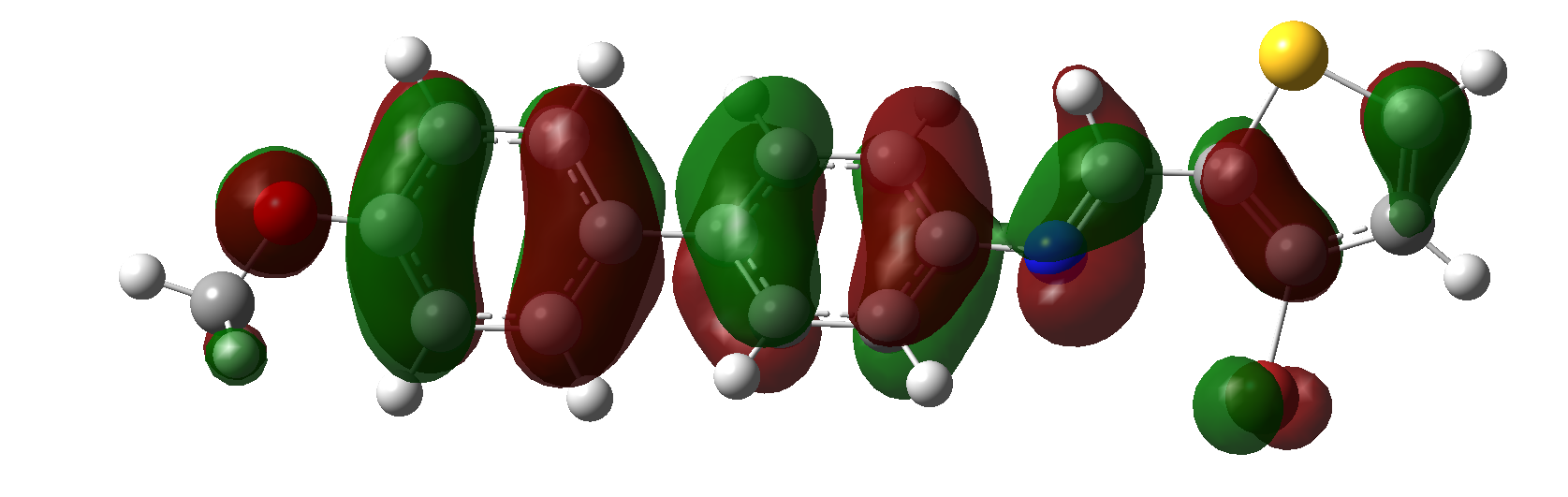  HOMO | 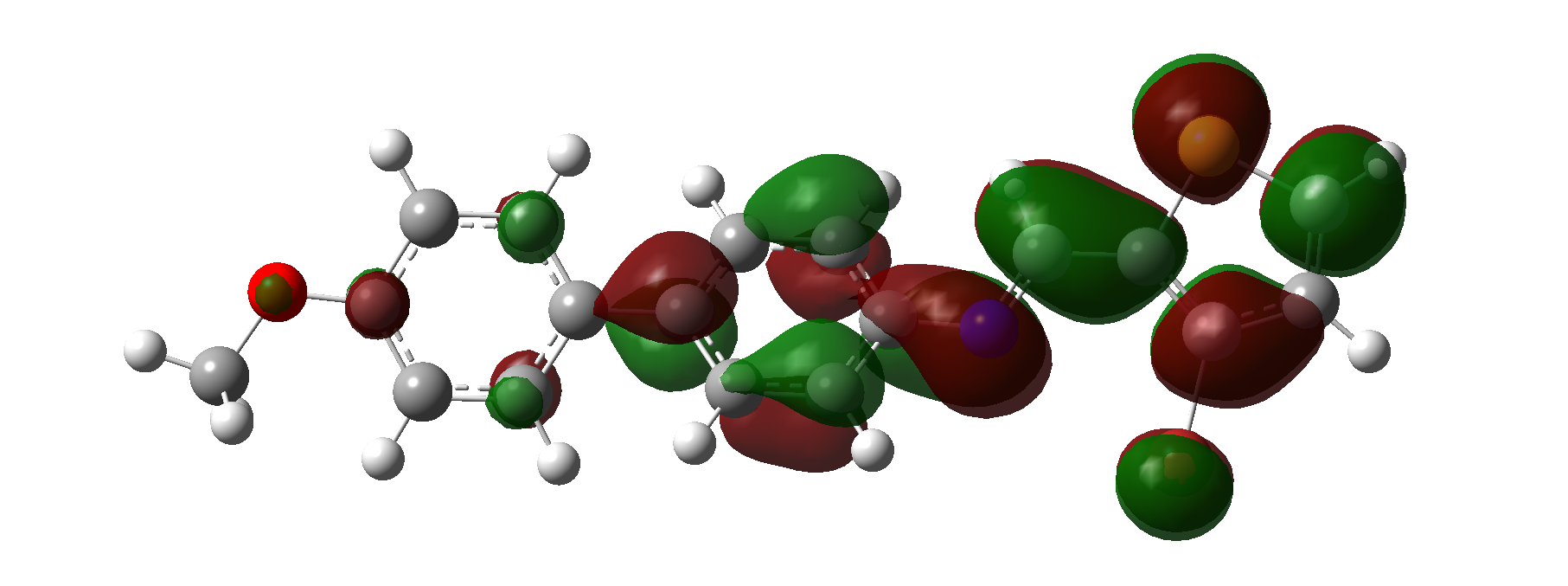  LUMO |
| 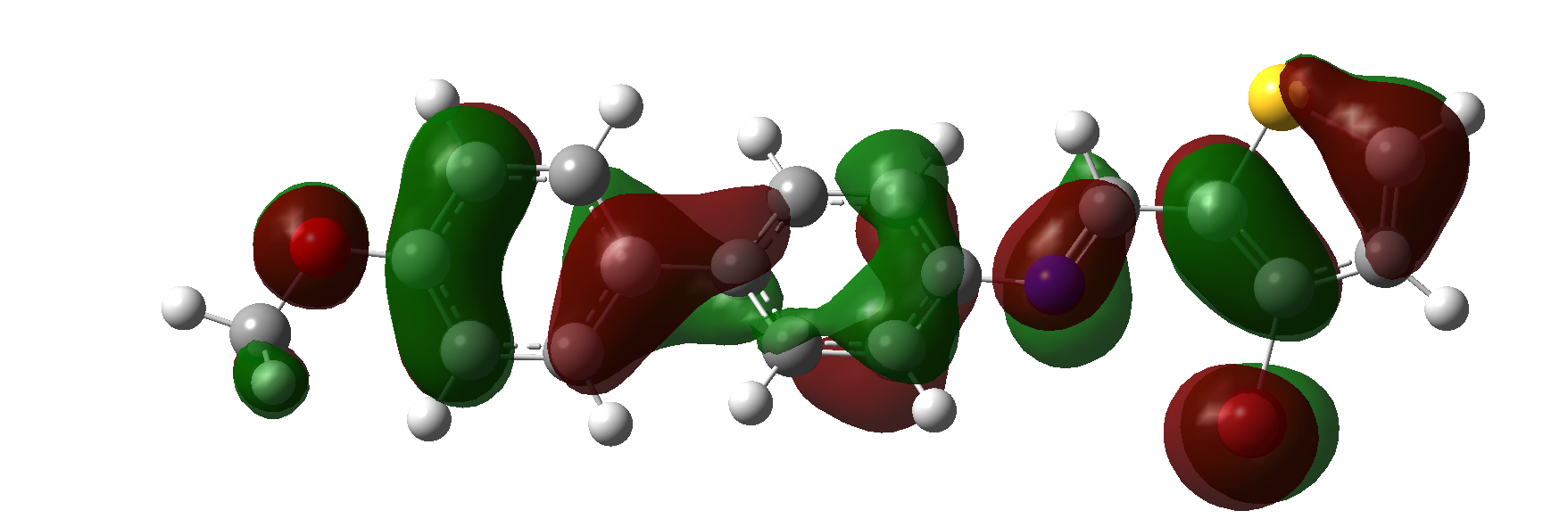  HOMO-1 | 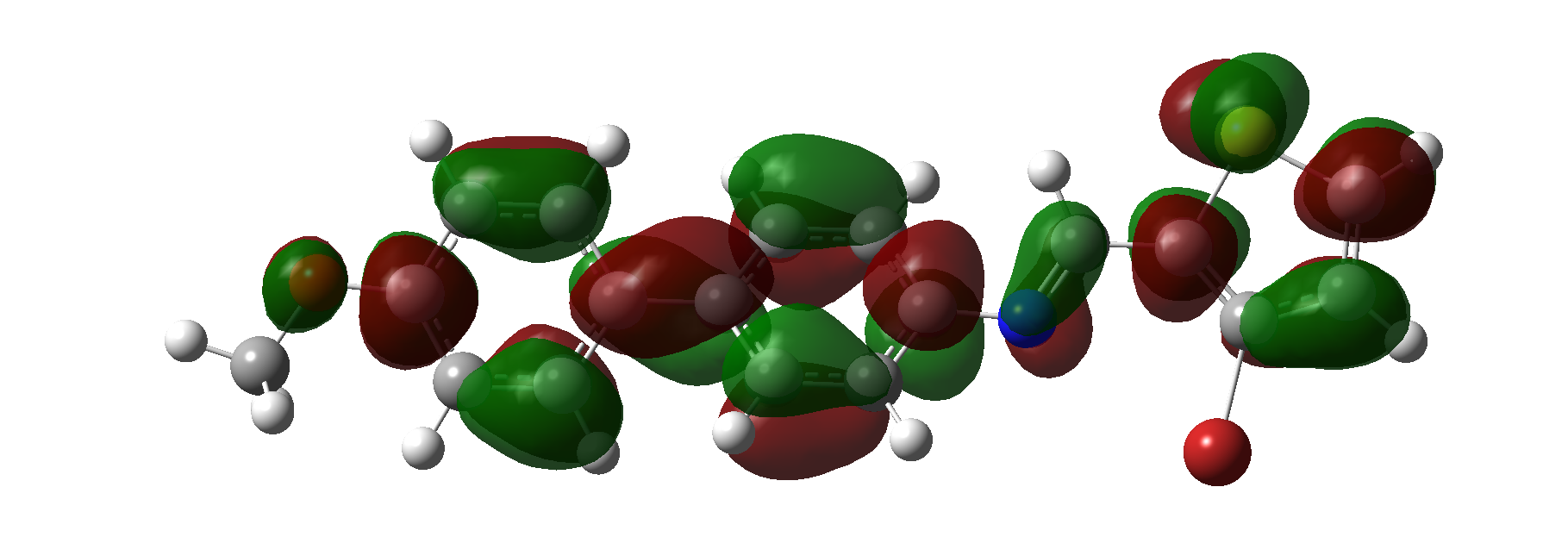  LUMO+1 |
| 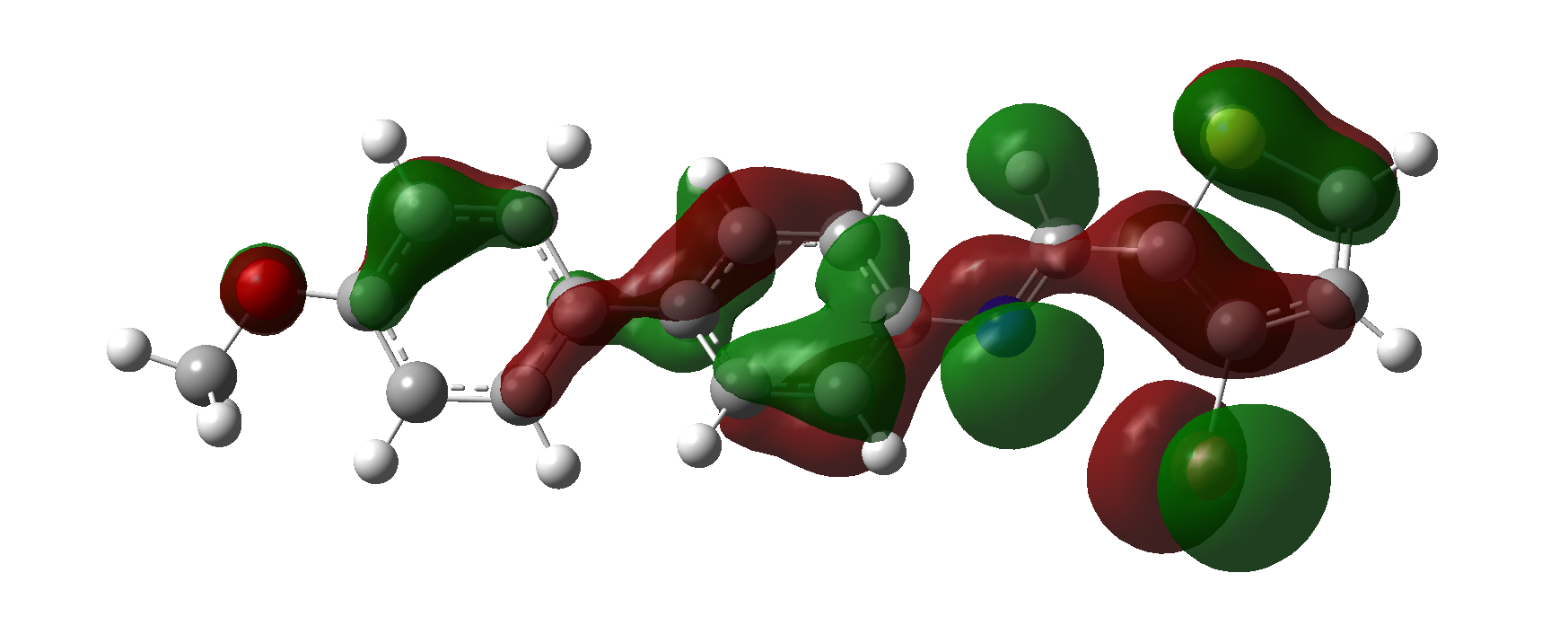  HOMO-2 | 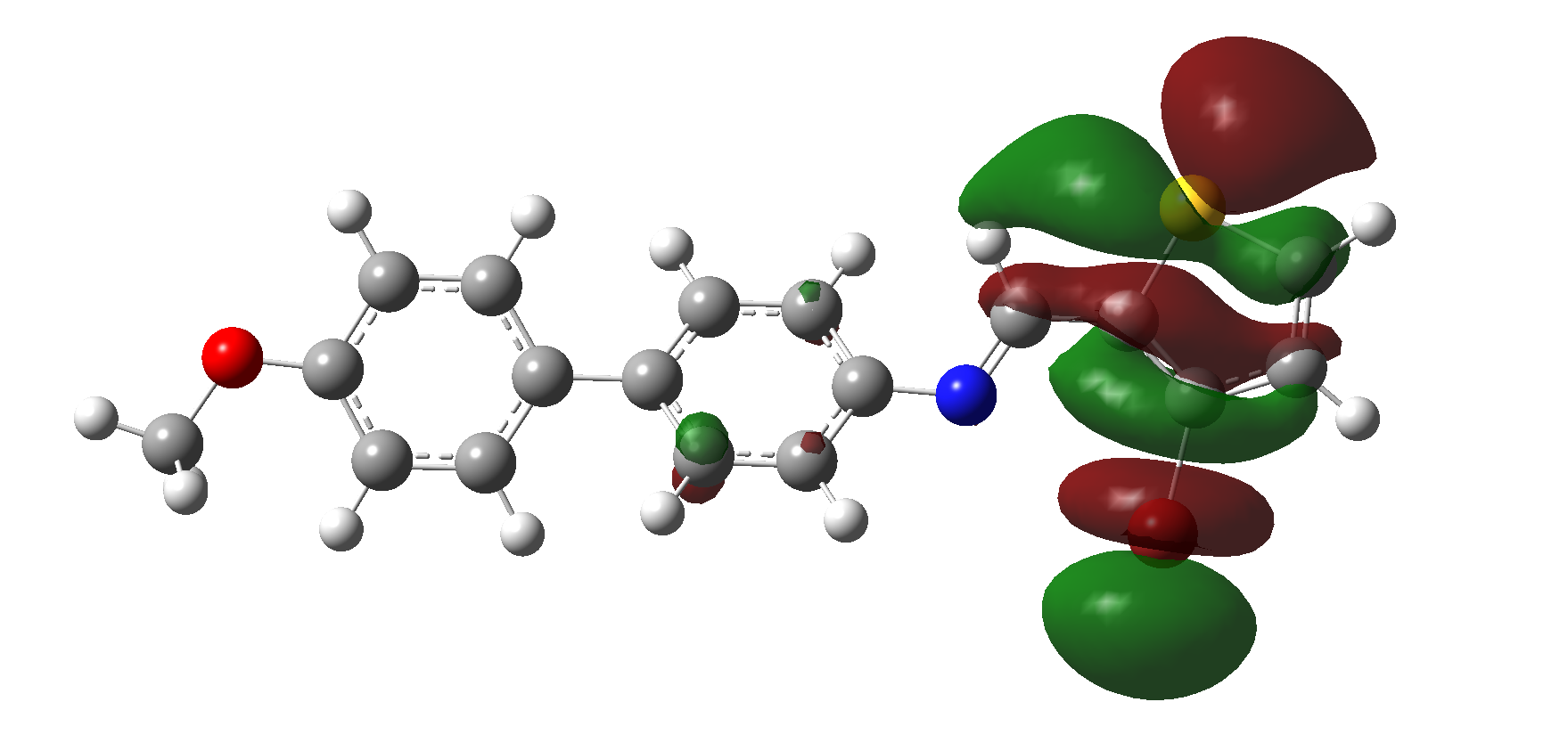  LUMO+2 |
| 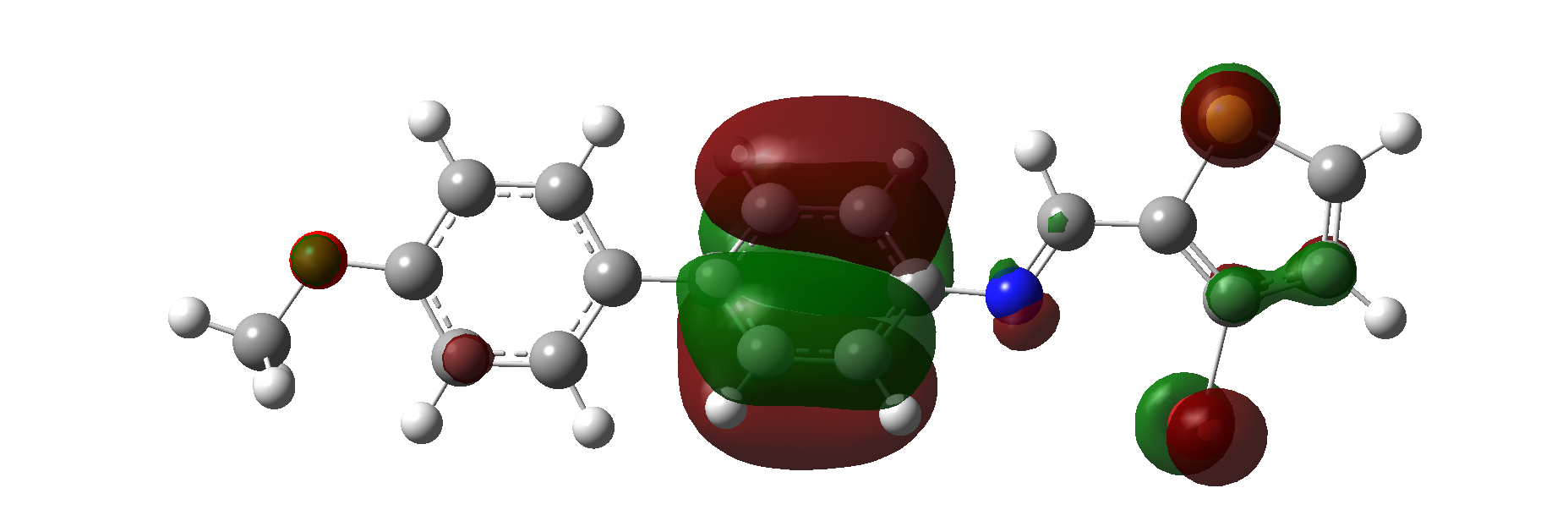    HOMO-3 | 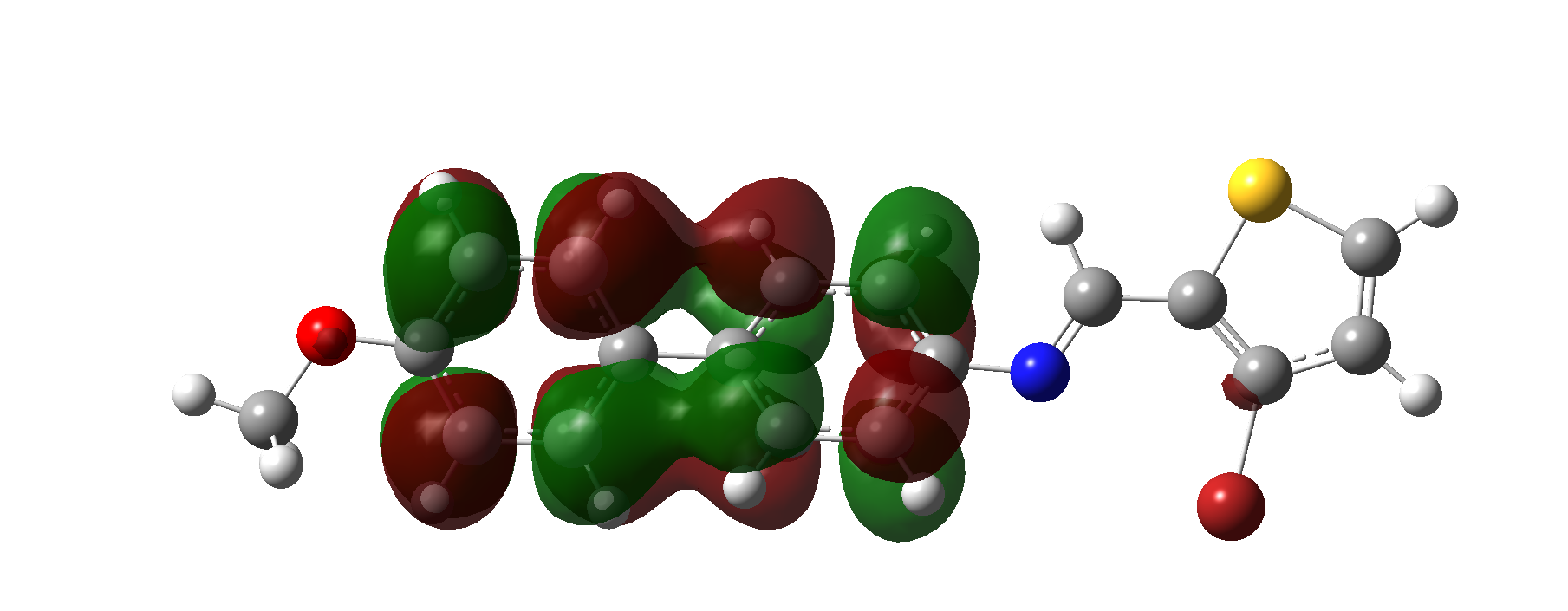  LUMO+3 |

| **3f** | |
| --- | --- |
| 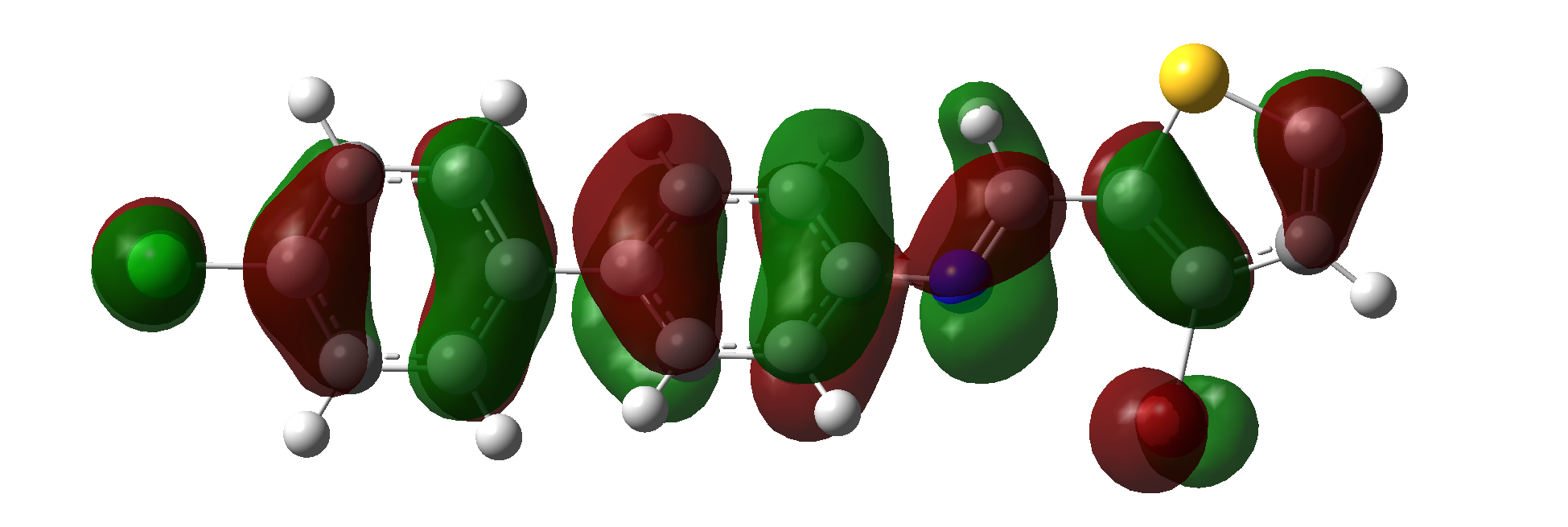  HOMO | 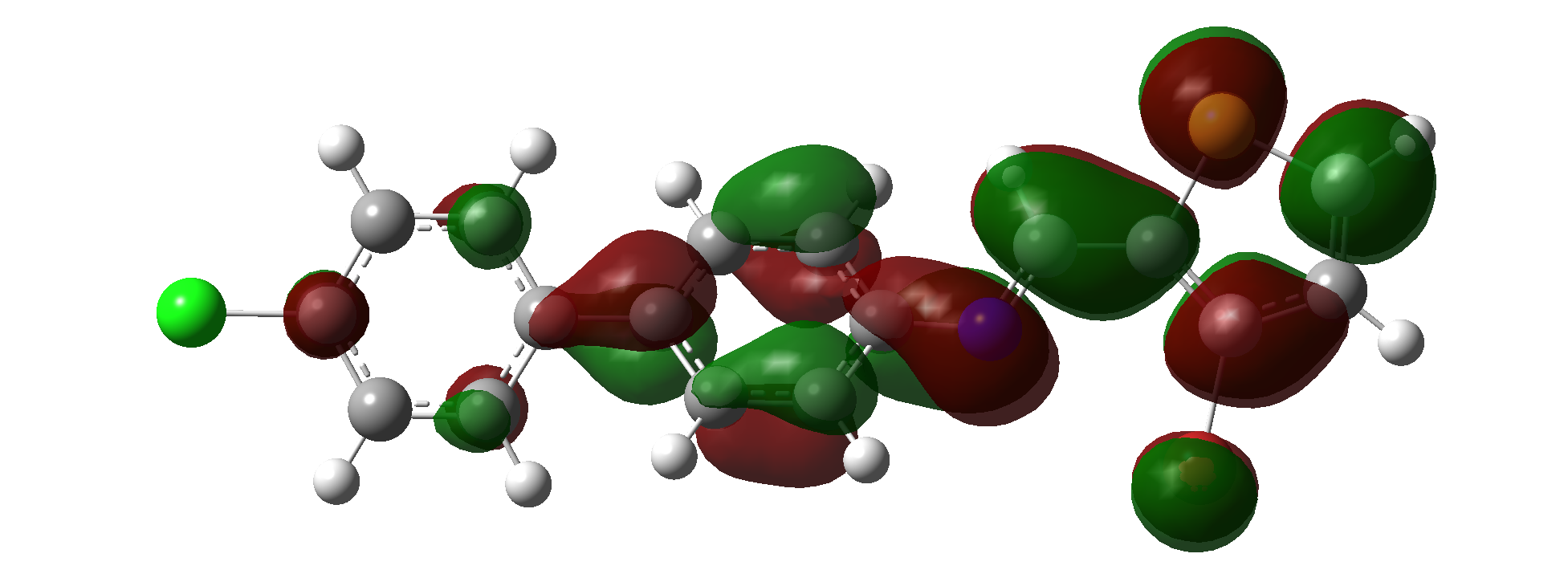  LUMO |
| 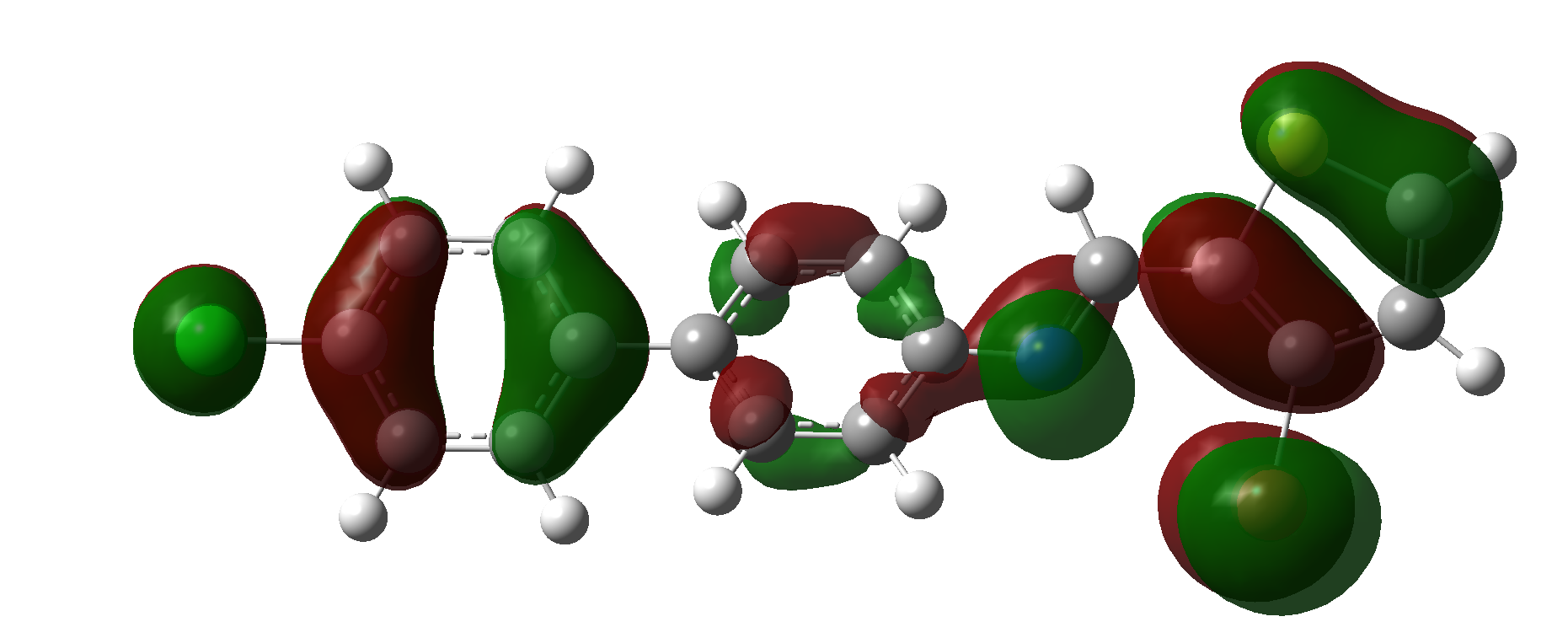  HOMO-1 | 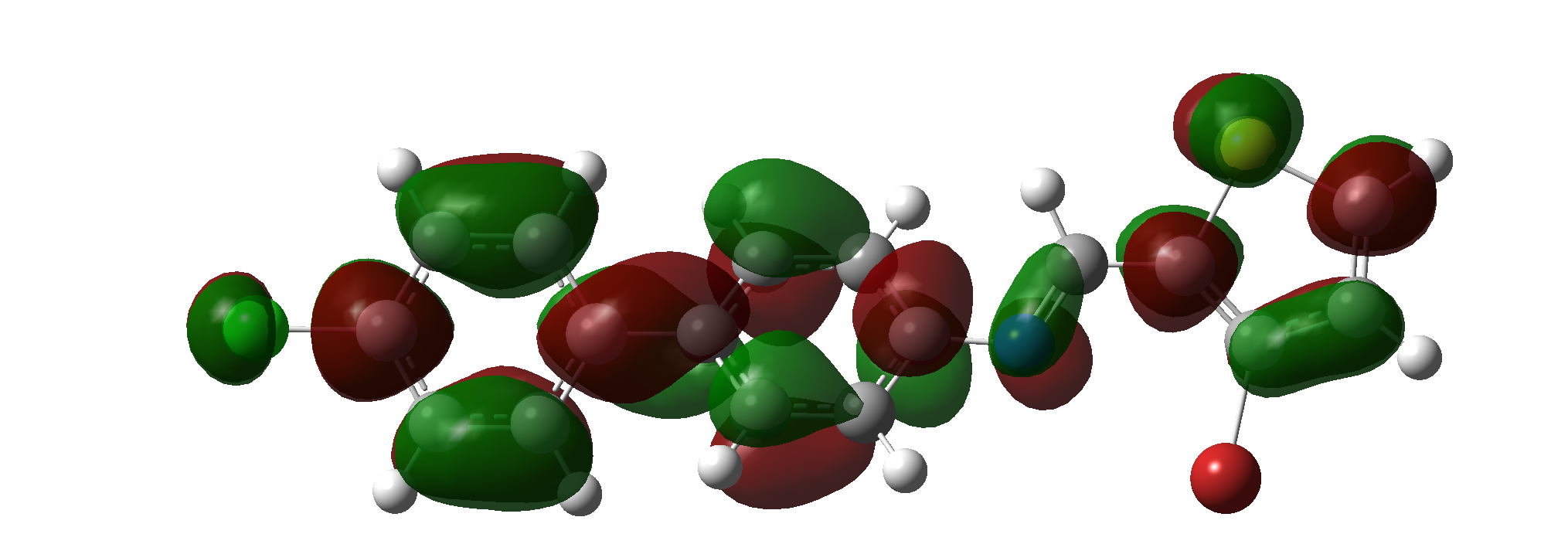  LUMO+1 |
| 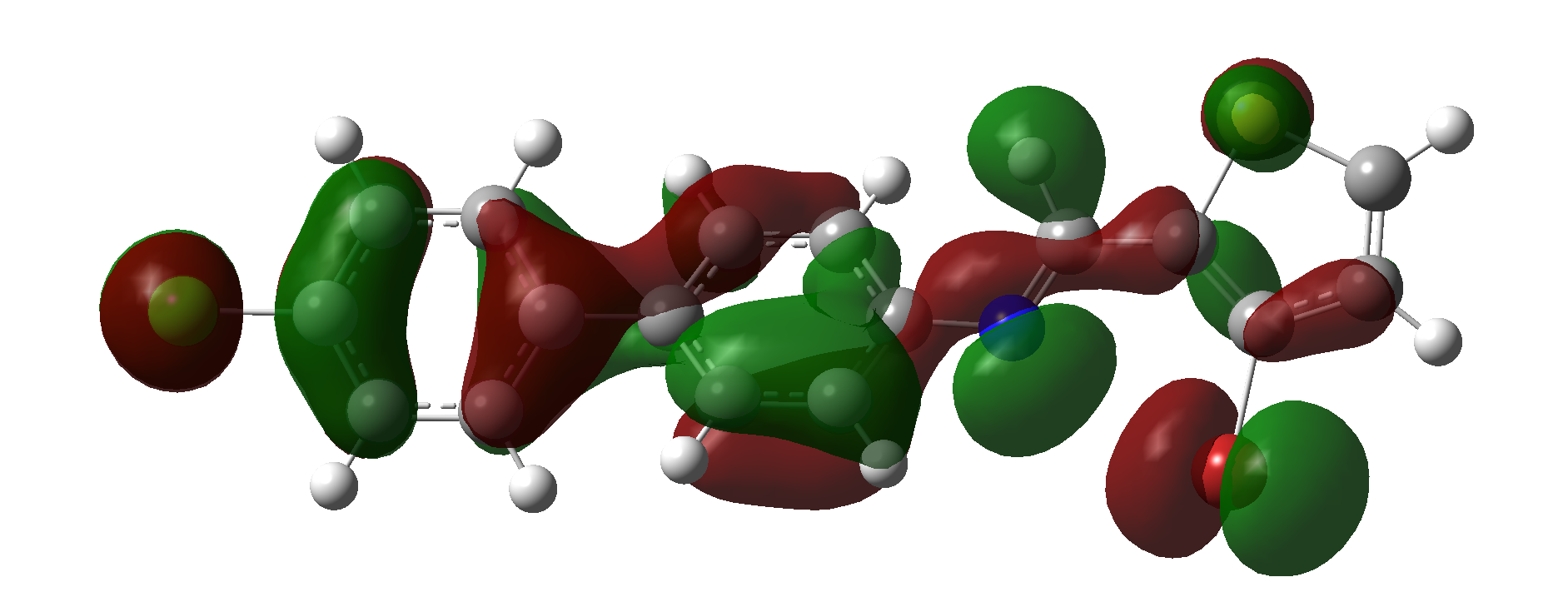  HOMO-2 | 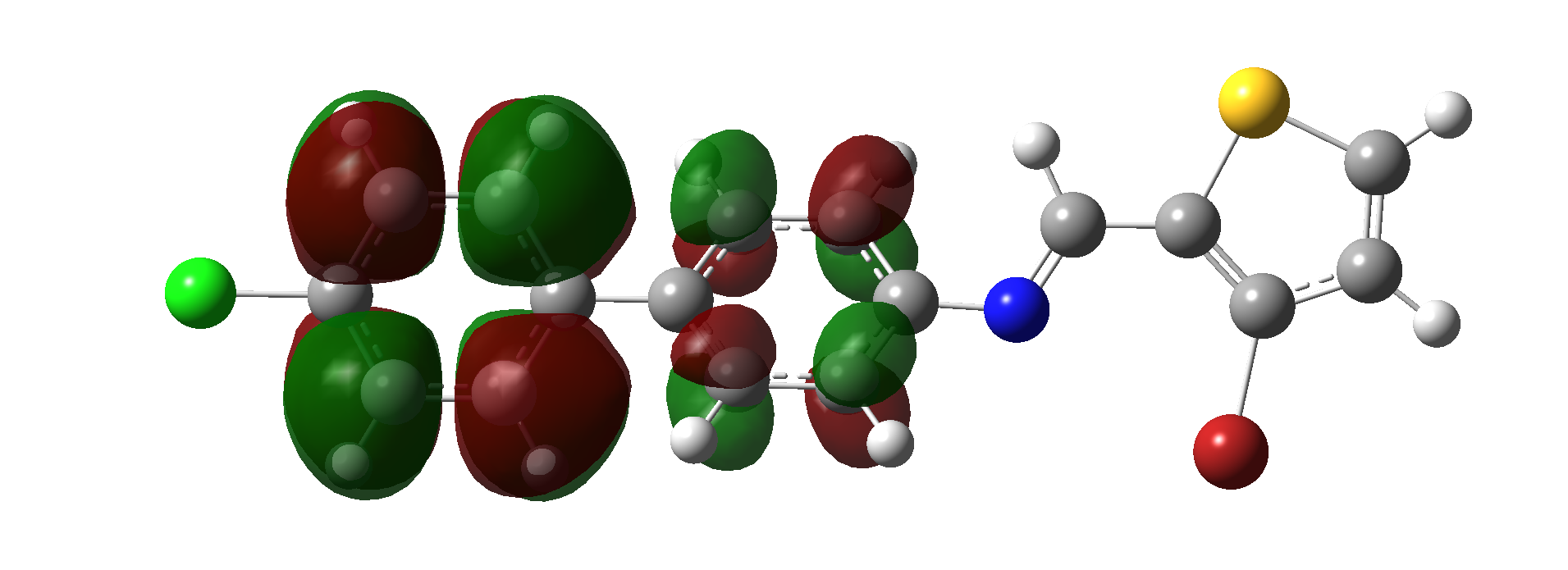  LUMO+2 |
| 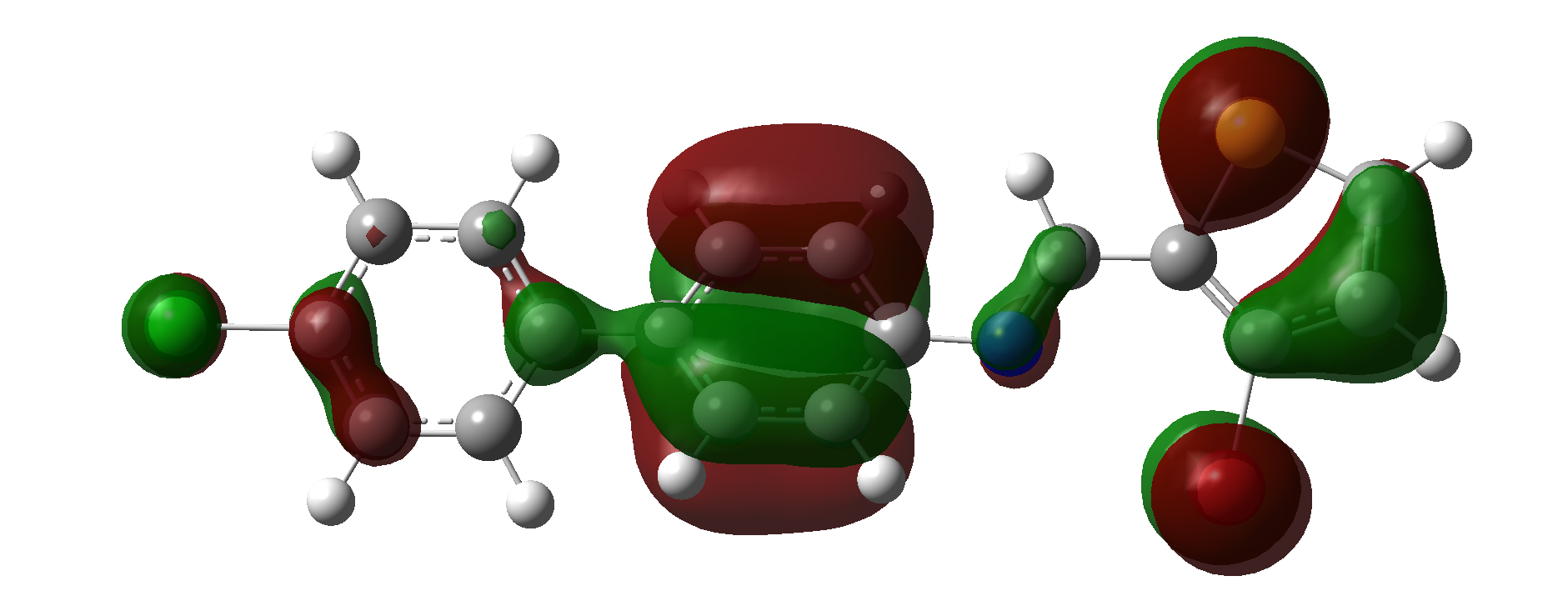  HOMO-3 | 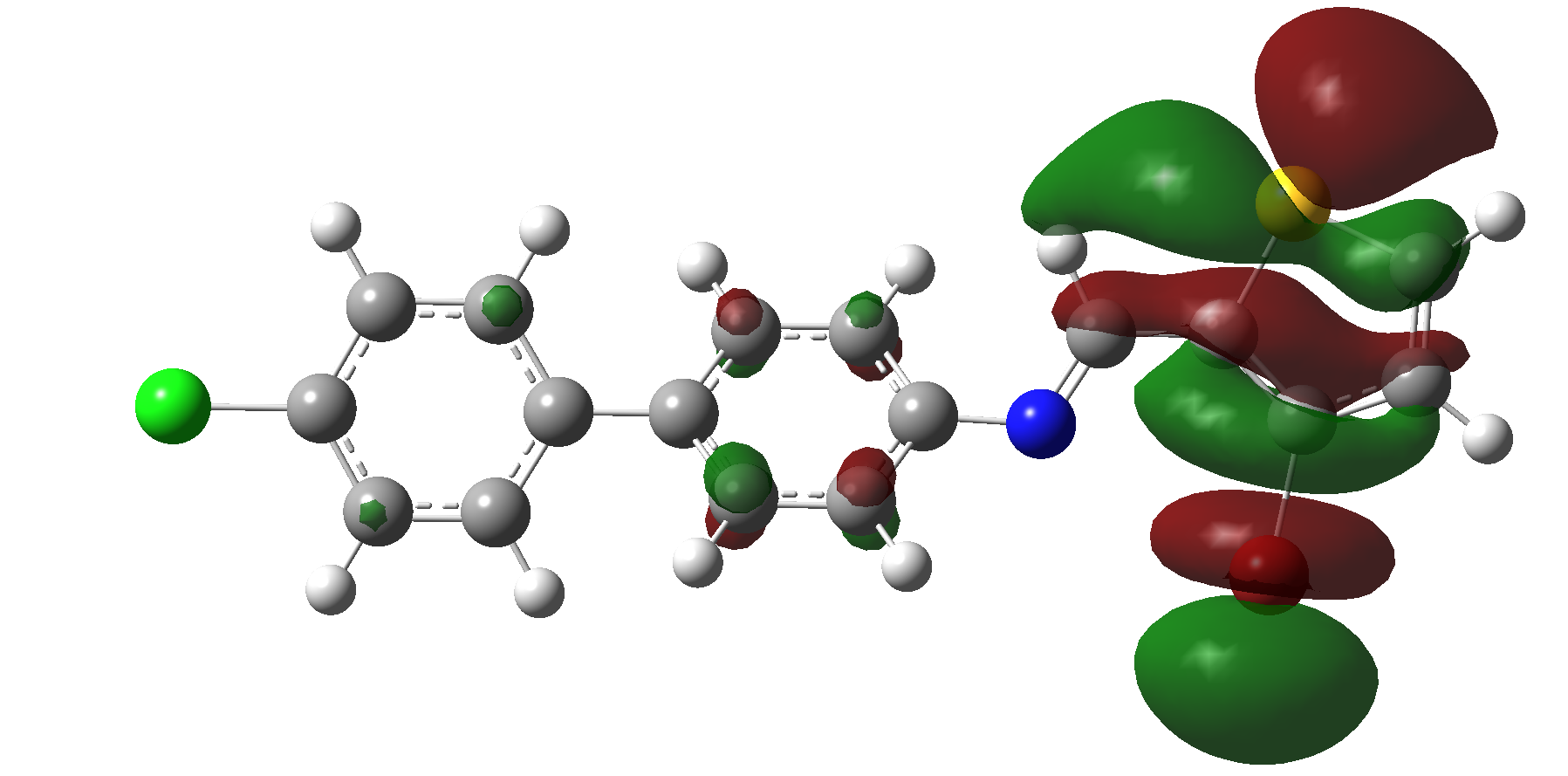  LUMO+3 |

| **3g** | |
| --- | --- |
| 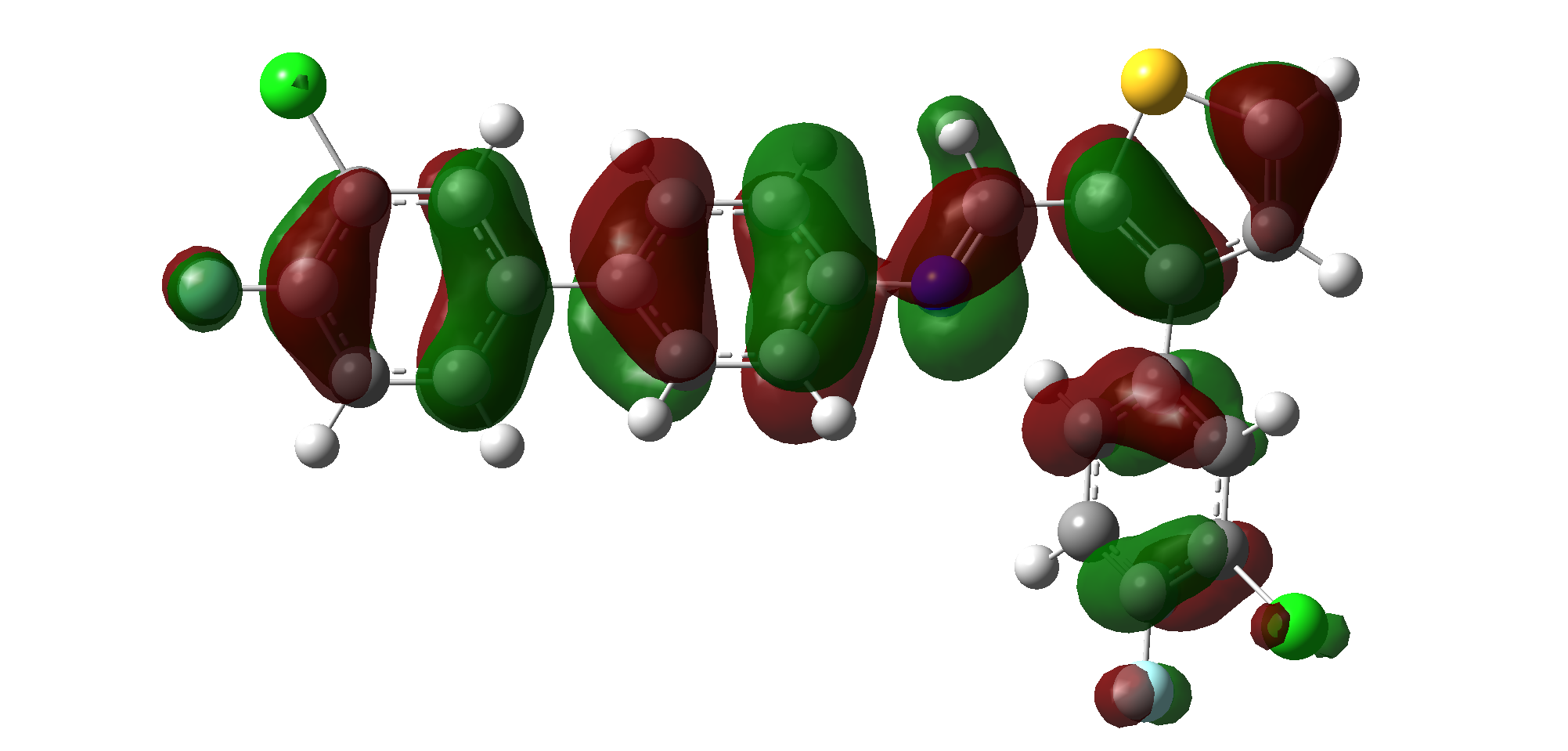  HOMO | 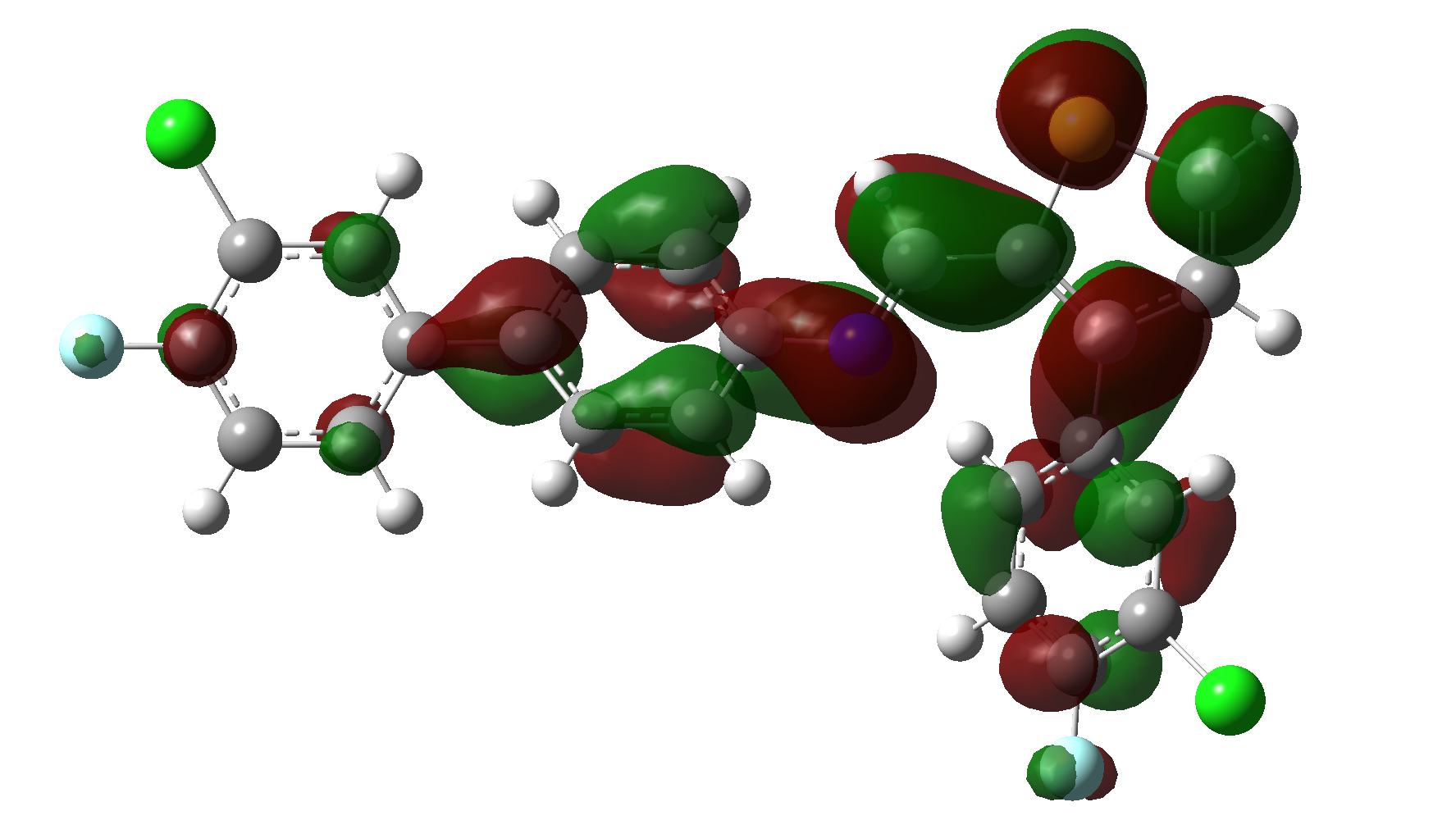  LUMO |
| 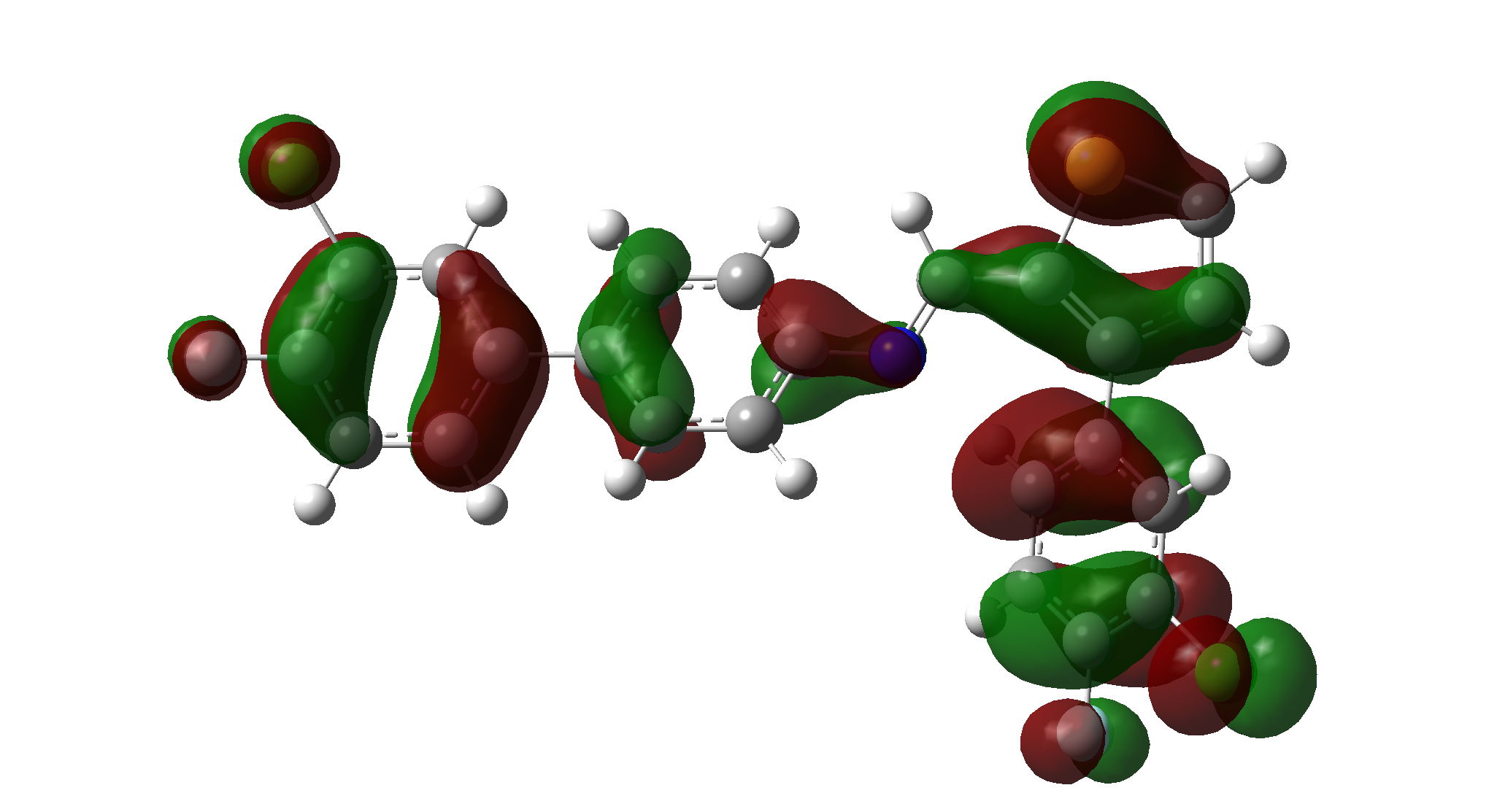  HOMO-1 | 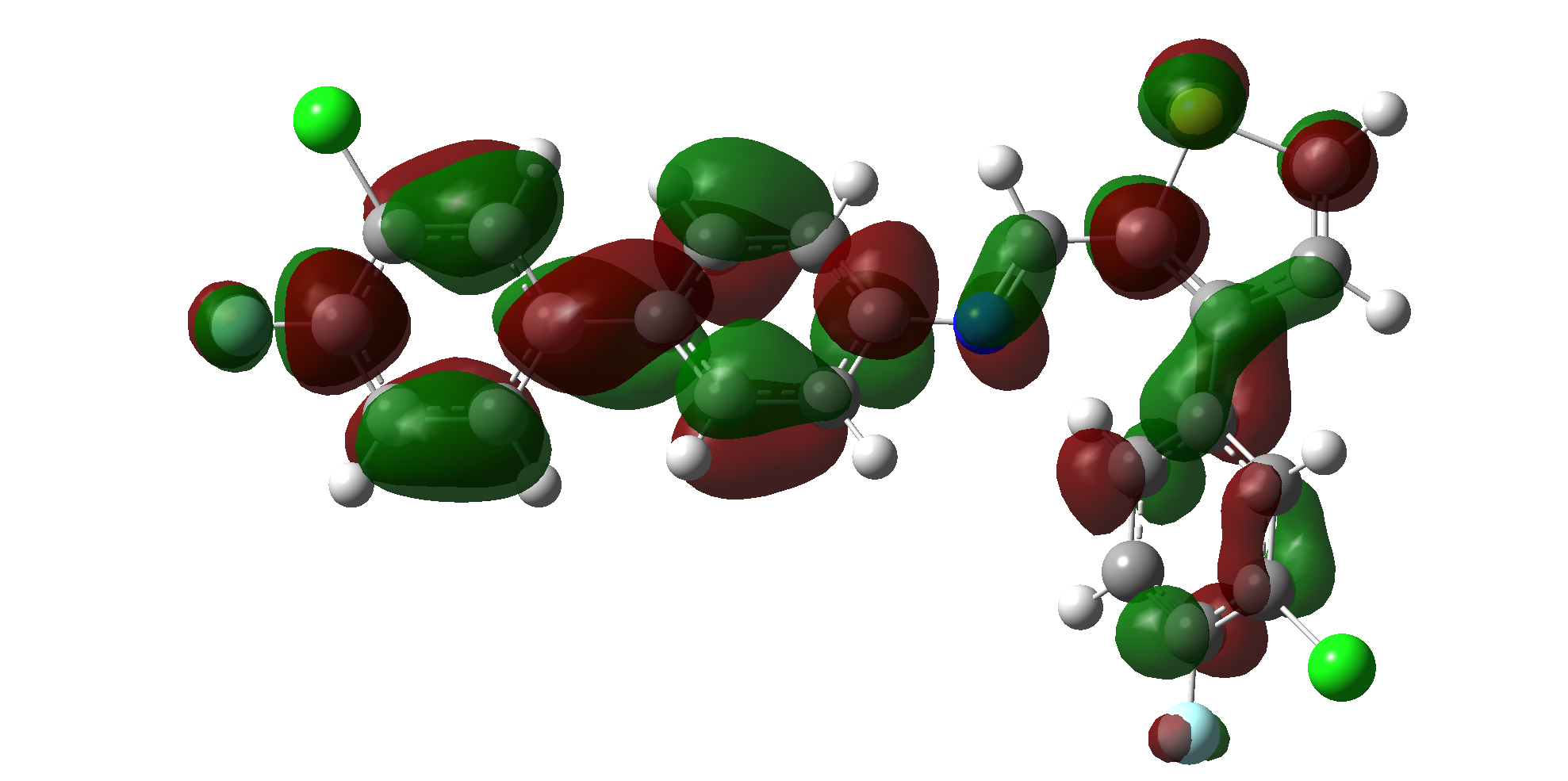  LUMO+1 |
| 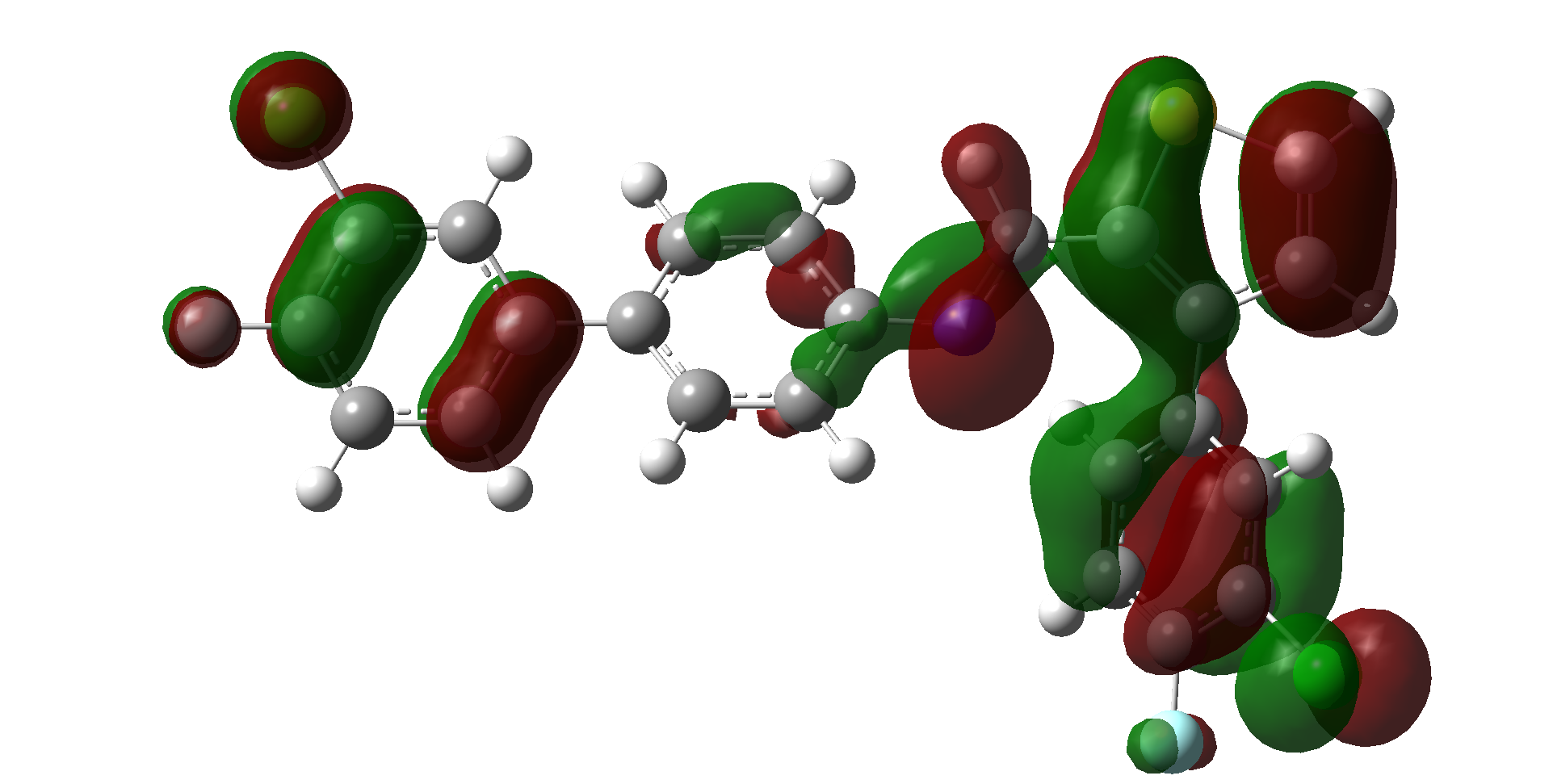  HOMO-2 | 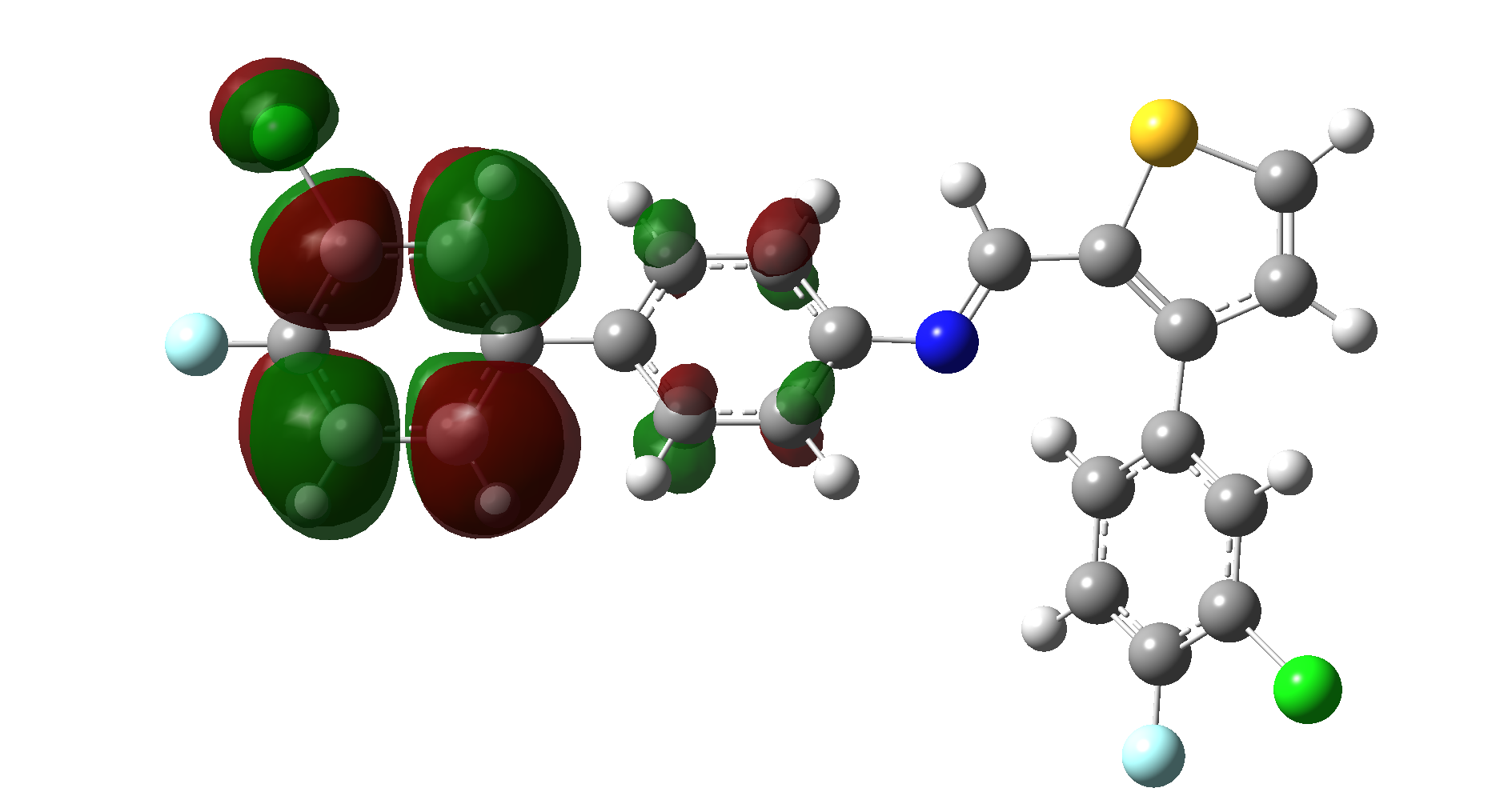  LUMO+2 |
| 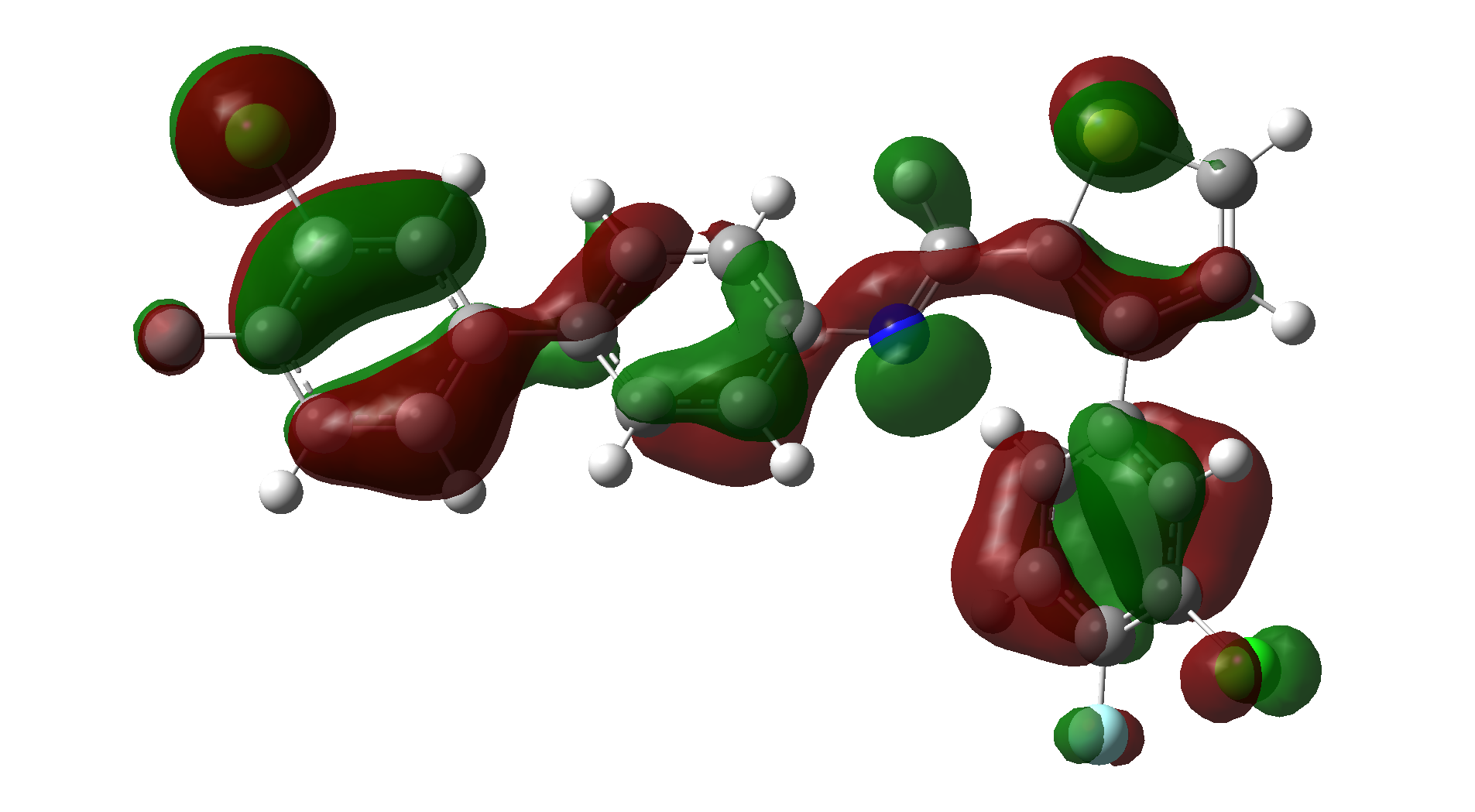  HOMO-3 | 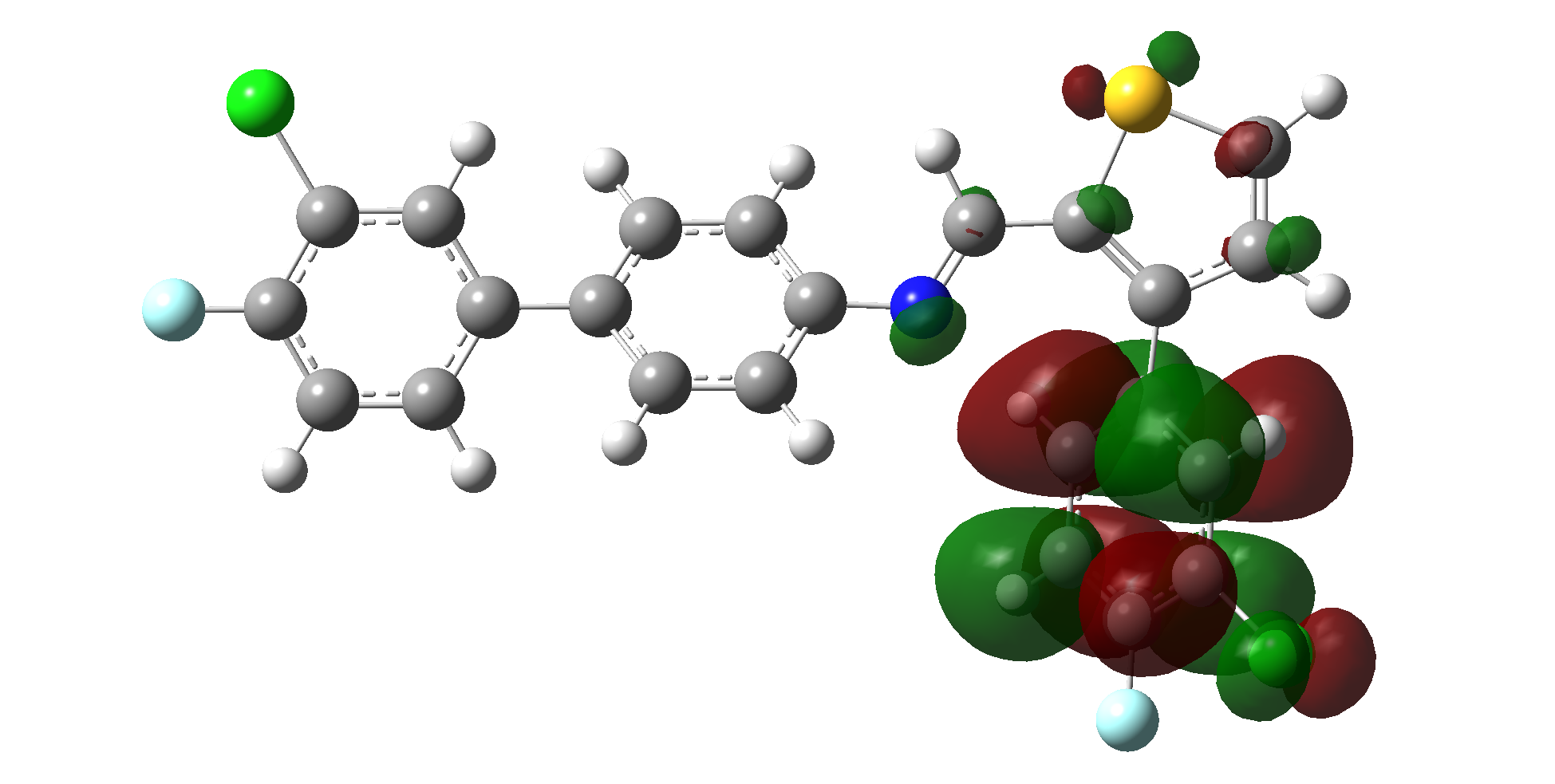    LUMO+3 |

| **3h** | |
| --- | --- |
| 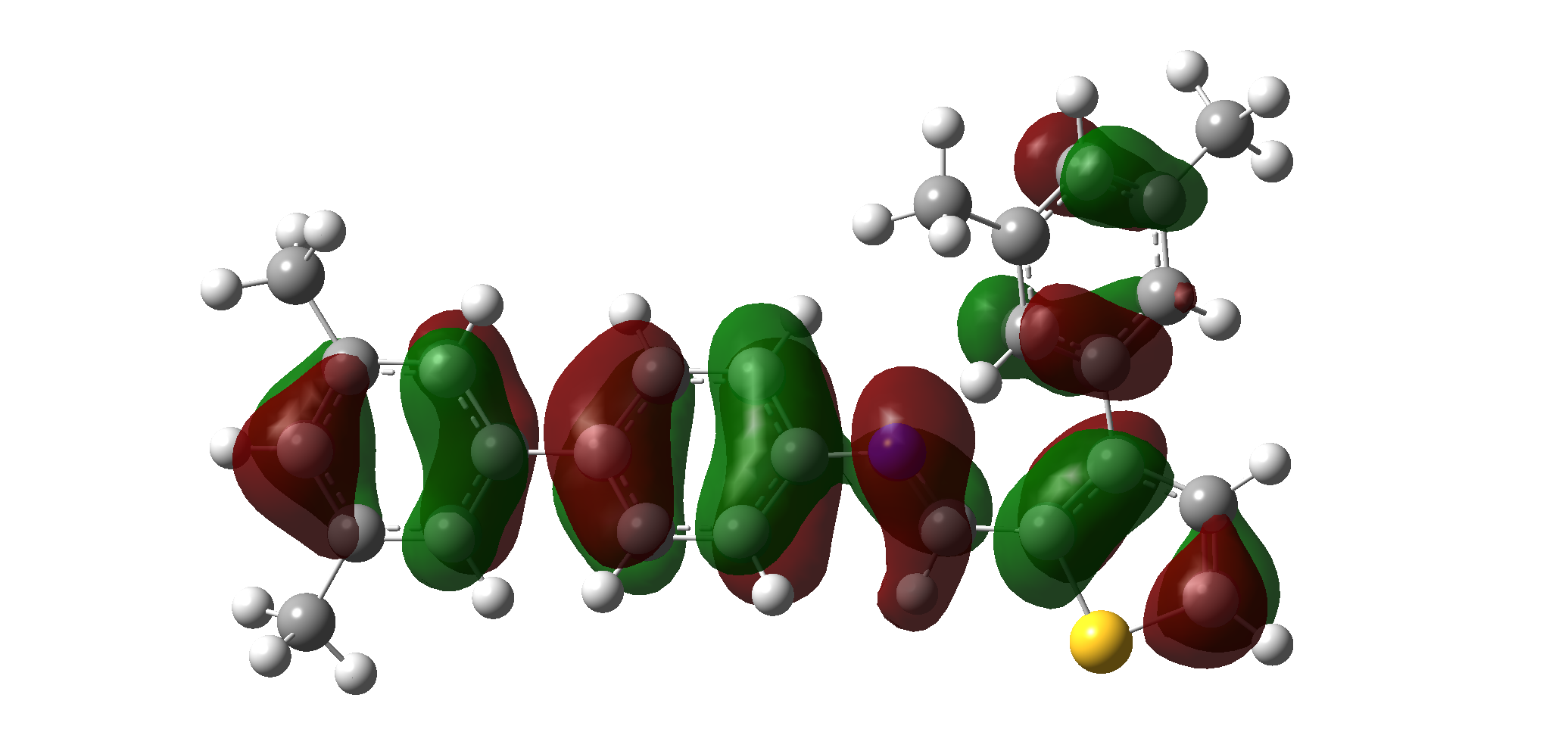  HOMO | 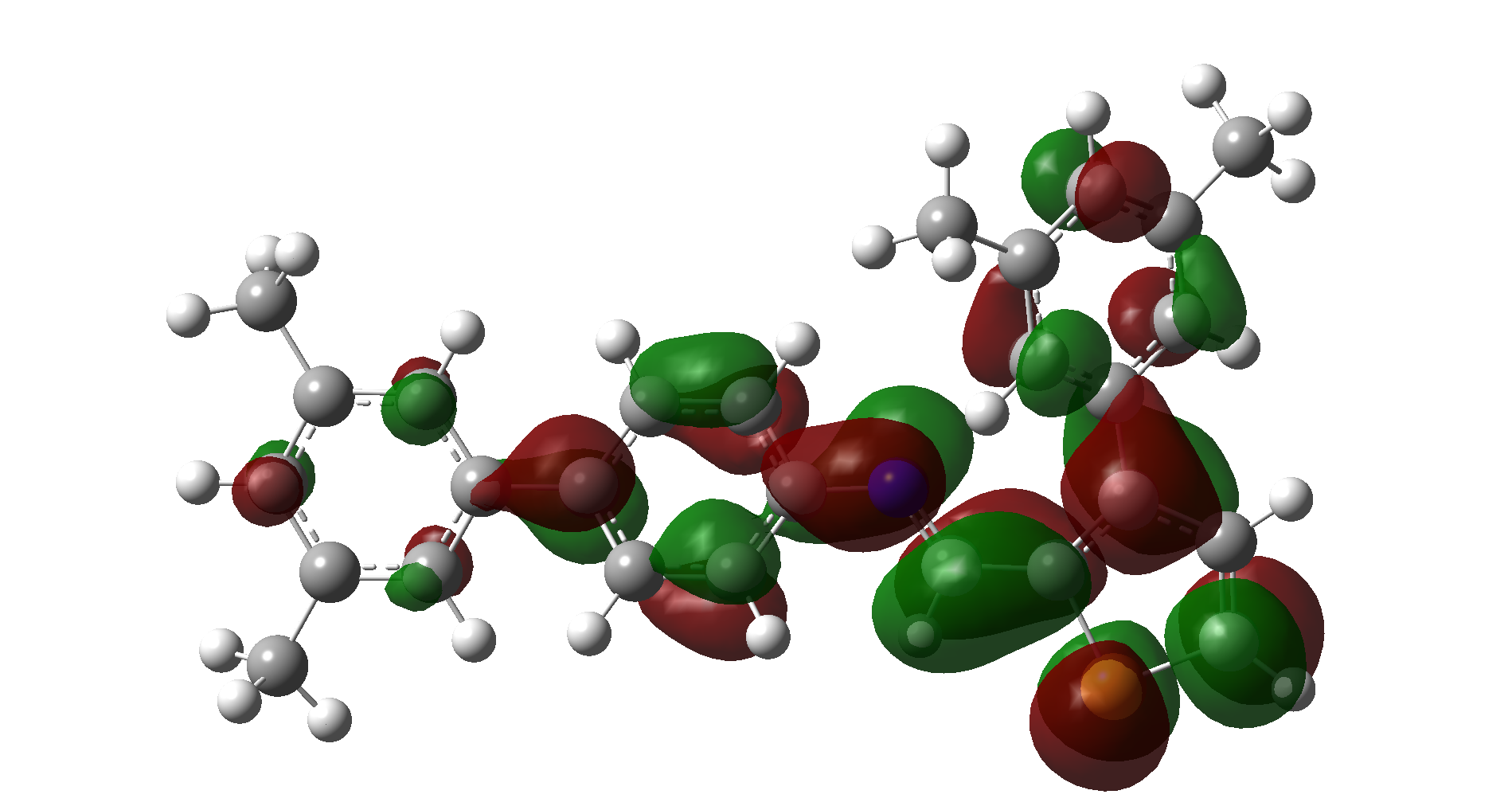  LUMO |
| 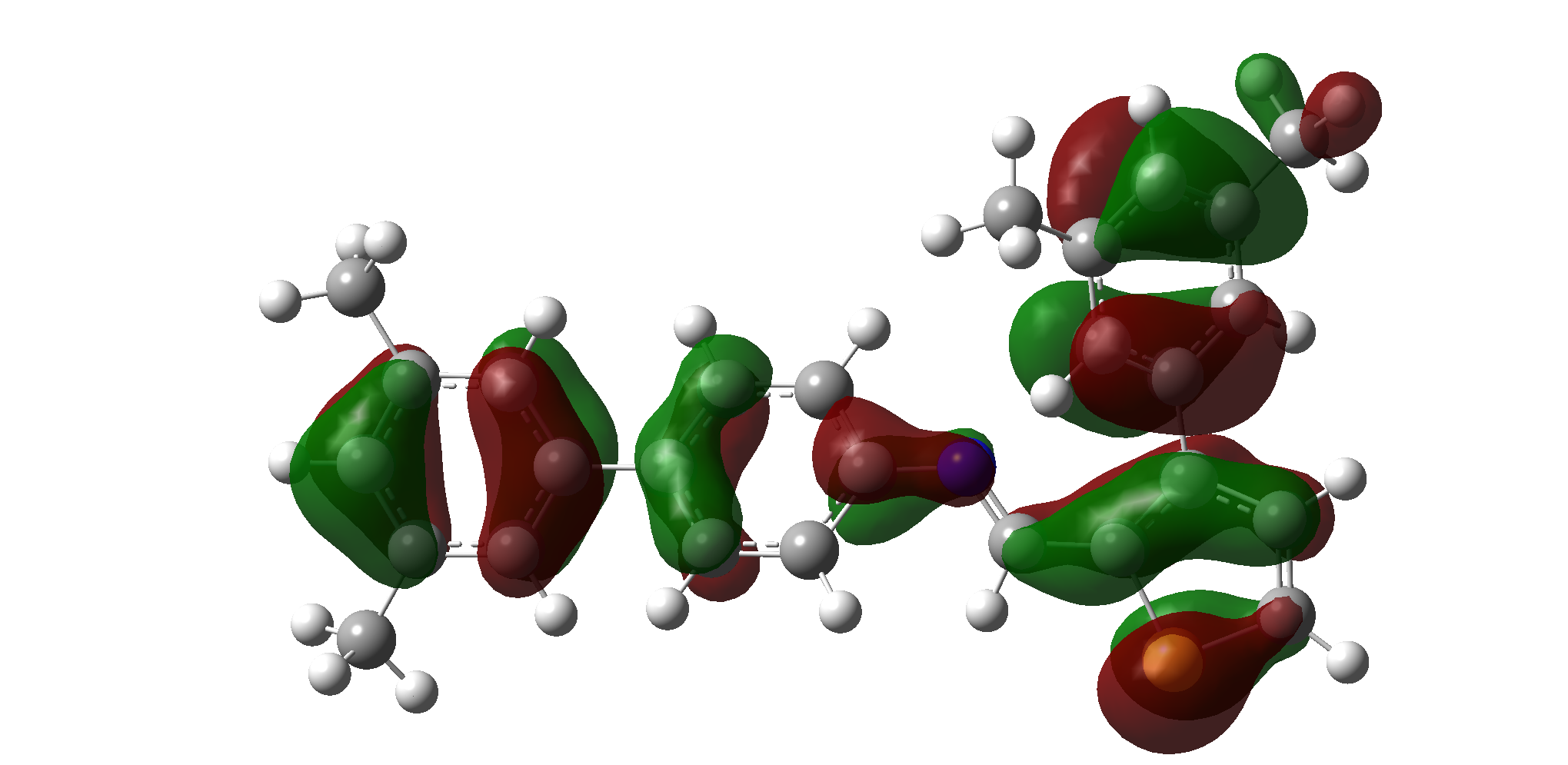  HOMO-1 | 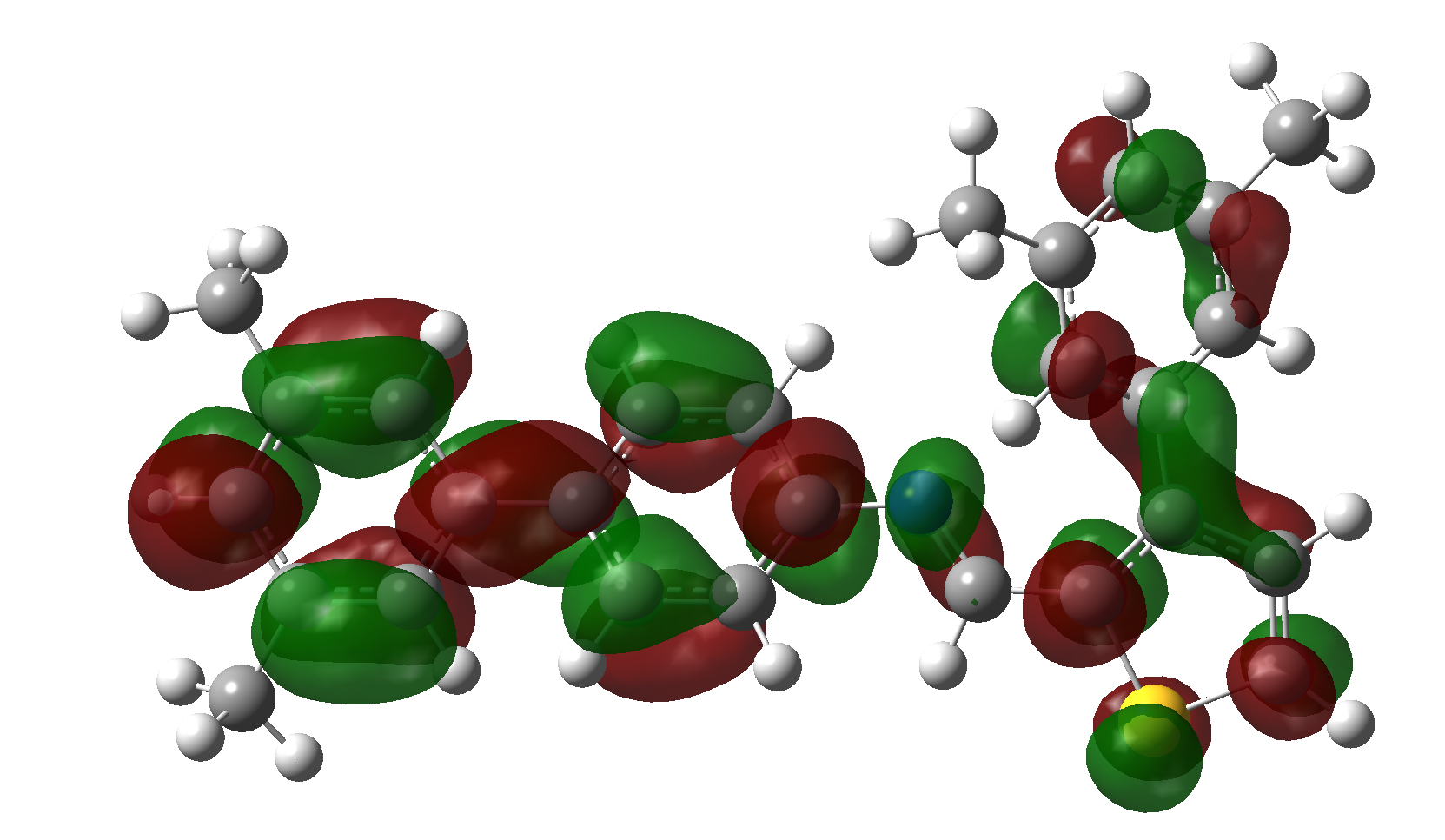  LUMO+1 |
| 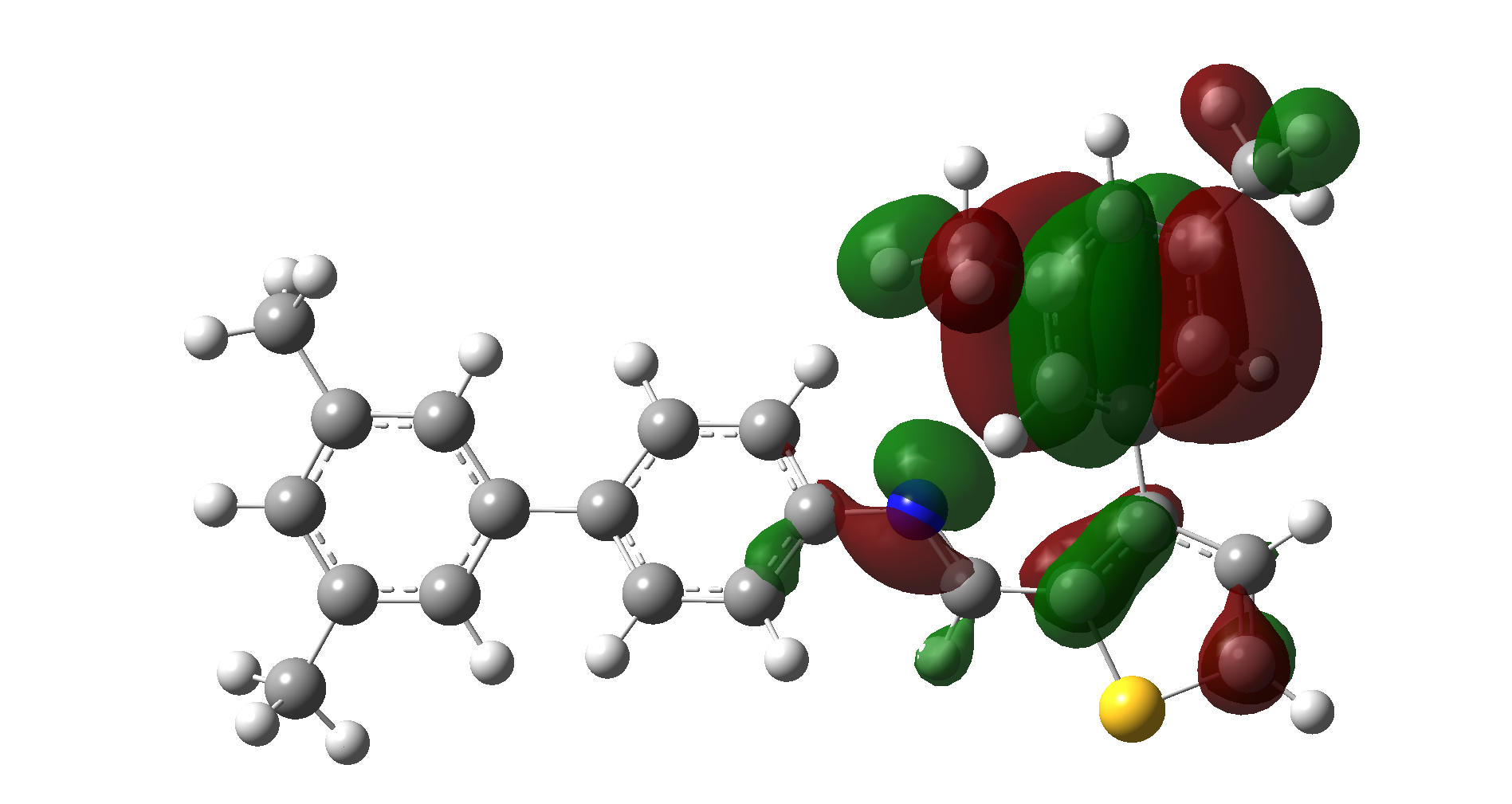  HOMO-2 | 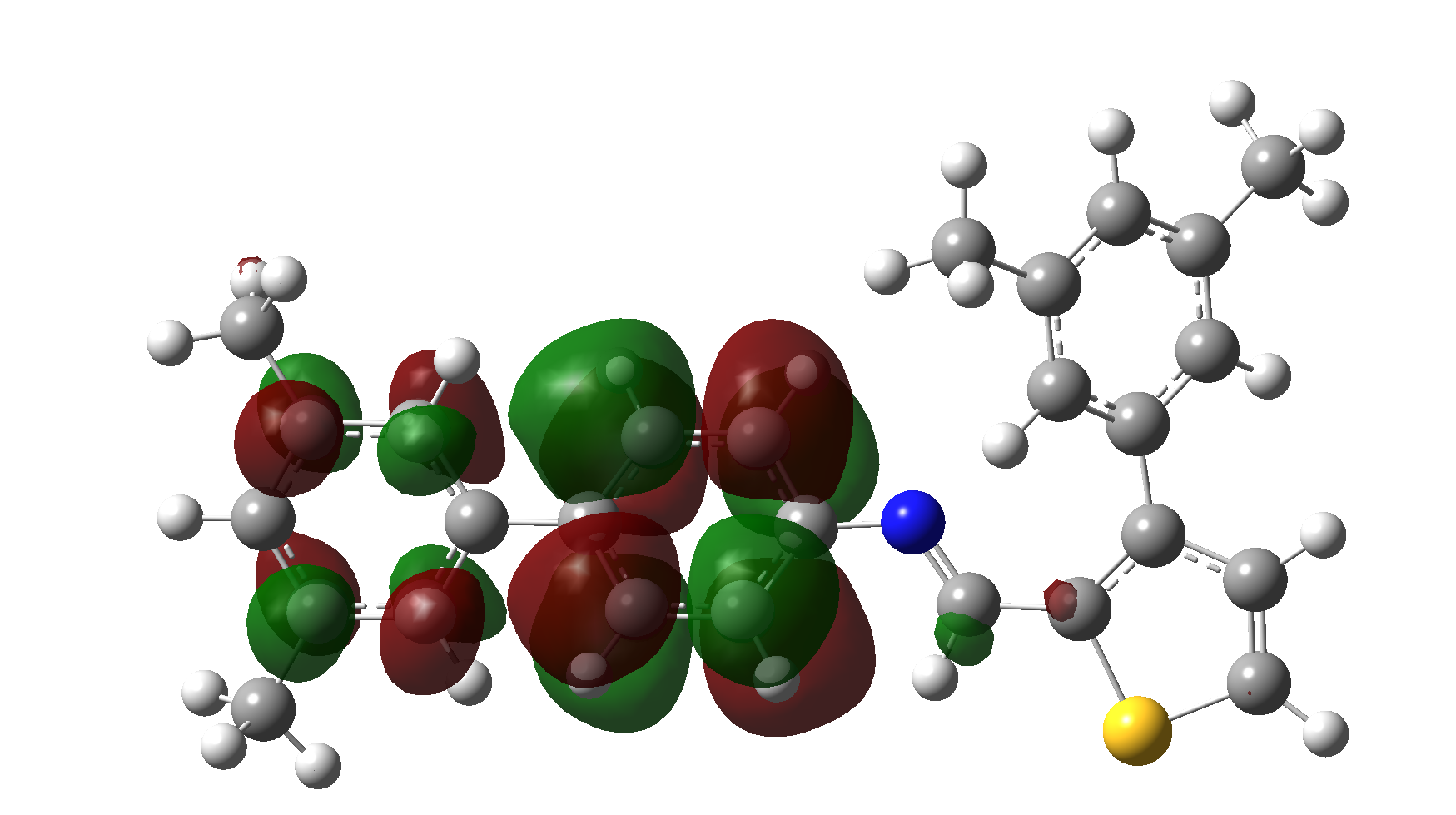  LUMO+2 |
| 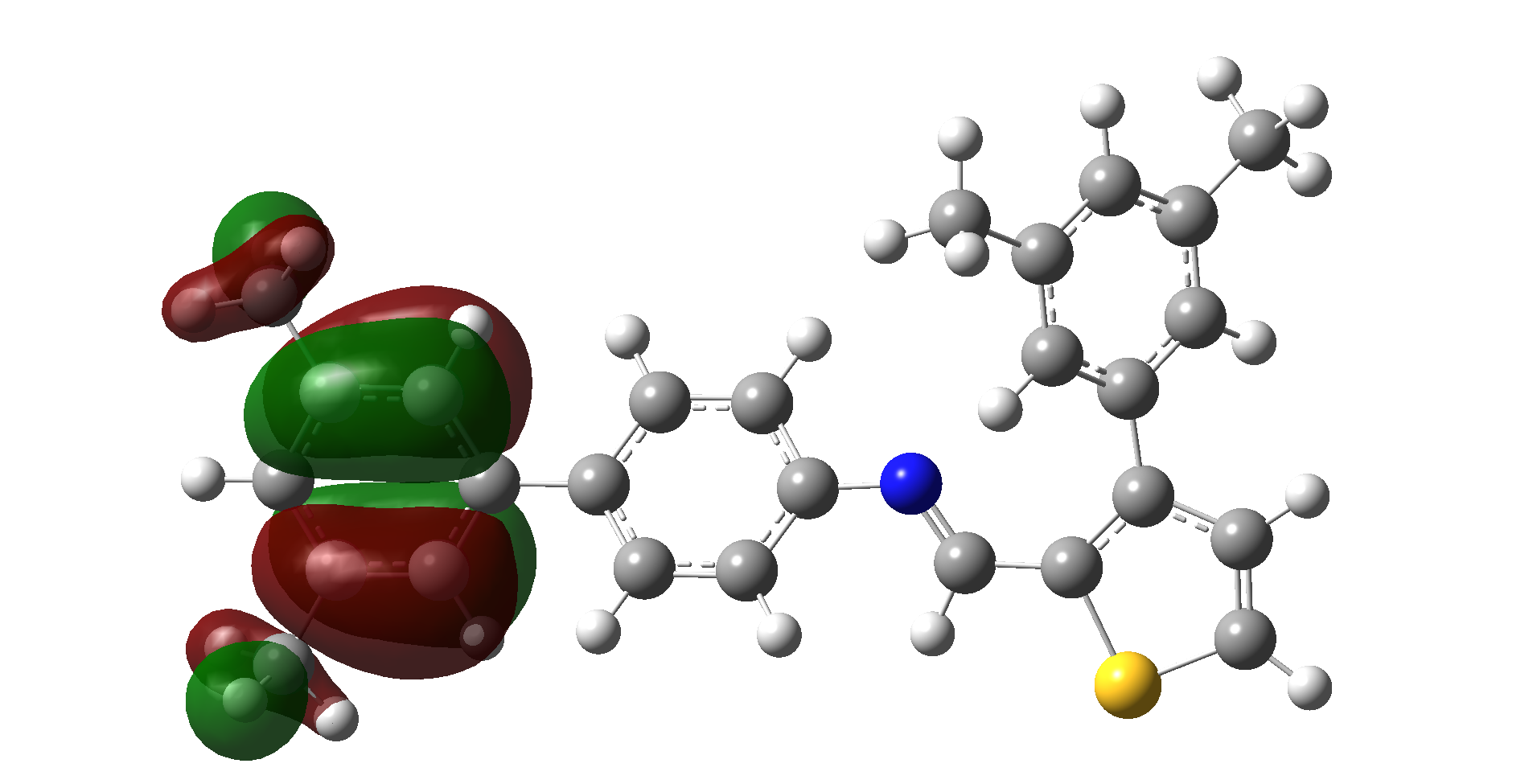  HOMO-3 | 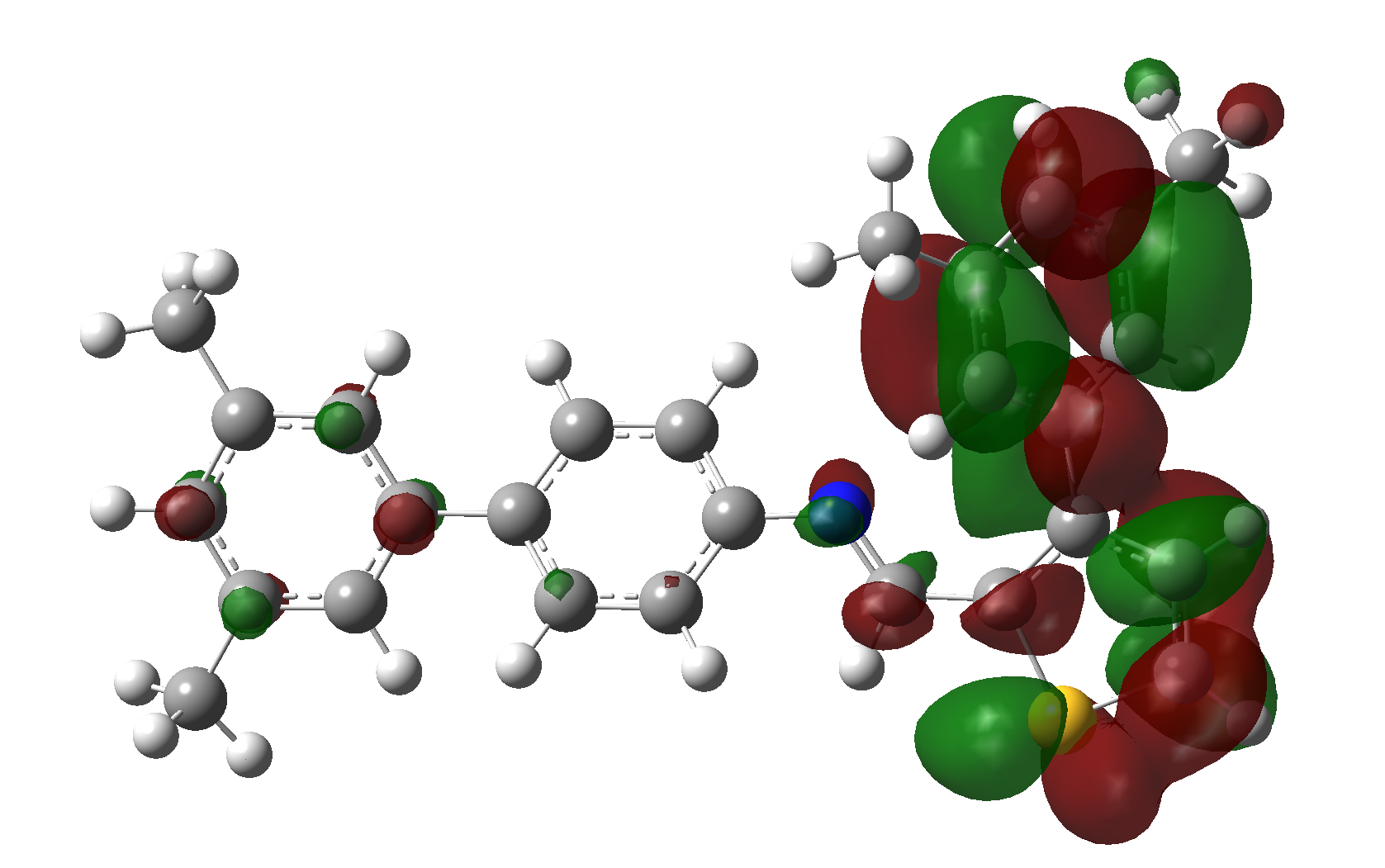  LUMO+3 |

| **3i** | |
| --- | --- |
| 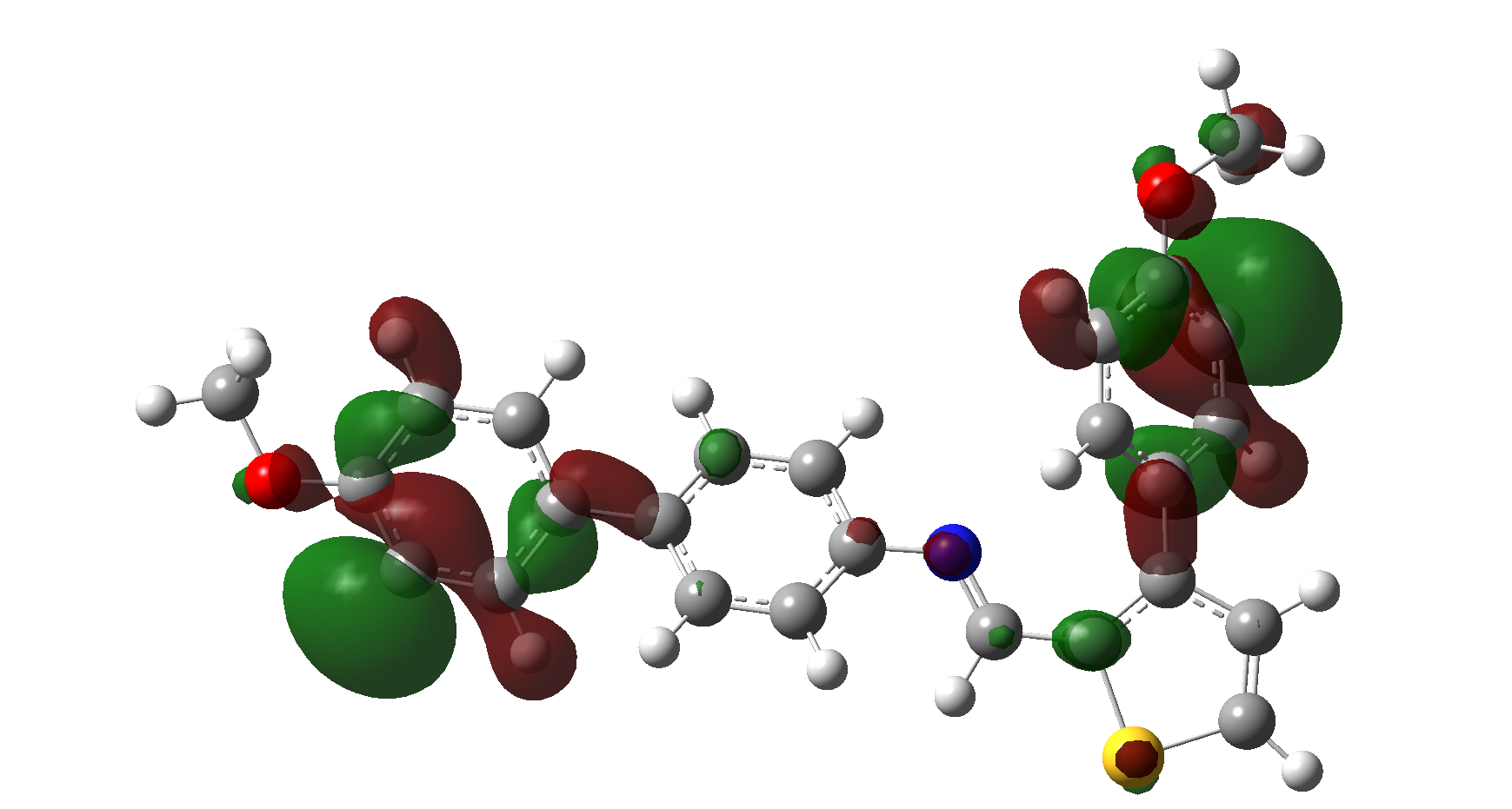  HOMO | 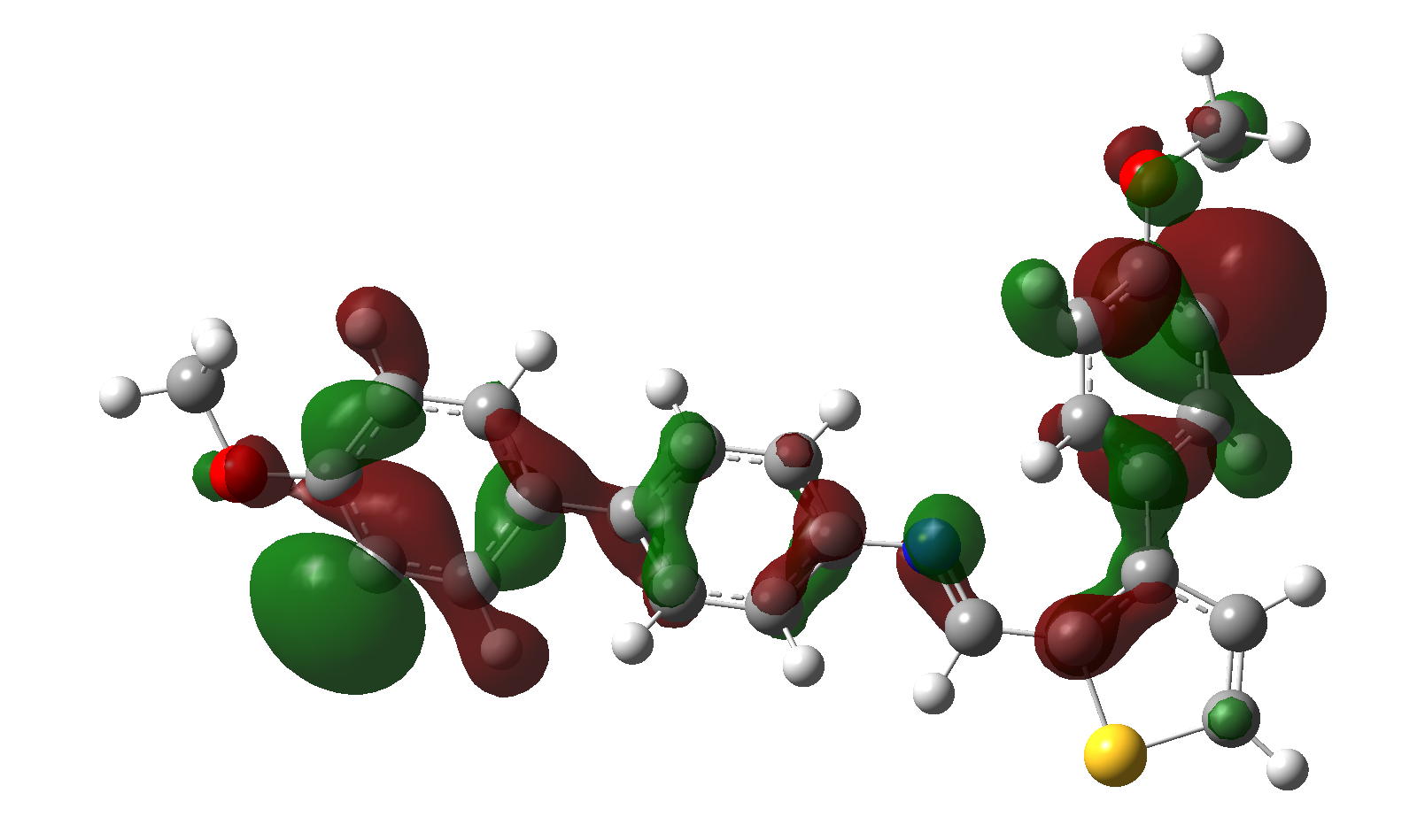  LUMO |
| 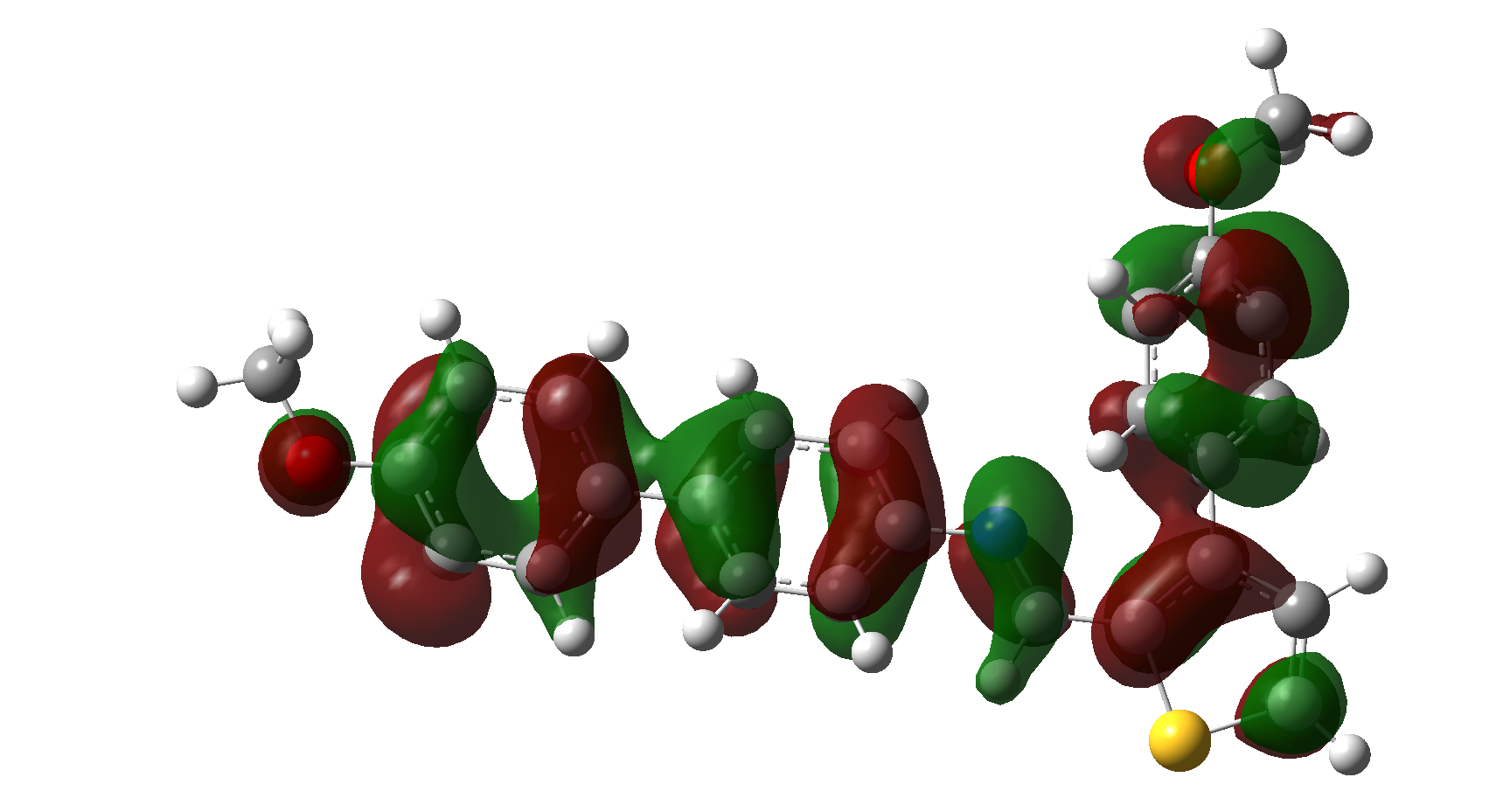  HOMO-1 | 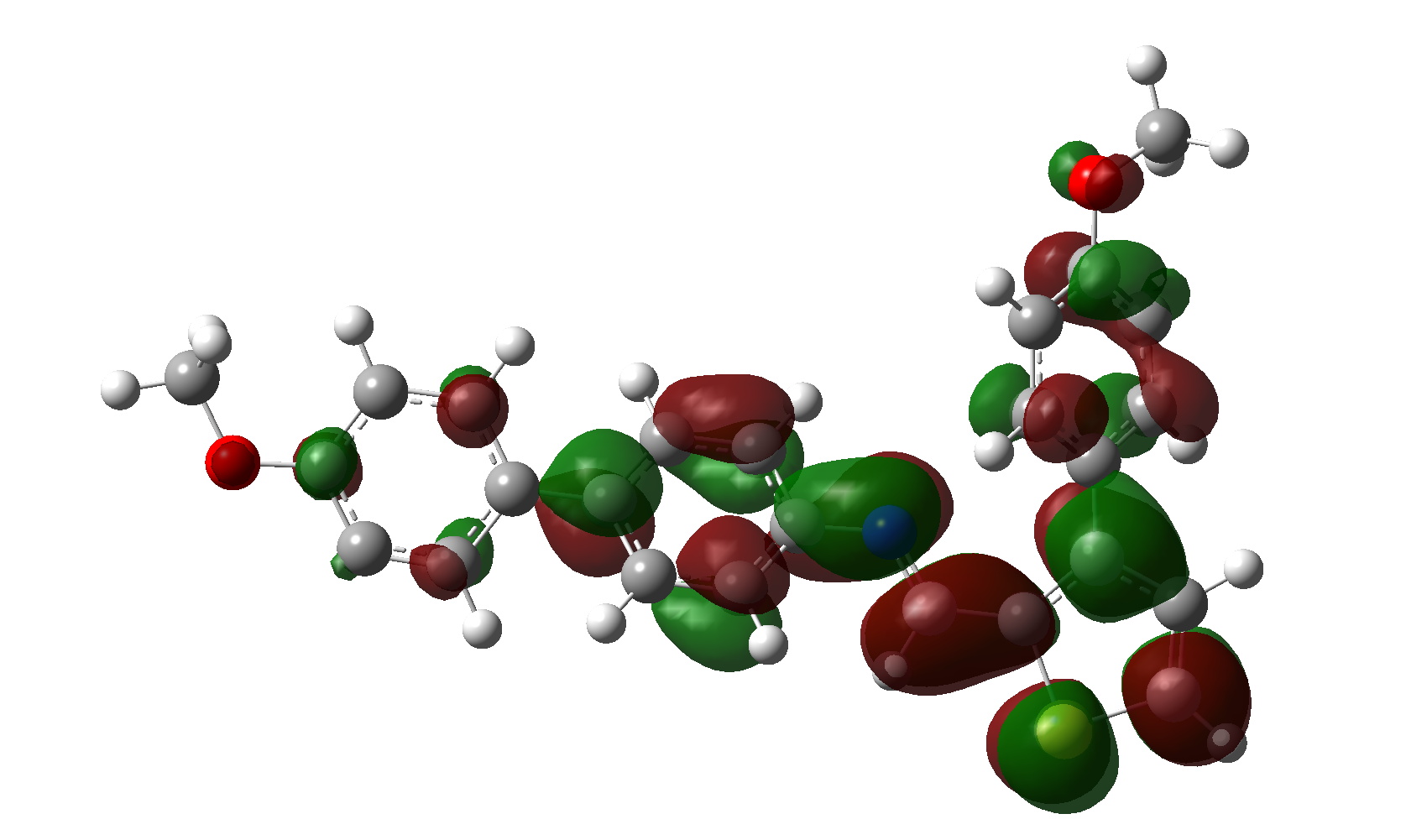  LUMO+1 |
| 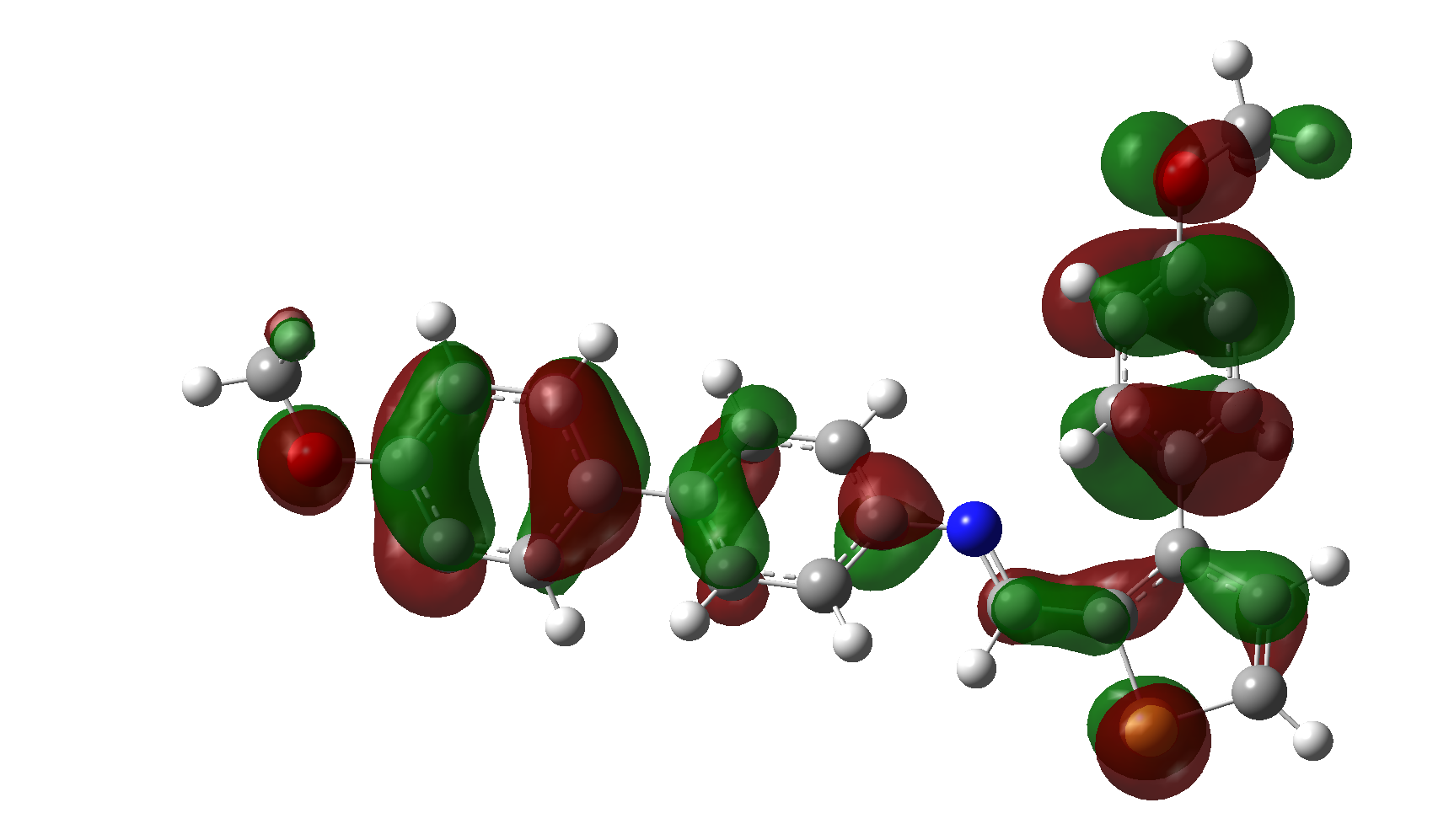  HOMO-2 | 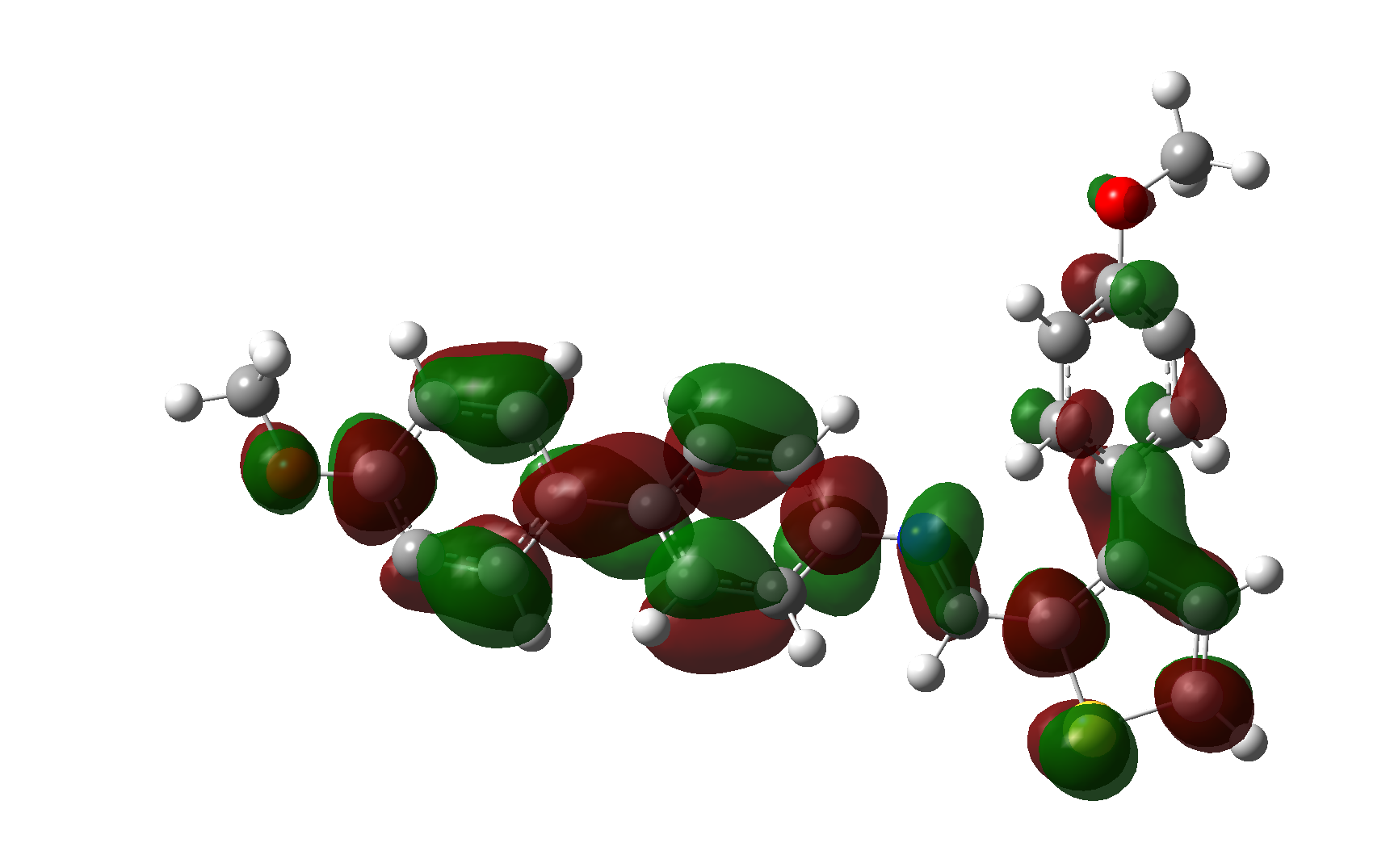  LUMO+2 |
| 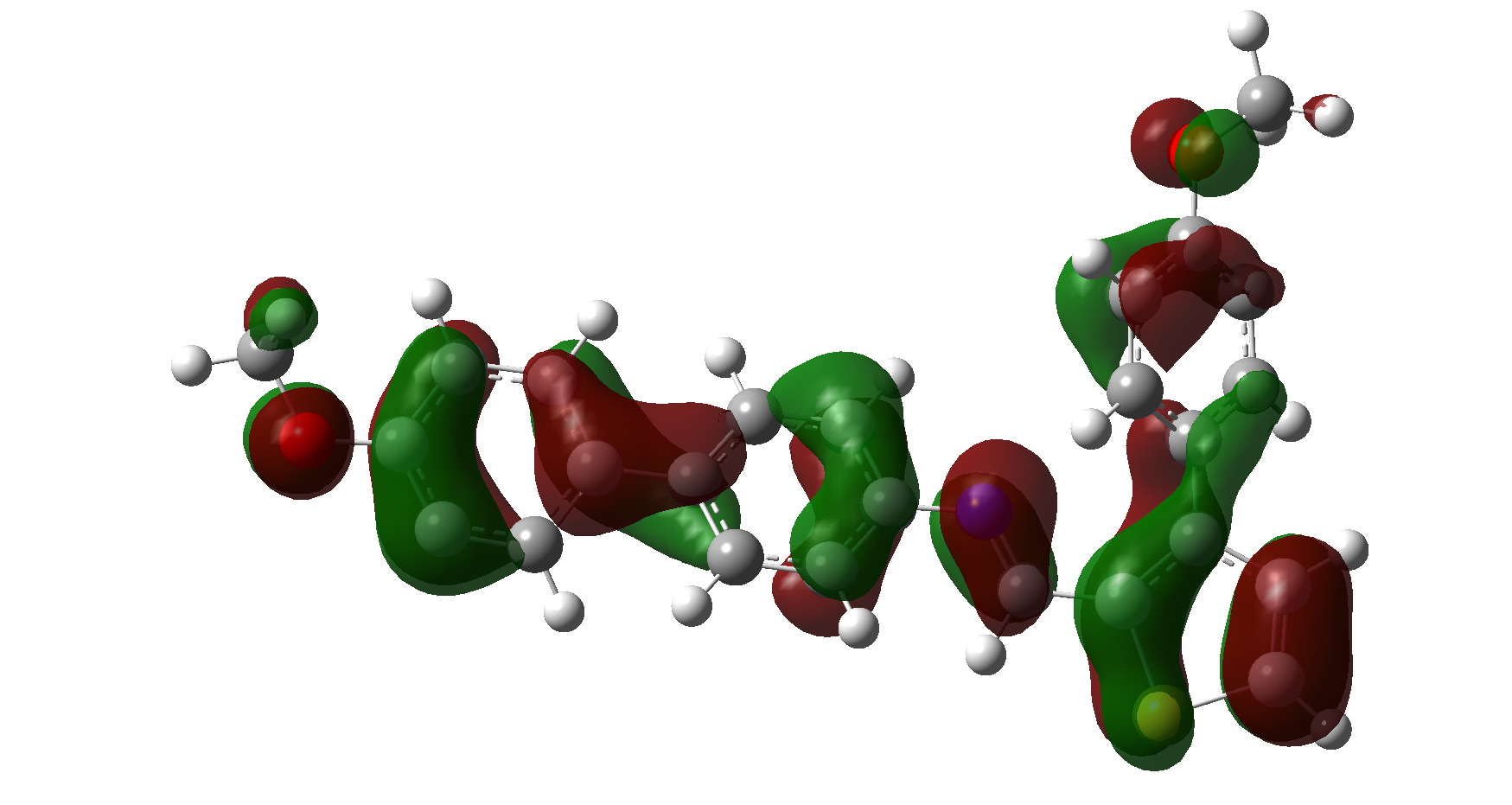  HOMO-3 | 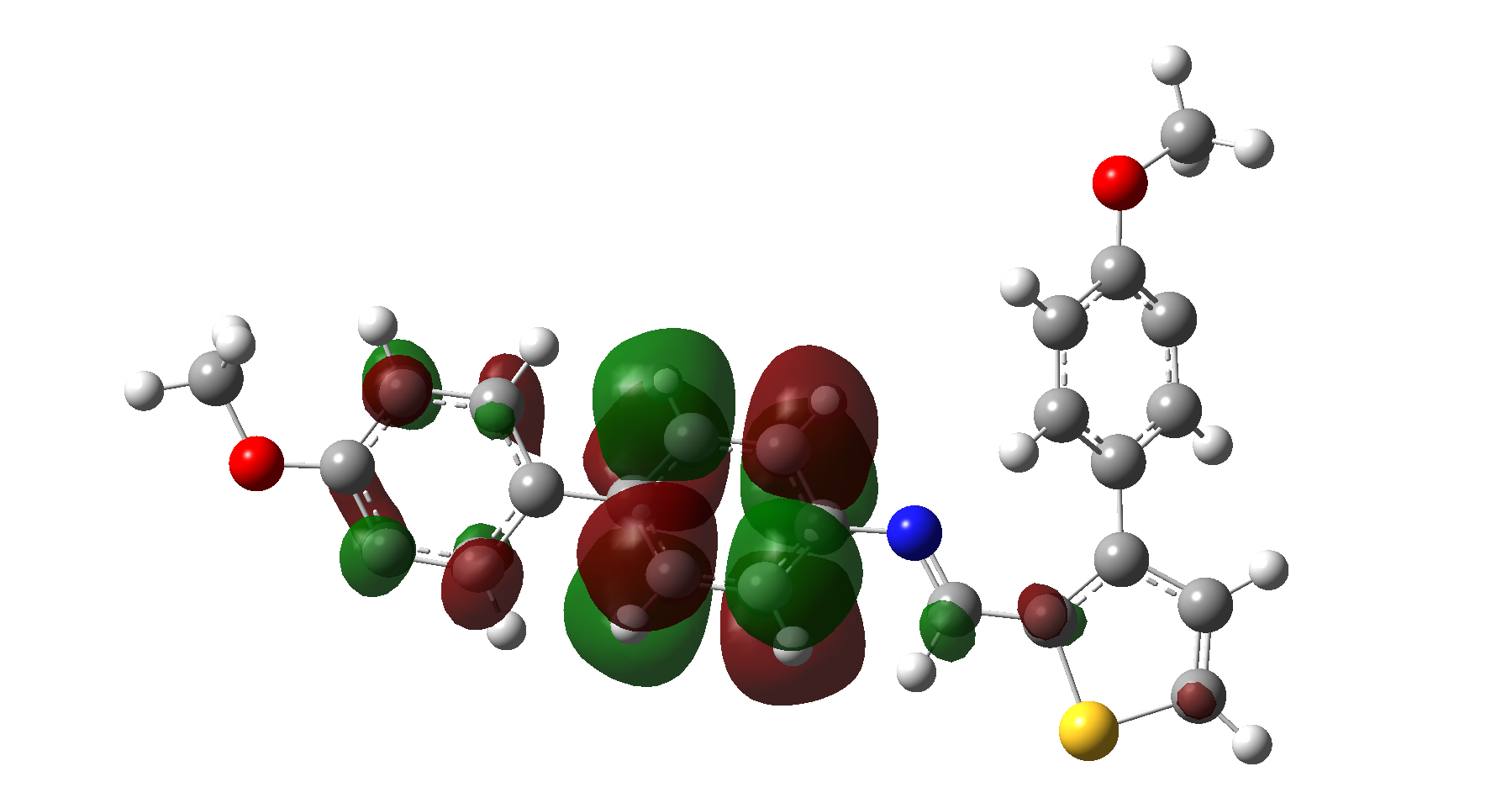  LUMO+3 |

**Figure S1**: HOMO, HOMO-1, HOMO-2, HOMO-3 and LUMO, LUMO+1, LUMO+2, LUMO+3 surfaces of **3b-3i**
